# Fungal Depsides—Naturally Inspiring Molecules: Biosynthesis, Structural Characterization, and Biological Activities

**DOI:** 10.3390/metabo11100683

**Published:** 2021-10-05

**Authors:** Sabrin R. M. Ibrahim, Alaa Sirwi, Basma G. Eid, Shaimaa G. A. Mohamed, Gamal A. Mohamed

**Affiliations:** 1 Preparatory Year Program, Batterjee Medical College, Jeddah 21442, Saudi Arabia; 2 Department of Pharmacognosy, Faculty of Pharmacy, Assiut University, Assiut 71526, Egypt; 3 Department of Natural Products and Alternative Medicine, Faculty of Pharmacy, King Abdulaziz University, Jeddah 21589, Saudi Arabia; asirwi@kau.edu.sa (A.S.); gahussein@kau.edu.sa (G.A.M.); 4 Department of Pharmacology and Toxicology, Faculty of Pharmacy, King Abdulaziz University, Jeddah 21589, Saudi Arabia; beid@kau.edu.sa; 5 Faculty of Dentistry, British University, Suez Desert Road, El Sherouk City 11837, Egypt; shaimaag1973@gmail.com; 6 Department of Pharmacognosy, Faculty of Pharmacy, Al-Azhar University, Assiut Branch, Assiut 71524, Egypt

**Keywords:** fungi, depsides, biosynthesis, spectral data, biological activities

## Abstract

Fungi represent a huge reservoir of structurally diverse bio-metabolites. Although there has been a marked increase in the number of isolated fungal metabolites over the past years, many hidden metabolites still need to be discovered. Depsides are a group of polyketides consisting of two or more ester-linked hydroxybenzoic acid moieties. They possess valuable bioactive properties, such as anticancer, antidiabetic, antibacterial, antiviral, anti-inflammatory, antifungal, antifouling, and antioxidant qualities, as well as various human enzyme-inhibitory activities. This review provides an overview of the reported data on fungal depsides, including their sources, biosynthesis, physical and spectral data, and bioactivities in the period from 1975 to 2020. Overall, 110 metabolites and more than 122 references are confirmed. This is the first review of these multi-faceted metabolites from fungi.

## 1. Introduction

**Figure 1:**
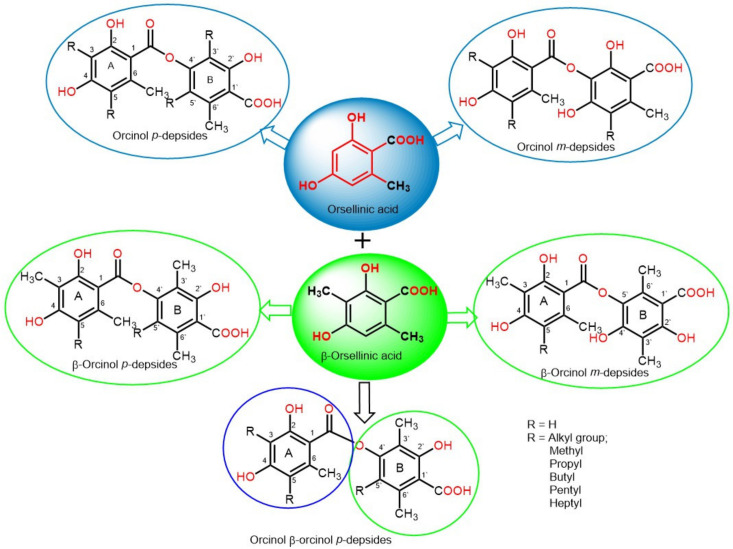
Basic structures of orcinol *para*depsides, orcinol *meta*depsides, β-orcinol *para*depsides, β-orcinol *meta*depsides, and mixed orcinol β-orcinol depsides.

Fungi are widespread cosmopolitan organisms that represent the second-largest class of organisms after insects [1]. For decades, they have been seen as harmful, causing health hazards and spoiling foods. Today, the view on fungi has altered to take into account their advantageous effects, which have become apparent in biotechnological and industrial fields, as well as the production of structurally unique and life-saving metabolites [2,3,4,5]. In the past decades, the number of bioactive metabolites isolated from fungi has been rapidly increased due to their wide diversity of ecological and environmental niches across the globe, including marine, terrestrial, and water environments where they function as pathogens, symbionts, and saprobes [6,7]. Fungi-derived metabolites have made remarkable contributions to the process of drug discovery [8,9,10,11,12,13,14,15,16]. They have been used as antibiotics, herbicides, pesticides, anti-infectives, immuno-suppressants, and anticancer agents [6,7,17]. Depsides are simple polyketides that are formed by the condensation of two or more hydroxybenzoic acid moieties via ester linkage; the COOH group of one molecule is esterified with a phenolic OH group of the second molecule. They could be β-orcinol (β-orsellinic acid) or orcinol (orsellinic acid) derivatives, relying on the existence of the C_3_ methyl group on both rings (Figure 1). The ring with an ester-carbonyl is referred to as ring A and the other as ring B. Their major structural variations are the attached alkyl chains’ length, the degree of chain oxidation, and the degree of methylation of OH and COOH groups [18]. The OH groups usually exist at the aromatic carbons, C-3′/C-4′/C-2 or C-4, and other oxygenated substituents are usually connected to the skeleton, such as carboxyl and methoxyl substituents [19].

**Figure 2:**
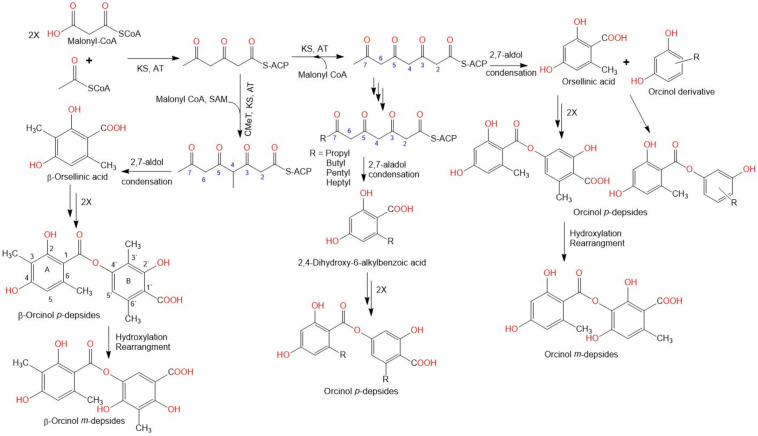
Biosynthesis of depsides [18,28,29,30,31,32,33].

**Figure 3:**
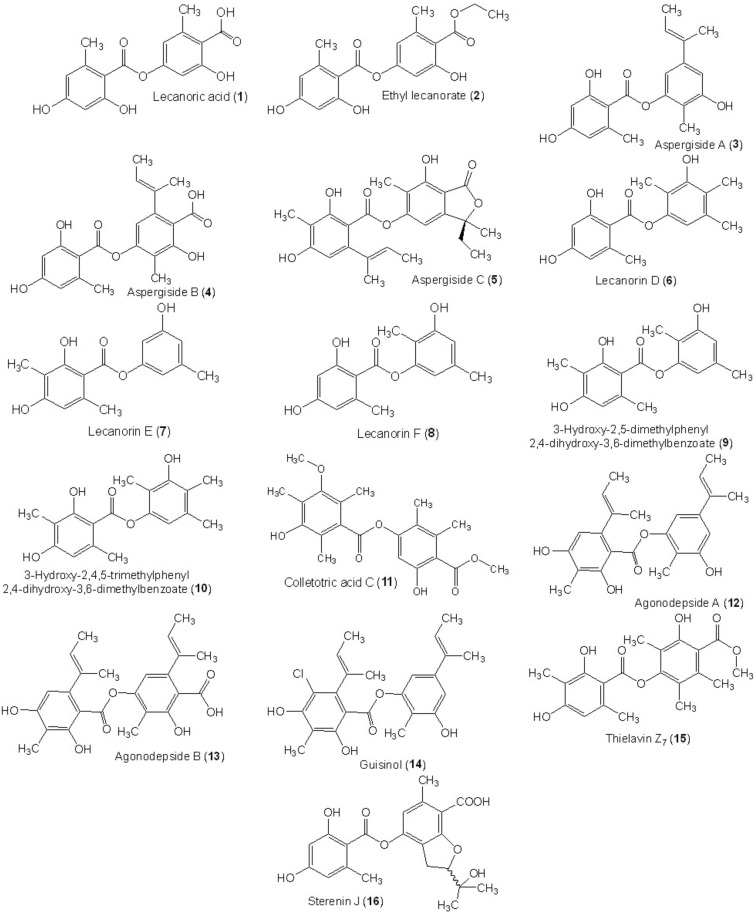
Chemical structures of di-depsides **1**–**16**.

**Figure 4:**
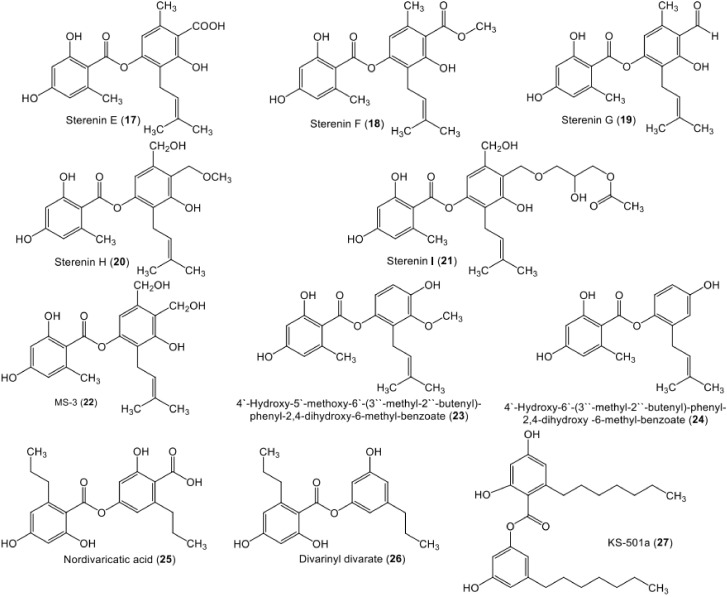
Chemical structures of di-depsides **17**–**27**.

**Figure 5:**
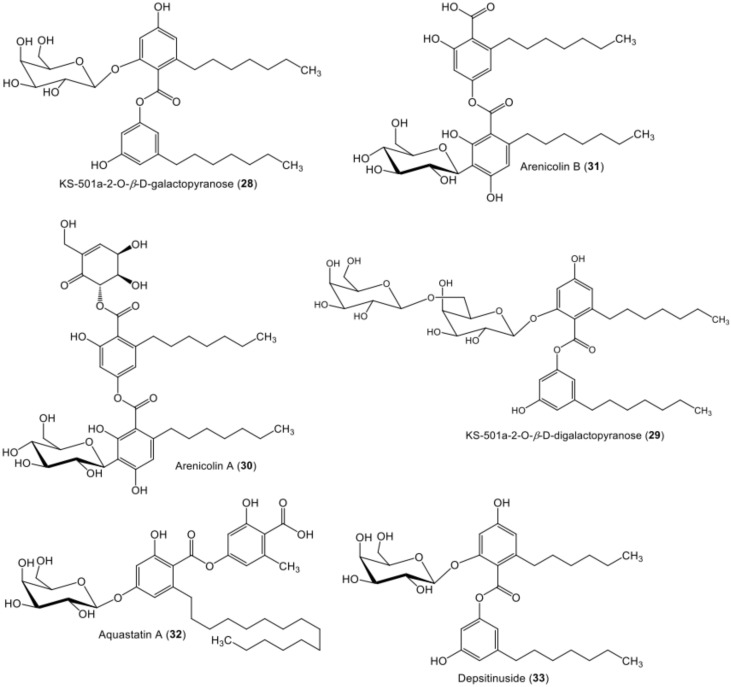
Chemical structures of sugar-containing di-depsides **28**–**33**.

**Figure 6:**
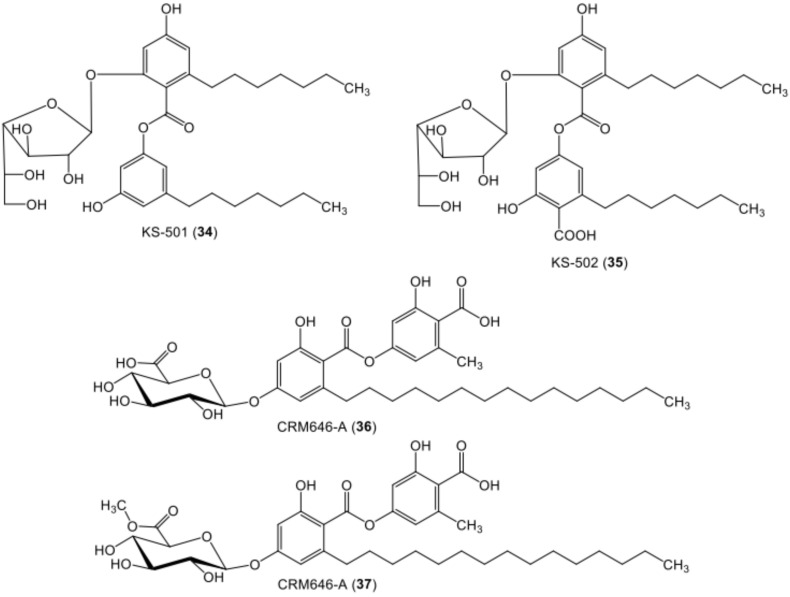
Chemical structures of sugar-containing di-depsides **34**–**37**.

**Figure 7:**
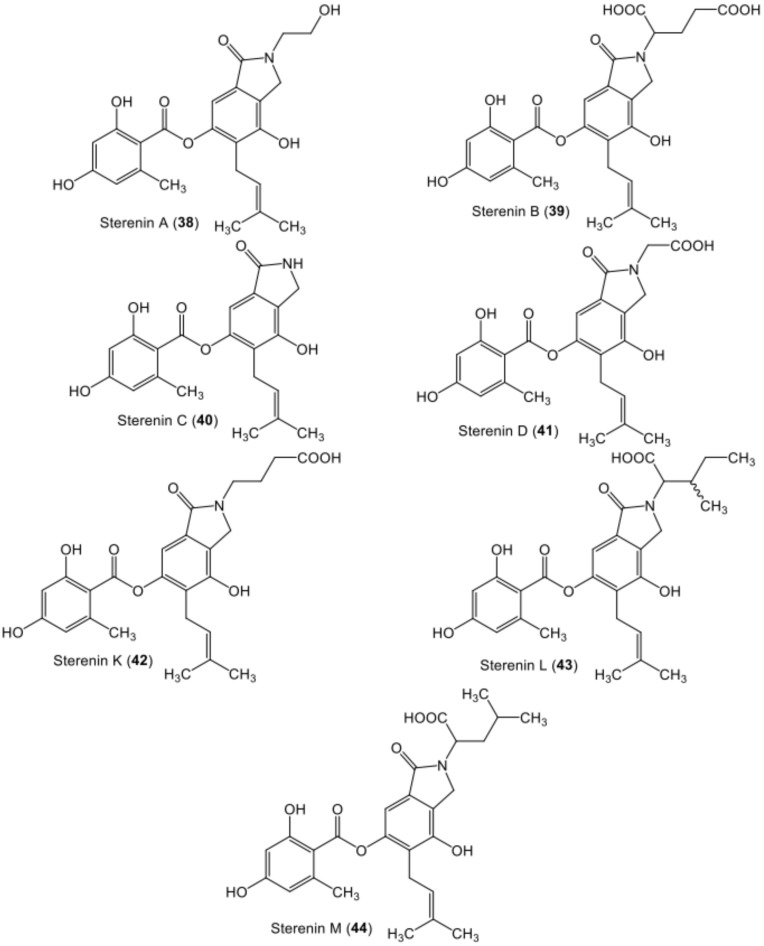
Chemical structures of nitrogen-containing di-depsides **38**–**44**.

**Figure 8:**
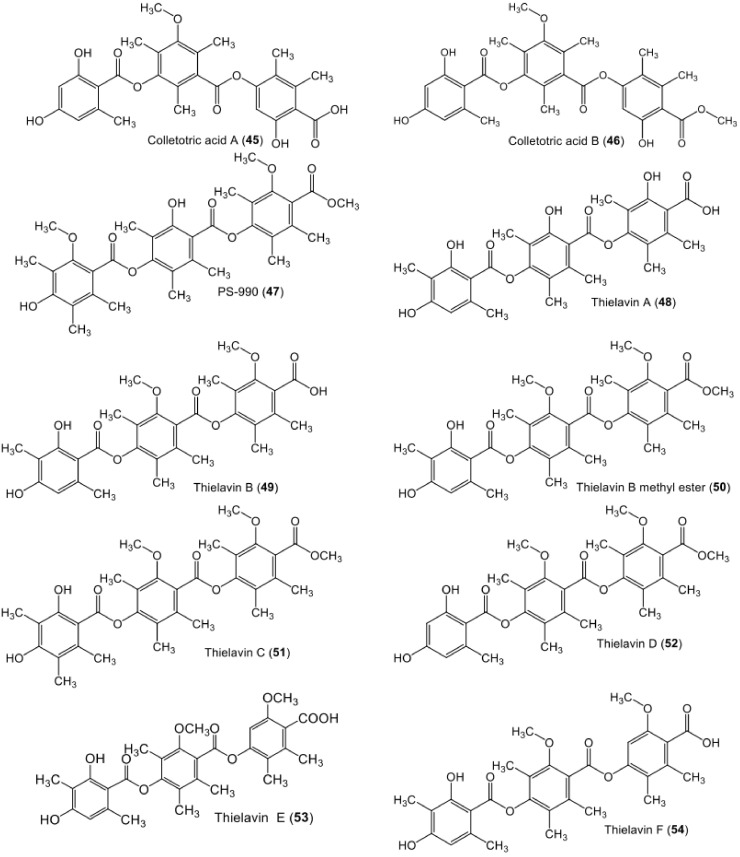
Chemical structures of tri-depsides **45**–**54**.

**Figure 9:**
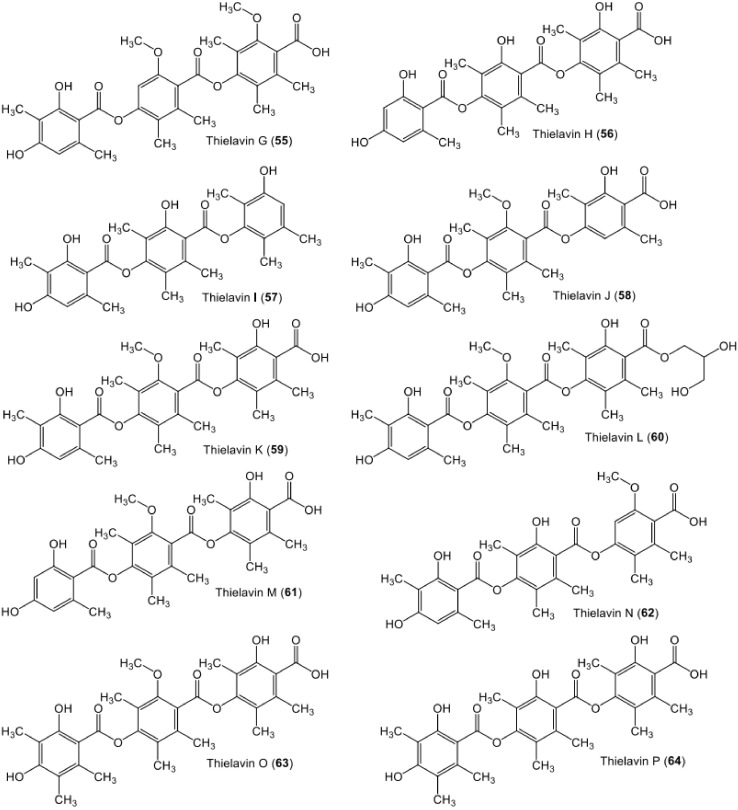
Chemical structures of tri-depsides **55**–**64**.

**Figure 10:**
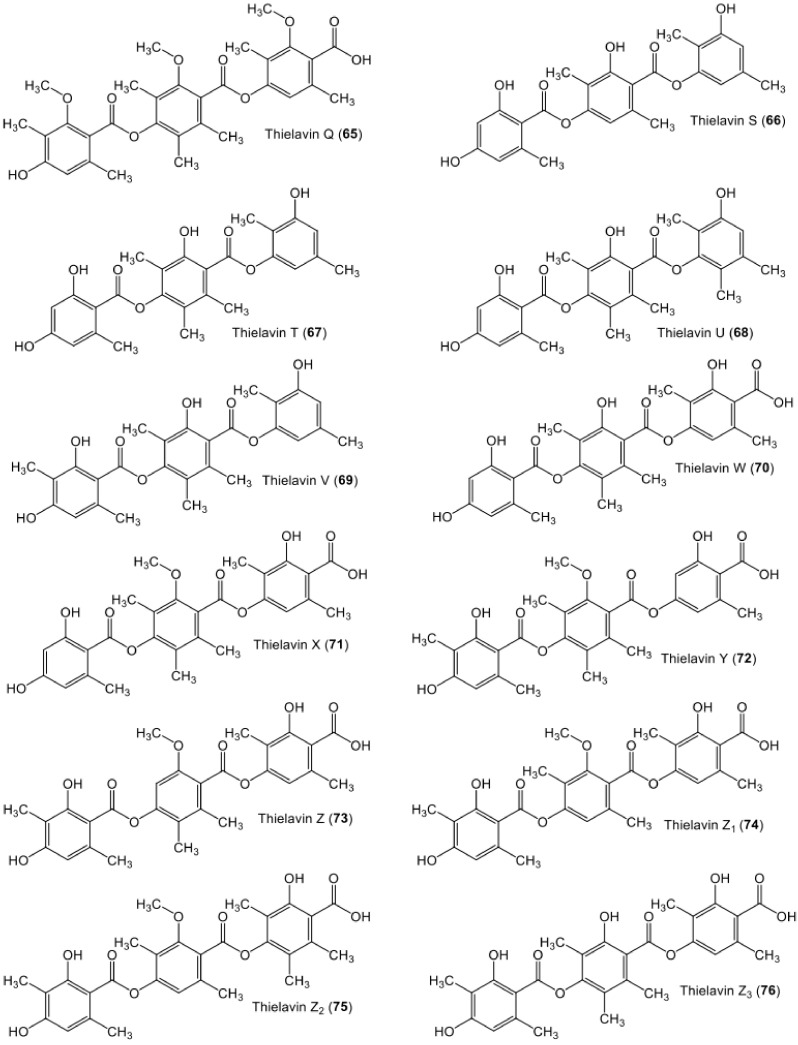
Chemical structures of tri-depsides **65**–**76**.

**Figure 11:**
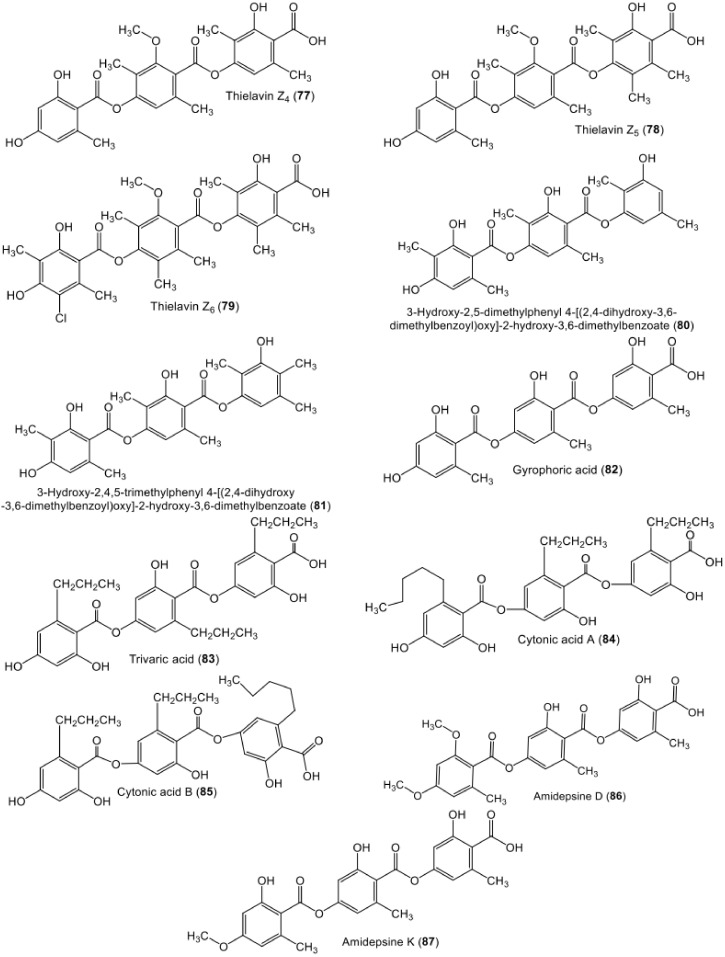
Chemical structures of tri-depsides **77**–**87**.

**Figure 12:**
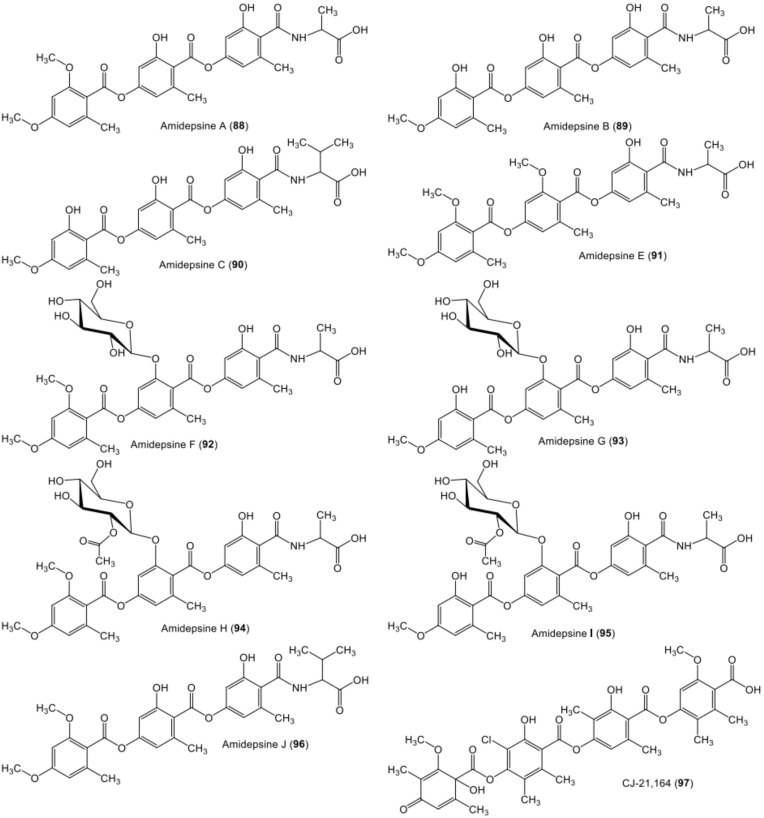
Chemical structures of nitrogen- and sugar-containing tri-depsides **88**–**96** and tetra-depside **97**.

**Figure 13:**
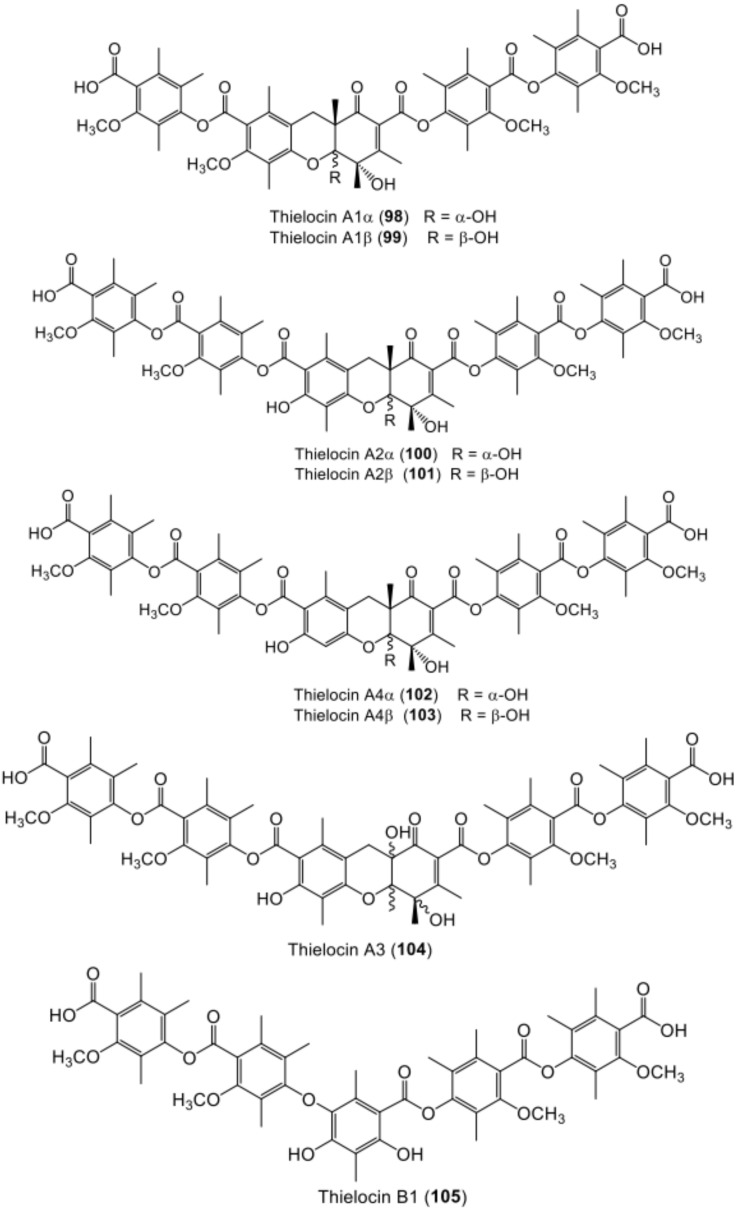
Chemical structures of terta- and penta-depside derivatives **98**–**105**.

**Figure 14:**
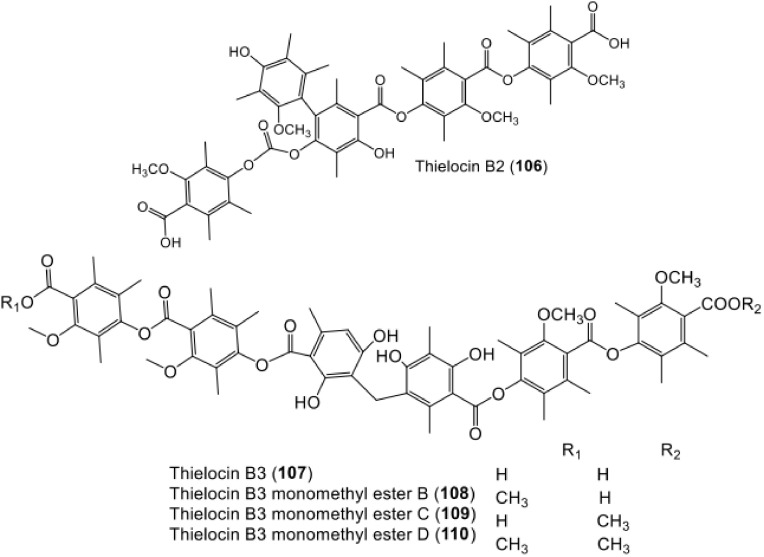
Chemical structures of penta- and hexa-depside **106**–**110**.

**Figure 15:**
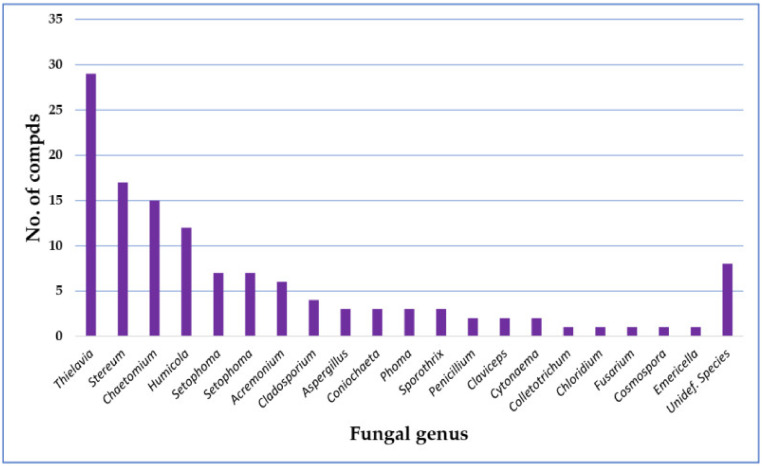
Numbers of depsides isolated from different fungal genera.

**Figure 16:**
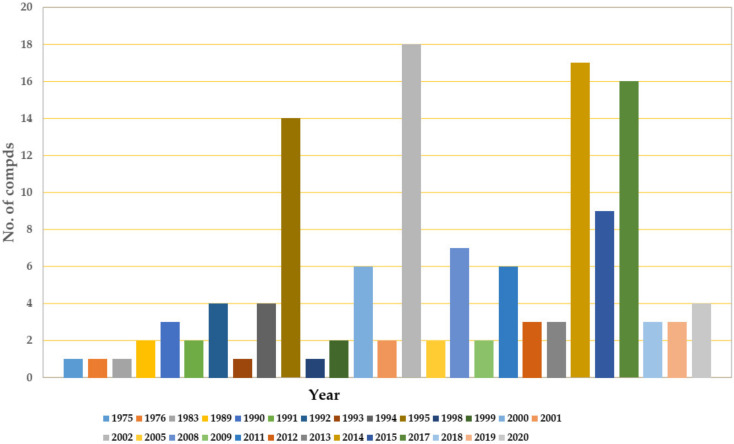
Annual numbers of isolated depsides, from 1975 to 2020.

**Figure 17:**
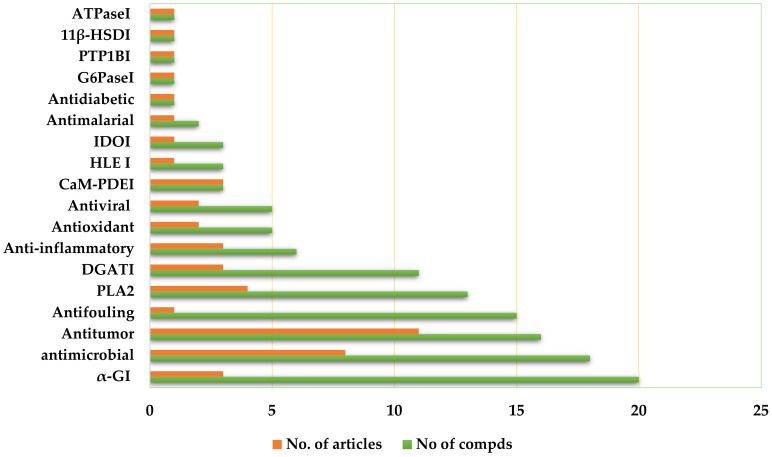
Biological activities of isolated depsides and the number of articles.

Depsides are common lichen metabolites [20,21,22]. However, they have also been reported in some higher plants and fungi [19,23,24,25,26,27]. Contrary to the lichen depsides, fungal depsides are not widely distributed and are isolated only from a restricted number of fungi (Table 1). It was reported that depsides possess remarkable bioactivities: anticancer, anti-diabetic, antibacterial, antiviral, anti-inflammatory, antifungal, antifouling, antioxidant, and various enzyme inhibitory activities. Therefore, these metabolites are of considerable importance as a prospective lead motif for medicinal chemistry. Our review of the literature indicated that there is currently no review on fungal depsides. Herein, 110 depsides reported in the literature from fungal sources have been listed, with a summary of their biosynthesis, physical constants, spectral data, sources, bioactivities, and references (Table 1, Table 2 and Table S1, Figure 2, Figure 3, Figure 4, Figure 5, Figure 6, Figure 7, Figure 8, Figure 9, Figure 10, Figure 11, Figure 12, Figure 13, Figure 14, Figure 15, Figure 16 and Figure 17). The data was displayed for each compound in the following manner: name, chemical structure, optical rotation, melting point, UV, molecular and weight formulae, NMR, and reference(s) (Table S1). The principal goal of this work is to provide the researchers with detailed references that can assist them in the rapid identification of isolated depsides by comparing their physical and spectral data. Nevertheless, highlighting the bioactivities of these metabolites may attract the attention of synthetic and medicinal chemists to synthesize new agents, using known depsides as start materials. Literature searches for published studies were performed through diverse databases: Web of Science, PubMed (MedLine), GoogleScholar, Scopus, SciFinder, Springer-Link, Wiley, and ACS (American Chemical Society) Publications using keywords (depsides, isolation, fungi, biosynthesis, NMR, and biological activities).

## 2. Biosynthesis of Depsides

Depsides are acetyl-poly-malonyl-derived polyketides that are biosynthesized by polyketide synthase (PKS) [28,29,30,31,32,33,34]. PKS is composed of a minimal set of KS (ketosynthase), AT (acyltransferase), and ACP (acyl carrier protein) domains [30]. The non-reduced framework of the depside rings reveals that its corresponding PKS belongs to non-reducing PKSs (NR-PKSs). Depsides consist of two orsellinic acid molecules, connected by an ester linkage. Therefore, orsellinic acid can be considered the constructing unit of all depsides [28]. Biosynthetically, orsellinic acid is produced from a linear tetraketide chain. This chain is formed through an acetate-malonate pathway that is catalyzed by PKSs [29,30]. The tetraketide chain forming β-orsellinic acid (methyl-3-orsellinate) is produced by introducing a CH_3_ group obtained from SAM (S-adenosylmethionine) by the methyl transferase (CMeT) domain of the corresponding PKS [31]. Then, the non-enzymatic 2,7-aldol condensation of these chains produces orsellinic and β-orsellinic acids. Furthermore, the molecular skeleton is probably designed by post-biosynthetic tailoring enzymes, such as cyclases and hydrolases [18]. *p*-Depsides are produced by the condensation of either orsellinic acid and orcinol derivatives or by two methyl-3-orsellinate or orsellinate moieties, through the formation of an ester [12]. The consequent condensation of an additional unit produces a tri-depside, and two moieties yield a tetra-depside [32]. Moreover, depsides containing alkyl side-chains can be produced by the reduction of the terminal ketone groups, resulting in the required saturated alkyl moieties. *m*-Depsides are formed through the hydroxylation of the para-depside B-ring, subsequently followed by rearrangement [33] (Figure 2).

## 3. Biological Activities

Despite the unique structures of depsides, they have not been well investigated in terms of their pharmacological activities. The literature survey revealed that depsides have various biological activities (Table 2). Thus, an overview of their reported pharmacological activities is summarized in Table 2 and is described in detail below.

### 3.1. Antitumor Activity

Cancer is considered the second cause of death after cardiovascular diseases [34]. In 2020, around 10 million deaths were estimated to have been due to cancer, 70% of which were in middle- and low-income countries [35]. Most of the anticancer agents cannot distinguish between abnormal and normal cells; thus, researchers have been directed to develop selective and safe anticancer drugs that target the abnormal cancerous cells and have minimal effects on normal cells. Fungi represent an important source of anticancer agents, with significant benefits against various tumors [36]. It is noteworthy to mention that most of the reported depsides showed activity on cancer cells with no or little effect on normal cells.

Lünne et al. [37] evaluated the antitumor effect of lecanoric acid (**1**) and ethyl lecanorate (**2**) purified from *Claviceps purpurea* on HepG2 (human liver cancer cells) and CCF-STTG1 (human astrocytoma cells) using the CTC (5-cyano-2,3-bis(4-methylphenyl)-2H-tetrazolium chloride) assay. Both metabolites produced a dose-dependent antitumor effect on the tested cell lines. They reduced the CCF-STTG1 cell viability down to ~60%, at a concentration of 40 μM, and HepG2 cell viability by ~30% and 40%, respectively. Similar to HepG2 cells, **2** had the strongest antitumor effect on CCF cells (IC_50_ value of 54 μM) [37]. In the MTT ((3-(4,5-dimethylthiazol-2-yl))-2,5-diphenyl-2H-tetrazolium bromide) assay, aspergisides A (**3**), B (**4**), and C (**5**) showed weak antitumor activity, with IC_50_ values in the range of 45–114 µM toward Vero, MCF-7, and KB cell lines, compared with doxorubicin [38]. MS-3 (**22**) was inactive against Ehrlich ascites for leukemia and carcinoma in vivo; however, it was active toward Yoshida sarcoma cells (ID_5o_ value of 85 µg/mL) in vitro. Its activity was suggested to be due to a glyoxalase inhibition, as it possessed a glyoxalase inhibitory effect with an ID_50_ value of 12 µg/mL in the spectrophotometric assay [39]. In addition, **23** possessed significant antitumor activity toward A549 and HepG2, with IC_50_ values of 13.14 and 49.02 µM, respectively, compared to cisplatin (IC_50_ 14.33 and 18.74, respectively) in the MTT assay [40]. Compounds **27**–**29** were assayed against NCI-H187, Vero, BC, and KB cells, employing an MTT assay. Compounds **27** and **28** exhibited a significant antitumor effect against BC, with IC_50_ values of 8.8 and 4.4 µM, respectively, compared to ellipticine (IC_50_ 0.49 µM), while they showed weak to moderate effectiveness toward other cell lines, with IC_50_ values ranging from 13.0 to 34.3 µM [41]. Arenicolins A (**30**) and B (**31**), two new depsides having C-glycosyl moiety and dual heptyl side-chains, were isolated from *Penicillium arenicola* and assessed for antitumor activity at a concentration of 30.0 μM toward IMR-32, HCT-116, and BT-474 cell lines using an ICC (immunocytochemistry) assay. Compound **30** reduced cell viability with IC_50_ values of 6.0, 7.3, and 9.7 μM, respectively, compared to 5-FU (5-fluorouracil, IC_50_ 6.5 μM for HCT-116 and 5.7 μM for IMR-32). However, **31** did not have a significant antitumor effect toward the tested cell lines at a concentration of > 30 μM [42].

CRM646-A (**36**) and CRM646-B (**37**) were discovered from *Acremonium* sp. that showed a potent anti-metastatic capacity toward B16-F10 melanoma cells, with an IC_50_ value of 15 µM for **36** and IC_50_ 30 µM for **37** [43]. They also caused the dose-dependent inhibition of heparinase, with IC_50_ values of 3 and 10 µM, respectively, in comparison to suramin (IC_50_ value of 5 µM) [43,44]. Asami et al. established that CRM646-A (**36**) induced the inhibition of cells’ invasion, migration, and growth in tumor cells, due to its induction of nucleus condensation, plasma membrane disruption, and morphological changes in result to the increase in Ca^2+^ levels; thus, it could potentially be used as an effective anti-metastatic agent [45]. Compounds **45** and **46** in the MTT assay showed an antitumor effect against A549 and MAD-MB-435, with IC_50_ values of 16.82 and 37.01 μM, and 20.75 and 37.73 μM, respectively, compared with epirubicin (IC_50_ 0.26 and 5.60 μM, respectively); however, **11** did not exhibit obvious activity [46]. Togashi et al. reported that **36** and **49** prohibited telomerase activity at doses of 3.2 and 32 µM, respectively. In addition, they inhibited viral reverse transcriptase activity at almost the same dose levels; therefore, they may inhibit universal RNA-dependent DNA polymerases [47]. Compound **50**, a tridepside, was obtained from MSX 55526 fungus and showed moderate activity against the MCF-7, H460, and SF268 cell lines in the SRB assay, with IC_50_ values of 7.3, 6.6, and 8.1 µM, respectively, compared to camptothecin (IC_50_ 0.07, < 0.01, and 0.04 µM, respectively) [48].

### 3.2. Antimicrobial Activity

The wide use of antibiotics leads to the development of resistant microbes [85]. Moreover, the number of efficient drugs against life-threatening fungal and bacterial infections has decreased dramatically because of emerging pathogens that are multidrug-resistant (MDR), which is the biggest obstacle to success during the treatment of infectious diseases [86]. Therefore, there is a growing demand for new antimicrobial compounds. Fungi are considered an important source of novel antimicrobials because of their rich secondary metabolites and abundant species diversity. Bacterial enoyl-ACP (acyl carrier protein) reductase accelerates the last and rate-limiting step in type II FAS (bacterial fatty acid synthesis) [87,88]. Enoyl-ACP reductase includes three isoforms, FabK, FabI, and FabL. It is found in most bacteria: *S. aureus* (FabI), *Streptococcus pneumonia* (FabK), *P. aeruginosa* and *Enterococcus faecalis* (FabK and FabI), *B. subtilis* (FabI and FabL), and *Mycobacterium tuberculosis* (InhA, a FabI homolog) [61,89]. This enzyme has been established as a novel target for treatment against infections produced by MDR pathogens.

Phainuphong et al. purified three new depsides, aspergisides A–C (**3**–**5**) from *Aspergillus unguis*, and assessed their antimicrobial potential against MRSA (methicillin-resistant *S. aureus*), *S. aureus*, *C. albicans*, flucytosine-resistant *C. neoformans*, and *M. gypseum* [38]. Compound **3** had a weak antibacterial activity toward *S. aureus* and MRSA, with an MIC (minimal inhibitory concentrations) value of 8 µg/mL, while **4** and **5** were inactive, with MIC values of 32–200 µg/mL in the agar diffusion method [38]. *Setophoma* sp. associated with guava fruits produced compounds **6**–**8** and **66**–**69** [19]. They did not have growth inhibition activity toward *E. coli*. However, **66**–**69** demonstrated inhibition of *S. aureus* with MIC values of 100, 6.25, 50, and 25 µg/mL, respectively, in comparison to tetracycline (MIC 3.12 µg/mL) [19]. Compounds **6** and **7** were inactive. Moreover, all compounds did not exhibit quorum-sensing inhibitory activity. Studying the structural activity relationship revealed that the activity increased with the full methylation of the B-ring; however, the additional CH_3_ group at ring A, especially at C-2, resulted in a decrease in activity [19]. The agonodepsides A (**12**) and B (**13**) were isolated from the filamentous fungus, F7524 [50]. In the fluorometric InhA assay, **12** inhibited *M. tuberculosis* InhA with an IC_50_ value of 75 µM, while **13** was inactive at 100 µM, compared with triclosan (IC_50_ 3.0 µM) [50].

*Emericella unguis* yielded **14**, which had antibacterial activity against *S. aureus*, and was inactive against *Vibrio parahaemolyticus* in the agar diffusion assay [51]. *Stereum rameale* afforded **22**, which was found to show powerful antibacterial activity toward *B. subtilis*, *B. cereus*, and *S. aureus*, with IZDs (inhibition zone diameters) of 25, 25 and 28 mm, respectively, while it showed no antibacterial activity against *E. coli*, *Salmonella* sp., and *P. aeruginosa*. Its MBCs (minimal bactericidal concentrations) were 10, 50, and 100 μg/mL for *B. subtilis*, *B. cereus*, and *S. aureus*, respectively [57]. Compounds **23** and **24** showed inhibitory activity against both *S. aureus* and MRSA with MIC values of 25.0 µg/mL, and antibacterial potential toward *B. subtilis* (MICs 25.0 and 50.0 µg/mL, respectively), in comparison to vancomycin (MICs 1.0, 1.0, and 0.5 µg/mL, respectively) in the agar plate diffusion assay. They had a weak antibacterial potential against *P. aeruginosa* [40]. The new depsides, arenicolins A (**30**) and B (**31**) that were purified from *Penicillium Arenicola*, did not show any growth inhibition toward *S. aureus*, *C. albicans*, and *C. neoformans* at a concentration of 100 μg/mL, utilizing the agar diffusion assay [42]. Kwon et al. reported that the depside galactopyranoside derivative **32**, isolated from *Sporothrix* sp., strongly inhibited *S. pneumoniae* FabK and *S. aureus* FabI, with IC_50_ values of 9.2 and 3.2 µM, respectively [61]. It also exhibited antibacterial activity against MRSA CCARM 3506 and CCARM 3167, as well as *S. aureus* RN4220, with MIC values of 16–32 µg/mL and strong activity against *B. subtilis* (KCTC 1021) and *E. faecalis* (KCTC 5191), with MIC values of 8–16 µg/mL [61]. Another study by Kwon et al. revealed that **32** prohibited *M. tuberculosis* InhA with IC_50_ value of 9.6 µM [89]. Moreover, **32** (conc. of 128) had a weak anti-bacterial capacity against *M. tuberculosis*, with a growth inhibition of 17.9% in the microbroth dilution [89]. *Sporothrix* sp. yielded **34** and **35**. Compound **34** exhibited weak antimicrobial activity against *S. aureus*, *E. faecium*, and *B. subtilis* (MIC values of 12.5, 100, and 12.5 µg/mL, respectively) and showed no activity against *C. albicans*, *P. aeruginosa*, *E. coli*, *P. vulgaris*, *S. sonnei*, *S. typhi*, and *K. pneumoniae* at a concentration of 100 µg/mL. However, **35** had weak activity against *S. aureus* and *B. subtilis* and no activity against the others [63]. In 2017, Aqueveque et al. isolated **41** from *Stereum hirsutum*, which showed an antifungal capacity against *Botrytis cinerea*, one of the most harmful phytopathogenic fungi, causing the crop disease known as grey mold [58]. It showed a significant inhibition of *B. cinerea* mycelial growth at concentrations of 1000 and 2000 µg/mL, reaching 67% and 76%, respectively. At a concentration of 500 µg/mL, it produced 96% inhibition of sporulation. It showed an MIC of 10 µg/mL and MFC (minimal fungicidal concentration) of 50 µg/mL, compared with rovral (MFC of 10 µg/mL and MIC of 1 µg/mL) in the microdilution plate assay [58].

Compound **45**, a new tridepside produced by *Colletotrichum gloeosporioides* and associated with *Artemisia mongolica*, was tested in the disk diffusion assay for *B. subtilis*, *S. aureus*, *S. lutea*, *Pseudomonas* sp., *C. albicans*, *A. niger*, *C. elegans*, *H. sativum*, and *T. rubrum*. It exhibited antibacterial potential toward *B. subtilis* (MIC 25 µg/mL), *S. aureus* (MIC 50 µg/mL), and *S. lutea* (MIC 50 µg/mL) in comparison to ampicillin (MIC values of 0.05, 0.5, and 0.01 µg/mL, respectively) and an antifungal effect toward *H. sativum*, with an MIC value of 50 µg/mL, relative to triadimefon (MIC value of 20 µg/mL) [65].

*Phoma* sp. associated with *Kandelia candel* yielded the compounds **11**, **45**, and **46**. Compound **45** showed marked antimicrobial activity against *B. subtilis*, *P. aeruginosa*, MRSA, and *C. albicans*, with MIC values ranging from 3.27 to 6.55 μg/mL, compared with ampicillin (MIC values of 0.07, 0.15, and 0.15, respectively) and ketoconazole (MIC 0.10 μg/mL) in the disk diffusion assay. Moreover, it had weak activity toward *Salmonella typhimurium* with an MIC value of 26.20 μg/mL. Compound **46** was active against MRSA and *P. aeruginosa*, with MIC values of 3.36 and 1.67 μg/mL, respectively, and was weakly active toward *B. subtilis* (26.9 μg/mL), while **11** had antibacterial activity toward *B. subtilis* with an MIC value of 9.70 μg/mL [46]. The novel depside, PS-990 (**47**), produced by *Acremonium* sp., had antibacterial potential against *B. subtilis*, with an MIC value of 0.65 µg/mL and weak activity against *E. faecium* and *S. aureus* (MICs 21.0 and 10.0 µg/mL, respectively) [66]. Compound **49** prohibited the formation of peptidoglycan in the in vitro assay, with an IC_50_ value of 5 µg/mL in *E. faecalis* (strain A256) compared with a panel of antibiotics, suggesting that it interfered with cell wall trans-glycosylation [90].

The endophytic fungus *Cladosporium uredinicola*, isolated from *Psidium guajava* fruits, produced **9**, **10**, **80**, and **81**. The metabolites **9**, **80**, and **81** were assessed against *S. aureus*, *E. coli*, *P. aeruginosa*, and *B. subtilis*. Compound **9** inhibited the growth of *P. aeruginosa* and *B. subtilis*, with MIC values of 25 µg/mL, and *E. coli* and *S. aureus*, with MIC values of 250 µg/mL. On the other hand, **80** displayed a bacteriostatic effect (dose of 250 µg/mL) toward all tested bacteria, while **81** had a bacteriostatic effect for *S. aureus* and *E. coli* at a dose of 250 µg/mL and at a dose of 25 µg/mL for *P. aeruginosa* and *B. subtilis* [49]. Moreover, **86** and **88**–**90** had antibacterial activity against *B. subtilis*, with IZDs ranging from 7 to 11 mm; however, they were inactive against *Saccharomyces sake*, *P. aeruginosa*, *E. coli*, *Mycobacterium smegmatis*, *Micrococcus luteus*, *Pyricularia oryzae*, *S. aureus*, *C. albicans*, *Mucor racemosus*, and *A. niger* with the paper-disk method [74]. In the disk diffusion method, compound **91**, biosynthesized by *Humicola* sp., possessed no antimicrobial capacity against *B. subtilis*, *M. smegmatis*, *S. sake*, *M. luteus*, *P. aeruginosa*, *E. coli*, *M. racemosus*, *S. aureus*, *A. niger*, *P. oryzae*, and *C*. *albicans* [76].

### 3.3. Antifouling Activity

The anti-larval settlement activities of **15**, **48**, **56**, **58**, **59**, and **70**–**79** were assessed towards cyprid larvae of *B. amphitrite* [52]. Compounds **15**, **48**, **56**, **71**–**73**, and **76**–**78** deterred larval settlement, with EC_50_s ranging from 2.95 to 69.19 µM in comparison to butenolide (EC_50_ 4.62 µM). At a concentration of 10 µM, **15, 71**–**73**, and **77** exhibited narcotic potential toward *B. amphitrite* cyprids. They caused the loss of the phototactic response of cyprids, in addition to decreasing the appendage activity and cyprids becoming completely immobilized. The recovery rates of cyprids treated with **15**, **71**–**73**, and **77** (concentration of 10 µM) revealed that larvae possessed the highest recovery rate after treatment with **71**, while no larvae recovered after treatment with **15** for 24 h. From all tested compounds, **71** had an excellent antifouling potential and cyprids treated with it had the highest recovery rate. Thus, **71**, **72**, and **77** were reversible inhibitors. Conversely, **58**–**59**, **74**, and **75** had no effect [52].

### 3.4. Anti-Diabetic Activity

Diabetes is among the most prevalent chronic diseases and is characterized by hyperglycemia, which leads to damage of the blood vessels. This may produce macro- and micro-vascular disorders, as well as other complications, such as sexual dysfunction, dementia, lower-limb amputations, and depression [91]. Diabetes prevalence is expected to be at approximately 366 million cases by the year 2030 [92]. The side effects of the available hypoglycemic agents necessitate the discovery of efficient, low-side-effect, and affordable agents for treating diabetes.

Rivera-Chávez et al. reported that the tridepside, **59** (dose 3.1–31.6 mg/kg), reduced glucose blood levels after 30 min of oral administration of the sucrose load in mice (3.0 g/kg); however, only the highest dose (31.6 mg/kg) caused a marked reduction in blood glucose levels in NA-STZ (nicotinamide-streptozotocin) diabetic mice, indicating that **59** (doses of 3.1 and 10 mg/kg) reduced the blood glucose levels in both diabetic and normal mice [67].

### 3.5. D-Glucose-6-Phosphate Phosphohydrolase Inhibitory Activity

G6Pase (D-glucose-6-phosphate phosphohydrolase) is a hepatic metabolism-regulating enzyme, that catalyzes the last steps of glycogenolysis and gluconeogenesis pathways [93]. Its inhibition decreases the output of hepatic glucose from both pathways, leading to lowering the blood glucose levels in diabetes. The tetra-depside, **97** isolated from *Chloridium* sp. CL48903 prohibited G6Pase in rat liver microsomes (IC_50_ 1.6 µM) at a concentration of 133 µM, using a colorimetric assay and hepatocyte glucose output (81% inhibition), indicating the role of **97** as a G6Pase inhibitor [77].

### 3.6. α-Glucosidase Inhibitory (αGI) Activity

The α-glucosidase enzyme is an important therapeutic target for treating carbohydrate-mediated diseases. It catalyzes the breakdown of oligo- and disaccharides into monosaccharides in the final stage of carbohydrate digestion, leading to a rise in glucose levels [94,95,96,97]. Several studies revealed that α-glucosidase inhibitors (αGIs) slow down the digestion and absorption of carbohydrates, and thus reduce the postprandial blood glucose level [94,95,96,97]. The serious side effects of the current αGIs, such as liver injuries and gastrointestinal damage, have directed research efforts toward discovering and developing new and safer anti-diabetic agents.

*Stereum hirsutum* produced isoprenylated depsides; **17**–**23**, **38**–**40**, and **42**–**44**, which possessed an αGI capacity with IC_50_ values ranging from 3.06 to 36.64 µM, in comparison to acarbose (IC_50_ 640.88 µM). Compounds **21** and **42** had no αGI activity (IC_50_ > 50 µM). Compounds **17**–**19** displayed stronger αGI activities than **20**–**23**, revealing that ring-B substitution with carbonyl functionality can increase activity. Furthermore, **17** showed much stronger activity than **16**, confirming that the isoprenyl group strongly influences the activity. However, furan ring formation at C-2 and C-3 in **16**, and C-8 connectivity of a propane-1,2,3-triol moiety in **21**, greatly reduced activity levels [53]. Compounds **11**, **45**, and **46** exhibited significant αGI activity, with IC_50_ values of 60.20, 36.2, and 35.80 μM, respectively, compared to 1-deoxynojirimycin (62.8 μM), in the colorimetric α-glucosidase assay [46]. The tridepsides, **48**, **58**, and **59**, exhibited higher *Saccharomyces cerevisieae* α-glucosidase (αGHY) inhibitory activity, with IC_50_ values of 23.8, 15.8, and 22.1 µM, respectively, than acarbose (IC_50_ 545 µM). They are considered non-competitive inhibitors with Ki values of 27.8–66.2 µM. On the other hand, **58** prohibited the activity of αGHBs (α-glucosidase from *Bacillus stearothermophilus*), with an IC_50_ of 30.5 µM, which was less active than acarbose (IC_50_ 0.015 µM) [67].

### 3.7. Protein Tyrosine Phosphatase Inhibitory (PTP1BI) Activity

PTP1B (protein-tyrosine phosphatase 1B) is a negative regulator of the insulin signaling pathway. The inhibition of PTP1B activity has great promise for alleviating insulin and leptin resistance; hence, PTP-1BIs (PTP1B inhibitors) show potential for treating T2DM and other metabolic disorders [98].

*Cosmospora* sp. produced aquastatin A (**32**) (IC_50_ 0.19 µM) that showed modest but selective PTP1BI activity over other PTPs (protein tyrosine phosphatases) such as TCPTP (T-cell protein tyrosine phosphatase) (IC_50_ 0.51 µM), SHP-2 (IC_50_ > 44 µM), CD45 (IC_50_ > 44 µM), and LAR (IC_50_ > 44 µM), compared with ursolic acid (IC_50_ 2.5 µM). It was suggested that the 2,4-dihydroxy-6-pentadecylbenzoic acid moiety is critical for PTP1BI activity [60].

### 3.8. Diacylglycerol Acyltransferase Inhibitory (DGATI) Activity

Postprandial hypertriglyceridemia is considered the main risk factor for cardiovascular functions. Thus, triglyceride synthesis inhibition has remarkable therapeutic potential in metabolic disorder treatment. The enzymes known as diacylglycerol acyltransferases (DGATs) catalyze the final and only committed step in the biosynthesis of triglycerides [99]. Therefore, these enzymes could be a potential therapeutic target to combat cardio-metabolic disorders [71,82,99]. Compound **9** also inhibited TG synthesis (IC_50_ 91 µM), as well as PC and PE syntheses, indicating that it had a non-specific DGATI effect [76]. The compounds **86** and **88**–**90** were purified from *Humicola* sp. by Tomoda et al. [74]. Compound **88** was the most potent DGATI, with an IC_50_ of 10.2 µM, followed by **86** (IC_50_ 17.5 µM), **89** (IC_50_ 19.2 µM), and **90** (IC_50_ 51.6 µM). They also inhibited the formation of triacylglycerol using Raji cells on the intact cell assay, with IC_50_ values ranging from 2.82 to 17.2 µM. At high concentrations, **86** moderately inhibited the formation of phosphatidylethanolamine (PE) and phosphatidylcholine (PC), whereas **89** possessed a weak effect, indicating that **89** specifically suppressed the formation of triacylglycerol (TG) [74]. Moreover, **91**, when isolated from *Humicola* sp. FO-5969, showed a dose-dependent inhibition of DGAT using rat liver microsomes in the enzyme assay. At an IC_50_ 124 µM, it was weaker than **88** (IC_50_ 10.2 µM), revealing the fact that the 11-OH group was important for potent DGAT-I. Inokoshi et al. purified from *Humicola* sp. six new tridepsides, **87** and **92**–**96**, as well as the known metabolites, **86** and **88**–**90** [71]. In the enzyme assay, utilizing microsomal fractions prepared from *S. cerevisiae* expressing human DGAT-2 and DGAT-1, the non-glycoside compound **96** inhibited DGAT-1 and DGAT-2 with an IC_50_ value of 40 µM, whereas the glycosylated metabolites **92**–**95** had no and/or very weak DGATI potential, suggesting that the sugar moiety at C-11 reduced the DGATI. However, the compounds **86** and **88**–**90** were dual DGATIs, with IC_50_ values ranging from 20 to 170 µM for DGAT-1 and 30–170 µM for DGAT-2 [71,82].

### 3.9. Activity of 11β-Hydroxysteroid Dehydrogenase Inhibitory (11β-HSDI) Enzyme

High levels of glucocorticoid produce insulin resistance and glucose intolerance, leading to metabolic syndrome (MS) [100]. The enzyme 11β-HSD (11β-hydroxysteroid dehydrogenase) is accountable for the production of glucocorticoids in tissues, thus it plays a remarkable role in T2DM and MS. The 11β-HSD1 inhibitors (11β-HSD1Is) could be considered promising therapeutics in treating MS. Compounds **38**–**41** exhibited powerful and selective inhibitory activities against 11β-HSD1 in the HTRF immunoassay. They inhibited human 11β-HSD1 activity in a dose-dependent manner with IC_50_ values ranging from 240 to 6600 nM. Compounds **38** and **40** were the most active with IC_50_s 240 and 230 nM, respectively, while they did not prohibit 11β-HSD2 (IC_50_ > 10,000 nM) [64].

### 3.10. Anti-Inflammatory Activities

Inflammation is a beneficial and complicated immune system response to tissue damage or external challenges [101]. Prolonged uncontrolled inflammation leads to various diseases, such as cancer, diabetes, and neurodegenerative and cardiovascular disorders, due to the expression of various inflammatory mediators [102]. The anti-inflammatory potential was estimated by assessing the suppression of pro-inflammatory cytokine expression (e.g., IL-6, TNF-*α*, and IL-1β), pro-inflammatory enzymes (e.g., iNOS, COX-2), derived production (PGE_2_ and NO), and various inflammatory signal pathways in immune monocytes and macrophages (e.g., RAW264.7 cells, BV2 cells), whether in vitro, stimulated by LPS (lipopolysaccharide) [103], or by the inhibited swelling rate in a mouse ear edema model in vivo [104].

Compound **23**, biosynthesized by *Stereum hirsutum*, exhibited noticeable NO inhibitory potential (IC_50_ 19.17 µM) in the LPS-induced macrophages, compared with hydrocortisone (IC_50_ 48.15 µM) [40]. Moreover, **48** (ID_50_ 12 µM) and **49** (ID_50_ 9 µM) possessed considerable anti-inflammatory potential for the conversion of ^14^C-arachidonic acid into PGF_2_α plus PGE_2_ by the microsomes of ram seminal vesicles [69,81]. ID_50_s of the conversion of arachidonic acid (AA) into PGH_2_ (prostaglandin H_2_), PGH_2_ into (prostaglandin E_2_), and thromboxane A_2_ (TXA_2_) synthetase are 10, 40, 150 µM, respectively, for **48** in comparison to indomethacin (ID_50_ 30 for PGH_2_ and 130 µM for PGE_2_) and imidazole (ID_50_ 200 µM for TXA_2_ synthetase); meanwhile, **49** had ID_50_ values of 40, 9, and 350 µM, respectively. Compound **48** had a strong inhibitory effect on the conversion of AA into PGH_2_, while **49** specifically inhibited the step involving PGE_2_ synthesis from PGH_2_. Moreover, they inhibited TXA_2_ synthesis in bovine platelet microsomes (ID_50_ values of 150 and 350 µM, respectively), which was comparable to imidazole (200 µM) [69,81]. Both compounds (dose 50 mg/kg, orally) showed no significant anti-inflammatory effects on carrageenan-induced edema in rats. However, **49** caused a 70% inhibition of this edema system at an intravenous (IV) dose of 5 mg/kg, while **48** displayed no activity, even with IV administration [81]. Matsumoto et al. also stated that **8**, **49**, and **51**–**53** had powerful rat PLA_2_-II inhibitory potential (IC_50_ values ranged from 0.45 to 43 µM) in comparison to manoalide (IC_50_ 2.0 µM) [70], while they had marked capacities toward human PLA2 (phospholipase A2)-II (IC_50_s 29, 2.4, 2.1, 6.2, and 9.3 µM, respectively) relative to manoalide (IC_50_ 1.5 µM) [70].

### 3.11. Antimalarial Activity

Malaria is among many prevalent health concerns and is caused by the *Plasmodium* parasite in several of the world’s tropical regions [105]. The emergence of malaria strains that are drug-resistant to the available therapeutics makes the discovery of new antimalarial agents a great scientific challenge [14,106].

The two new depside galactopyranosides, **28** and **29**, and their aglycone **27**, isolated from *Acremonium* sp., were tested against *Plasmodium falciparum* K1 using a microculture radioisotope technique. Only compound **27** was active towards *P. falciparum* K1, with an IC_50_ value of 9.9 µM compared with dihydroartemisinin (IC_50_ 0.0039 µM). However, **28** and **29** had weak effects (IC_50_ > 10 µM) [41].

### 3.12. Antioxidant Activity

The depsides, **23** and **24** showed weak radicals scavenging capacity with EC_50_ > 200 µM [40]. In the DPPH (2-diphenyl-1-picrylhydrazyl) assay, **11**, **45**, and **46** also had weak antioxidant activity, compared with ascorbic acid [46].

### 3.13. Ca^2+^/CaM Dependent Phosphodiesterase Inhibitory (CaM-PDEI) Activity

Calmodulin (CaM) is a prevalent Ca^2+^-binding protein that regulates several Ca^2+^-dependent cellular functions in physiological and pathophysiological processes [107]. It is implicated in the cytoskeleton function and architecture, cell motility, apoptosis, cell proliferation, autophagy, the dephosphorylation/phosphorylation of proteins, reproductive processes, ion channel function, the relaxation/contraction of smooth muscle, and gene expression [108]. CaM can regulate these processes via modulating various proteins, including enzymes: phosphodiesterase, kinases, NOS (nitric oxide synthases), phosphatases, and ion channels. CaM-PDE is a key enzyme that is embroiled in the complex interactions between the cyclic nucleotide and Ca^2+^-second messenger systems [108]. Moreover, CaM is linked with several pathological states, including smooth muscle malfunctions and unregulated cell growth. CaM-PDEIs may play an important role in treating various disorders, such as neurodegenerative diseases and cancer [109].

Nakanishi et al. reported that **34** and **35**, purified from *Sporothrix* sp., inhibited heart and bovine brain PDEs (IC_50_ 4.3 and 1.8 µM and 5.9 and 15.0 µM, respectively) [63]. Moreover, they prohibited the CaM-dependent activities of CaM-PDEs but had a low effect against their CaM-independent effects, suggesting that these compounds interacted with CaM to inhibit Ca^2+^/CaM-dependent enzymes. On the other hand, they had no inhibitory activities on protein kinase C [63]. Moreover, PS-990 (**47**), isolated from *Acremonium* sp., inhibited brain CaM-PDE with an IC_50_ value of 3 µg/mL and did not elevate the intracellular cyclic AMP level. It markedly induced the neurite extension of Neuro2A (mouse neuroblastoma) at concentrations ranging from 10 to 30 µg/mL, suggesting its neuritogenic effect. It inhibited both cell growth and thymidine incorporation into the cells at the same concentration range. Interestingly, **47** reversibly induced neurite formation, with cell growth arrest through a mechanism other than increasing the intracellular cyclic AMP concentration [66,110].

### 3.14. Antiviral Activity

HCMV (human cytomegalovirus) is the most familiar viral cause of congenital infections, which can lead to severe birth defects. Its current treatments include viral DNA polymerase inhibitors, which block the late stages of HCMV replication; however, they do not prohibit the viral induction of multiple cell activation events [111]. Thus, it may be beneficial to discover new treatments for HCMV infections.

Compounds **27**–**29** were assessed against HSV-1 (*Herpes simplex* virus type 1), using the SBR technique. Only **28** showed potent activity, with an IC_50_ value of 7.2 µM, compared with acyclovir (IC_50_ 10.2 µM), while **27** and **29** displayed weak activity, with IC_50_ values of > 1000 and > 50 µM, respectively [41]. *Cytonaema* sp. yielded novel *p*-tridepsides; **84** and **85** showed in vitro inhibitory activities to hCMV protease, with IC_50_ values of 43 and 11 µM, respectively, in the scintillation proximity assay [73].

### 3.15. Human Leukocyte Elastase (HLE) Inhibitory Activity

HLE is one of the most destructive enzymes that can degrade tissue matrix proteins, such as collagen, elastin, fibronectin, proteoglycan, and laminin, by activating progelatinase, procollagenase, and prostromelysin [25]. It is released from PMNLs (polymorphonuclear leukocytes) as a result of inflammatory mediators and stimuli. HLE is considered an important therapeutic target for treating many inflammation-linked disorders [112].

The depsides, **25** (IC_50_ 45.1 µM) and **26** (IC_50_ 92.6 µM), weakly inhibited HLE in the spectro-photometric immunoassay, while the tridepside, **83** (IC_50_ 1.8 µM), exhibited high HLE inhibitory activity compared to ulinastatin (IC_50_ 1.1 µg/mL), which was 25–50-fold greater than that of depsides [25].

### 3.16. Indoleamine 2,3-Dioxygenase Inhibitory (IDOI) Activity

IDO (indoleamine 2,3-dioxygenase) catalyzes the tryptophan catabolism initial step via the KP (kynurenine pathway) [68]. Dysregulation of the KP is accompanied by the IDO activity elevation and production of quinolinic acid (an excitotoxin), which has been engaged in the pathogenesis of neurodegenerative disorders, neuroinflammatory, HIV encephalitis, age-related cataract, and depression [68]. Therefore, IDO is a promising target of new therapeutics for treating neurological disorders and cancer, as well as other disorders characterized by a defect in tryptophan metabolism.

Compounds **49**, **54**, and **65** isolated from *Coniochaeta* sp., inhibited the activity of IDO with IC_50_ values of 21.2, 14.5, and, 26 µM, respectively in comparison to menadione (IC_50_ 3.7 µM) [68].

### 3.17. Adenosine Triphosphatase Inhibitory Activity

Na^+^/K^+^-ATPase (sodium/potassium adenosine triphosphatase) is an integral membrane protein that is accountable for maintaining Na^+^ and K^+^ gradients across the plasma membrane, an important process for mammalian cell survival. Currently, it is extensively studied as a potential target for cancer treatment, especially in glioblastoma and lung cancer [113]. The proton pump, H^+^/K^+^ ATPase, plays an important role in the stomach acidification process. Its inhibition in gastric parietal cells decreases gastric acid overproduction [114]. H^+^/K^+^ ATPase inhibitors can be utilized as a target for developing drugs against gastric acid production disturbances.

Aquastatin A (**32**) was biosynthesized by *Fusarium aquaeductuum*. It inhibited Na^+^/K^+^-ATPase (adenosine triphosphatase) (IC_50_ 7.1 µM) and H^+^/K^+^-ATPase (IC_50_ 6.2 µM) [59].

### 3.18. Proteasome Inhibitory Activity

Proteasome comprises one or two 19S RPs (regulatory particles) and 20S CPs (core particles). In humans, the 20S CP formation is assisted by proteasome-specific chaperones: PAC1–PAC4 and POMP (proteasome maturation protein) [115,116]. Proteasome accounts for misfolded, unneeded, or damaged cellular protein degradation. Therefore, it is a crucial target for the future treatment of various diseases, such as neurodegenerative and autoimmune diseases, cystic fibrosis, cancer, diabetes, and atherosclerosis [115]. The compound **105** (IC_50_ 0.020 µM) had a potent PAC3 (proteasome-assembling chaperone 3 homodimer) inhibitory effect, while **103** and **107** (IC_50_ > 250 µM) did not inhibit the PAC3 homodimer [116].

### 3.19. Phospholipase Inhibitory Activities

Phospholipase A2 (PLA2) catalyzes the hydrolysis of membrane phospholipids into arachidonic acid; therefore, its inhibitors have the potential for treating various inflammatory disorders [117]. Compound **107** showed a strong reversible and noncompetitive inhibition of human PLA_2_-II (*Ki* value of 0.098 μM, IC_50_ value of 0.076 μM); however, it showed weak inhibition of human PLA_2_-I (IC_50_ of 18 μM). Its inhibitory effect toward PLA_2_-II human and PLA_2_ *Naja mocambique* was noticeably reduced by methylation of the two COOH groups. Furthermore, **107**, upon co-injection with carrageenan, remarkably reduced PLA_2_ activity and exudate volume in the carrageenan-induced pleurisy rat model [80]. Rat PLA_2_-II was the most sensitive to **99**, with an IC_50_ of 0.0033 µM and *K*i of 0.0068 µM. Furthermore, it showed 50% quenching of the PLA_2_ of *Naja naja* venom [78,83]. The metabolites, **48**–**53**, **98**–**101**, and **104**–**107**, purified from *T. terricola* RF-143, were tested for PLA_2_ inhibition [70,78]. Compound **99** (IC_50_ 0.0033 μM) was the most potent inhibitor against rat PLA_2_-II, and the other compounds had inhibitory effects, with an IC_50_ of 0.0078–0.070 μM. Compounds **48**–**53** showed strong inhibition toward human PLA2 (phospholipase A2)-II and rat PLA_2_-II, with IC_50_s of 2.1–29 µM and 0.45–43 µM, respectively, compared to manoalide (IC_50_s 1.5 µM and 2.0 µM, respectively) [70]. Moreover, **99** inhibited the histamine release from PLA2-stimulated mast cells. In addition, **99** (IC_50_ 1.4 μM) inhibited bee venom PLA_2_. The co-injection of **99** (1 μg/paw) with bee venom PLA_2_ reduced edema formation by 44.7%, via the inhibition of PLA2 activity [84]. It was reported that **99** repressed various secretagogues for stimulated degranulation in rat and mouse mast cells, without affecting PGD_2_ (prostaglandin D2) synthesis. Thus, the secretory PLA_2_ inhibition by **99** attenuated the severity of inflammation via repression of the degranulation process [118,119].

## 4. Conclusions

Recently, more focus has been given to fungi as they are excellent platforms for the biosynthesis of a huge number of structurally diverse metabolites. The knowledge of these metabolites offers a virtually untapped source of new bioactive metabolites with potential agrochemical and pharmaceutical uses. Fungi use these metabolites for defense, and many of these metabolites demonstrate a broad range of bioactivities. Among these metabolites are depsides that possess remarkable bioactivities. According to the listed results, there are 110 depsides that have been isolated from fungi. Most of them are reported from *Thielavia* (26.6%), *Stereum* (17.4%), *Chaetomium* (15%), and *Humicola* (12%) species (Figure 15).

*Thielavia*, *Chaetomium*, and *Humicola* belong to the Chaetomiaceae family; therefore, this family could be considered as one of the major producers of depsides. It is obvious that the largest number of depsides was isolated in 2002 (18 depsides), 2014 (17 depsides), 2017 (16 depsides), and 1995 (14 depsides) (Figure 16).

Most of the reported depsides have been evaluated for their α-GI (α-glucosidase inhibitory), antimicrobial, antitumor, antifouling, PLA2 (phospholipase A2), and DGATI (diacylglycerol acyltransferase inhibitory) abilities (Figure 17). Thus, these studies revealed that fungal depsides are a rich source for the discovery of effective and novel pharmaceutical leads and should be further exploited.

They also demonstrate inhibitory activities against various enzymes that can be utilized as targets for the treatment of various diseases. These metabolites could have potential as lead compounds for treating metabolic syndrome, obesity, and diabetes via the inhibition of various enzymes, such as HSD, PTP1BI, α-GI, G6Pase, and DGAT. However, extensive explorations of their mechanism of action, as well as structure modification, chemical synthesis, and structure/activity relationship analysis are needed. Despite the extensive structural diversity of depsides, none of them has been approved by the FDA, and none of them has as yet progressed to clinical trials. Therefore, the impact of fungal depsides on human health concerns has to be considered in several ways.

## Figures and Tables

**Table 1:** List of fungal depsides (Fungal source, host, and place).

Compound Name	Fungus	Host (Part)	Source, Place	Ref.
Lecanoric acid (**1**)	*Claviceps purpurea* (PKS7)	-	Culture	[37]
Ethyl lecanorate (**2**)	*Claviceps purpurea* (PKS7)	-	Culture	[37]
Aspergiside A (**3**)	*Aspergillus unguis* PSU-RSPG204 (BCC56860)	-	Soil, Surat Thani Province, Thailand	[38]
Aspergiside B (**4**)	*Aspergillus unguis* PSU-RSPG204 (BCC56860)	-	Soil, Surat Thani Province, Thailand	[38]
Aspergiside C (**5**)	*Aspergillus unguis* PSU-RSPG204 (BCC56860)	-	Soil, Surat Thani Province, Thailand	[38]
Lecanorin D (**6**)	*Setophoma* sp. (KM288713)	Fruits of *Psidium guajava*	Sӓo Carlos local trade, São Paulo state, Brazil	[19]
Lecanorin E (**7**)	*Setophoma* sp. (KM288713)	Fruits of *Psidium guajava*	Sӓo Carlos local trade, São Paulo state, Brazil	[19]
Lecanorin F (**8**)	*Setophoma* sp. (KM288713)	Fruits of *Psidium guajava*	Sӓo Carlos local trade, São Paulo state, Brazil	[19]
3-Hydroxy-2,5-dimethylphenyl 2,4-dihydroxy-3,6-dimethylbenzoate (**9**)	*Setophoma* sp. (KM288713)	Fruits of *Psidium guajava*	Sӓo Carlos local trade, São Paulo state, Brazil	[49]
3-Hydroxy-2,4,5-trimethylphenyl 2,4-dihydroxy-3,6-dimethylbenzoate (**10**)	*Setophoma* sp. (KM288713)	Fruits of *Psidium guajava*	Sӓo Carlos local trade, São Paulo state, Brazil	[49]
Colletotric acid C (**11**)	*Phoma* sp. (SYSU-SK-7)	Healthy branch of *Kandelia candel*	Shankou Mangrove Nature Reserve, Guangxi Province, China	[46]
Agonodepside A (**12**)	Fungal strain (F7524)	Leaves of *Derris thyrsiflora*	Singapore	[50]
Agonodepside B (**13**)	Fungal strain (F7524)	Leaves of *Derris thyrsiflora*	Singapore	[50]
Guisinol (**14**)	*Emericella unguis* (isolate 1 (M87-2)	*Stomolopus meleagris*	Paria Bay, Venezuela	[51]
	*Emericella unguis* (M90B-10)	Soft part of an unidentified mollusc	Paria Bay, Venezuela	[51]
Thielavin Z_7_ (**15**)	*Thielavia* sp (UST030930-004)	-	12-d Biofilms developed at the pier of the Hong Kong University of Science and Technology in Port Shelter, Hong Kong, China	[52]
Sterenin J (**16**)	*Stereum hirsutum* (EU851110)	-	Tibetan mountain	[53]
Sterenin E (**17**)	*Stereum hirsutum* (EU851110)	-	Tibetan mountain	[53]
Sterenin F (**18**)	*Stereum hirsutum* (EU851110)	-	Tibetan mountain	[53]
Sterenin G (**19**)	*Stereum hirsutum* (EU851110)	-	Tibetan mountain	[53]
Sterenin H (**20**)	*Stereum hirsutum* (EU851110)	-	Tibetan mountain	[53]
Sterenin I (**21**)	*Stereum hirsutum* (EU851110)	-	Tibetan mountain	[53]
MS-3 (**22**)	*Stereum hirsutum* (EU851110)	-	Tibetan mountain	[39,53,54,55,56]
	*Stereum rameale* (strain 2511)	The bark of a dead tree	Native forest of *Nothofagus* species (Nothofagaceae), near Ñuble National Reserve, Ñuble Province, Chile	[57,58]
4′-Hydroxy-5′-methoxy-6′-(3″-methyl-2″-butenyl)-phenyl-2,4-dihydroxy-6-methyl-benzoate/ 4-Hydroxy-3-methoxy-2-(3-methylbut-2-en-1-yl)phenyl 2,4-dihydroxy-6-methylbenzoate (**23**)	*Stereum hirsutum* (AB733150.1)		Tibetan Plateau, China	[40]
	*Stereum hirsutum* (EU851110)	-	Tibetan mountain	[53]
4′-Hydroxy-6′-(3″-methyl-2″-butenyl)-phenyl-2,4-dihydroxy-6-methyl-benzoate (**24**)	*Stereum hirsutum* (AB733150.1)	-	Tibetan Plateau, China	[40]
Nordivaricatic acid (**25**)	Fungal strain	-	Soil, Yunnan Province, China	[25]
65-Divarinyl divarate (**26**)	Fungal strain	-	Soil, Yunnan Province, China	[25]
KS-501a (3-Heptyl-5-hydroxyphenyl 2-heptyl-4,6-dihydroxybenzoate) (**27**)	*Acremonium* sp. (BCC 14080)	Palm leaf	Khao Yai National Park, Nakhon Ratchasima Province, Thailand	[41,56]
KS-501a-2-O-*β*-D-galactopyranose (**28**)	*Acremonium* sp. (BCC 14080)	Palm leaf	Khao Yai National Park, Nakhon Ratchasima Province, Thailand	[41]
KS-501a-2-O-*β*-D-digalactopyranose (**29**)	*Acremonium* sp. (BCC 14080)	Palm leaf	Khao Yai National Park, Nakhon Ratchasima Province, Thailand	[41]
Arenicolin A (**30**)	*Penicillium arenicola* (NRRL 8095)	**-**	Soil, British Columbia, Canada	[42]
	*Penicillium arenicola* (3392)	**-**	Soil, pine forest sample, near Kiev, Ukraine	[42]
	*Penicillium arenicola* (31507)	**-**	Mineral soil, under *Pinus resinosa*, Ontario, Canada	[42]
	*Penicillium arenicola* (31509)	**-**	Oil-soaked soil sample, Norman Wells, NW Territories, Canada	[42]
Arenicolin B (**31**)	*Penicillium arenicola* (NRRL 8095)	**-**	Soil, British Columbia, Canada	[42]
	*Penicillium arenicola* (3392)	**-**	Soil, pine forest sample, near Kiev, Ukraine	[42]
	*Penicillium arenicola* (31507)	**-**	Mineral soil, under *Pinus resinosa*, Ontario, Canada	[42]
	*Penicillium arenicola* (31509)	**-**	Oil-soaked soil sample, Norman Wells, NW Territories, Canada.	[42]
Aquastatin A (**32**)	*Fusarium aquaeductuum* (SANK 11089)	**-**	Slime fluxes, Karuizawa, Nagano Prefecture, Japan	[59]
	*Cosmospora* sp. SF-5060	**-**	Inter-tidal sediment, Gejae Island, Korea	[60]
	*Sporothrix* sp. (FN611)	**-**	Soil, Jeonju City, Jeollabuk-do, Korea	[61]
Depsitinuside (**33**)	Endophytic fungus, internal strain (8984)	Leaves of *Viburnum tinus*	-	[62]
KS-501 (2-(*β*-D-galactofuranosyloxy)-6-heptyl-4-hydroxybenzoic acid 3-heptyl-5-hydroxyphenyl ester) (**34**)	*Sporothrix* sp. (KAC-1985)	Fallen leaf	Yamakita-cho, Ashgarakami-gun, Kanagawa Prefecture, Japan	[56,63]
KS-502 (2-(*β*-D-galactofuranosyloxy)6-heptyl-4-hydroxybenzoic acid 4-carboxy-3-heptyl-5-hydroxyphenyl ester) (**35**)	*Sporothrix* sp. (KAC-1985)	Fallen leaf	Yamakita-cho, Ashgarakami-gun, Kanagawa Prefecture, Japan	[56,63]
CRM646-A (**36**)	*Acremonium* sp. (MT70646)	**-**	Soil, Geryong, Kongju, Korea	[43,44,47]
CRM646-B (**37**)	*Acremonium* sp. (MT70646)	**-**	Soil, Geryong, Kongju, Korea	[43,44,47]
Sterenin A (**38**)	*Stereum* sp. (SANK 21205)	**-**	Spore print of fresh basidiocarps, Gunma Prefecture, Japan	[64]
Sterenin B (**39**)	*Stereum* sp. (SANK 21205)	**-**	Spore print of fresh basidiocarps, Gunma Prefecture, Japan	[64]
Sterenin C (**40**)	*Stereum* sp. (SANK 21205)	**-**	Spore print of fresh basidiocarps, Gunma Prefecture, Japan	[64]
Sterenin D (**41**)	*Stereum* sp. (SANK 21205)	-	Spore print of fresh basidiocarps, Gunma Prefecture, Japan	[64]
Sterenin K (**42**)	*Stereum hirsutum* (EU851110)	-	Tibetan mountain	[53]
Sterenin L (**43**)	*Stereum hirsutum* (EU851110)	-	Tibetan mountain	[53]
Sterenin M (**44**)	*Stereum hirsutum* (EU851110)	-	Tibetan mountain	[53]
Colletotric acid A (**45**)	*Phoma* sp. (SYSU-SK-7)	Healthy branch of *Kandelia candel*	Shankou Mangrove Nature Reserve, Guangxi Province, China	[46]
	*Colletotrichum gloeosporioides*	Stem of *Artemisia mongolica*	Hillsides of the Zijin Mountain, suburb of Nanjing, China	[65]
Colletotric acid B (**46**)	*Phoma* sp. (SYSU-SK-7)	Healthy branch of *Kandelia candel*	Shankou Mangrove Nature Reserve, Guangxi Province, China	[46]
PS-990 (**47**)	*Acremonium* sp. (KY12702)	-	Soil, Tokyo, Japan	[66]
Thielavin A (**48**)	*Chaetomium carinthiacum* (ATCC 46463)	-	American Type Culture Collection	[62]
	*Thielavia* sp. (UST030930-004)	-	12-d Biofilms developed at the pier of the Hong Kong University of Science and Technology in Port Shelter, Hong Kong, China	[52]
	Endophytic fungus MEXU (27905)	Healthy leaves of *Hintonia latiflora*	México	[67]
Thielavin B (**49**)	*Coniochaeta* sp. (10F058a)	-	Soil, Korea	[68]
	*Chaetomium carinthiacum* (ATCC 46463)	-	American Type Culture Collection	[62]
	*Coniochaeta* sp. (10F058a)	-	Soil, Ochang, Korea.	[68]
Thielavin B methyl ester (**50**)	Mycosynthetix fungal strain 55526	-	Leaf litter, North Carolina Smoky Mountains, United States.	[48]
Thielavin C (**51**)	*Chaetomium carinthiacum* (ATCC 46463)	-	American Type Culture Collection	[62]
	*Thielavia terricola* (SANK 10475)	-		[69]
Thielavin D (**52**)	*Chaetomium carinthiacum* (ATCC 46463)	-	American Type Culture Collection	[62]
Thielavin E (**53**)	*Thielavia terricola* RF-143	-		[70]
Thielavin F (**54**)	*Chaetomium carinthiacum* (ATCC 46463)	-	American Type Culture Collection	[62]
	*Coniochaeta* sp. (10F058a)	-	Soil, Ochang, Korea	[68]
Thielavin G (**55**)	*Chaetomium carinthiacum* (ATCC 46463)	-	American Type Culture Collection	[62]
Thielavin H (**56**)	*Chaetomium carinthiacum* (ATCC 46463)	-	American Type Culture Collection	[62]
	*Thielavia* sp UST030930-004	-	12-d Biofilms developed at the pier of the Hong Kong University of Science and Technology in Port Shelter, Hong Kong, China	[52]
Thielavin I (**57**)	*Chaetomium carinthiacum* (ATCC 46463)	-	American Type Culture Collection	[62]
Thielavin J (**58**)	*Chaetomium carinthiacum* (ATCC 46463)	-	American Type Culture Collection	[62]
	*Thielavia* sp UST030930-004	-	12-d Biofilms developed at the pier of the Hong Kong University of Science and Technology in Port Shelter, Hong Kong, China	[52]
	Endophytic fungus MEXU 27905	Healthy leaves of *Hintonia latiflora*	México	[67]
Thielavin K (**59**)	*Chaetomium carinthiacum* (ATCC 46463)	-	American Type Culture Collection	[62]
	*Thielavia* sp UST030930-004	-	12-d Biofilms developed at the pier of the Hong Kong University of Science and Technology in Port Shelter, Hong Kong, China	[52]
	Endophytic fungus MEXU 27905	Healthy leaves of *Hintonia latiflora*	México	[67]
Thielavin L (**60**)	*Chaetomium carinthiacum* (ATCC 46463)	-	American Type Culture Collection	[62]
Thielavin M (**61**)	*Chaetomium carinthiacum* (ATCC 46463)	-	American Type Culture Collection	[62]
Thielavin N (**62**)	*Chaetomium carinthiacum* (ATCC 46463)	-	American Type Culture Collection	[62]
Thielavin O (**63**)	*Chaetomium carinthiacum* (ATCC 46463)	-	American Type Culture Collection	[62]
Thielavin P (**64**)	*Chaetomium carinthiacum* (ATCC 46463)	-	American Type Culture Collection	[62]
Thielavin Q (**65**)	*Coniochaeta* sp. (10F058a)	-	Soil, Ochang, Korea	[68]
Thielavin S (**66**)	The endophyte *Setophoma* sp. (KM288713)	Fruits of *Psidium guajava*	Sӓo Carlos local trade, São Paulo state, Brazil	[19]
Thielavin T (**67**)	The endophyte *Setophoma* sp. (KM288713)	Fruits of *Psidium guajava*	Sӓo Carlos local trade, São Paulo state, Brazil	[19]
Thielavin U (**68**)	The endophyte *Setophoma* sp. (KM288713)	Fruits of *Psidium guajava*	Sӓo Carlos local trade, São Paulo state, Brazil	[19]
Thielavin V (**69**)	The endophyte *Setophoma* sp. (KM288713)	Fruits of *Psidium guajava*	Sӓo Carlos local trade, São Paulo state, Brazil	[19]
Thielavin W (**70**	*Thielavia* sp UST030930-004	-	12-d Biofilms developed at the pier of the Hong Kong University of Science and Technology in Port Shelter, Hong Kong, China	[52]
Thielavin X (**71**)	*Thielavia* sp UST030930-004	-	12-d Biofilms developed at the pier of the Hong Kong University of Science and Technology in Port Shelter, Hong Kong, China	[52]
Thielavin Y (**72**)	*Thielavia* sp (UST030930-004)	-	12-d Biofilms developed at the pier of the Hong Kong University of Science and Technology in Port Shelter, Hong Kong, China	[52]
Thielavin Z (**73**)	*Thielavia* sp (UST030930-004)	-	12-d Biofilms developed at the pier of the Hong Kong University of Science and Technology in Port Shelter, Hong Kong, China	[52]
Thielavin Z_1_ (**74**)	*Thielavia* sp (UST030930-004)	-	12-d Biofilms developed at the pier of the Hong Kong University of Science and Technology in Port Shelter, Hong Kong, China	[52]
Thielavin Z_2_ (**75**)	*Thielavia* sp (UST030930-004)	-	12-d Biofilms developed at the pier of the Hong Kong University of Science and Technology in Port Shelter, Hong Kong, China	[52]
Thielavin Z_3_ (**76**)	*Thielavia* sp (UST030930-004)	-	12-d Biofilms developed at the pier of the Hong Kong University of Science and Technology in Port Shelter, Hong Kong, China	[52]
Thielavin Z_4_ (**77**)	*Thielavia* sp (UST030930-004)	-	12-d Biofilms developed at the pier of the Hong Kong University of Science and Technology in Port Shelter, Hong Kong, China	[52]
Thielavin Z_5_ (**78**)	*Thielavia* sp (UST030930-004)	-	12-d Biofilms developed at the pier of the Hong Kong University of Science and Technology in Port Shelter, Hong Kong, China	[52]
Thielavin Z_6_ (**79**)	*Thielavia* sp. (UST030930-004)	-	12-d Biofilms developed at the pier of the Hong Kong University of Science and Technology in Port Shelter, Hong Kong, China	[52]
Hydroxy-2,5-dimethylphenyl 4-[(2,4-dihydroxy-3,6-dimethylbenzoyl)oxy]-2-hydroxy-3,6-dimethylbenzoate (**80**)	*Cladosporium uredinicola*	Fruits of *Psidium guajava*	Sӓo Carlos local trade, São Paulo state, Brazil	[49]
Hydroxy-2,4,5-trimethylphenyl 4-[(2,4-dihydroxy-3,6-dimethylbenzoyl)oxy]-2-hydroxy-3,6-dimethylbenzoate (**81**)	*Cladosporium uredinicola*	Fruits of *Psidium guajava*	Sӓo Carlos local trade, São Paulo state, Brazil	[49]
Gyrophoric acid (82)	*Humicola* sp. FO-2942	-	Soil, Nagasaki, Japan	[71,72]
Trivaric acid (**83**)	Fungal strain	-	Soil, Yunnan Province, China	[25]
Cytonic acid A (**84**)	*Cytonaema* sp. (F32027)	*Quercus* sp.	United Kingdom	[73]
Cytonic acid B (**85**)	*Cytonaema* sp. (F32027)	*Quercus* sp.	United Kingdom	[73]
Amidepsine D (**86**)	*Humicola* sp. (FO-2942)	-	Soil, Nagasaki, Japan	[74,75]
Amidepsine K (**87**)	*Humicola* sp. (FO-2942)	-	Soil, Nagasaki, Japan	[71]
Amidepsine A (**88**)	*Humicola* sp. (FO-2942)	-	Soil, Nagasaki, Japan	[74,75]
Amidepsine B (**89**)	*Humicola* sp. (FO-2942)	-	Soil, Nagasaki, Japan	[74,75]
Amidepsine C (**90**)	*Humicola* sp. (FO-2942)	-	Soil, Nagasaki, Japan	[74,75]
Amidepsine E (**91**)	*Humicola* sp. (FO-5969)	-	Soil, Asaka, Saitama, Japan	[76]
Amidepsine F (**92**)	*Humicola* sp. (FO-2942)	-	Soil, Nagasaki, Japan	[71]
Amidepsine G (**93**)	*Humicola* sp. (FO-2942)	-	Soil, Nagasaki, Japan	[71]
Amidepsine H (**94**)	*Humicola* sp. (FO-2942)	-	Soil, Nagasaki, Japan	[71]
Amidepsine I (**95**)	*Humicola* sp. (FO-2942)	-	Soil, Nagasaki, Japan	[71]
Amidepsine J (**96**)	*Humicola* sp. (FO-2942)	-	Soil, Nagasaki, Japan	[71]
44-CJ-21,164 (**97**)	*Chloridium* sp. (CL48903).	-	China	[77]
Thielocin A1α (98)	*Thielavia terricola* (RF-143)	-	Culture	[78]
Thielocin A1β (99)	*Thielavia terricola* (RF-143)	-	Culture	[78]
Thielocin A2α (100)	*Thielavia terricola* (RF-143)	-	Culture	[70]
Thielocin A2β (101)	*Thielavia terricola* (RF-143)	-	Culture	[70]
Thielocin A4α (102)	*Thielavia terricola* (RF-143)	-	Culture	[79]
Thielocin A4β (103)	*Thielavia terricola* (RF-143)	-	Culture	[79]
Thielocin A3 (104)	*Thielavia terricola* (RF-143)	-	Culture	[70]
Thielocin B1 (105)	*Thielavia terricola* (RF-143)	-	Culture	[70]
Thielocin B2 (106)	*Thielavia terricola* (RF-143)	-	Culture	[70]
Thielocin B3 (107)	*Thielavia terricola* (RF-143)	-	Culture	[80]
Thielocin B3 monomethyl ester B (108)	*Thielavia terricola* (RF-143)	-	Culture	[80]
Thielocin B3 monomethyl ester C (109)	*Thielavia terricola* (RF-143)	-	Culture	[80]
Thielocin B3 monomethyl ester D (110)	*Thielavia terricola* (RF-143)	-	Culture	[80]

**Table 2:** Biological activities of fungal depsides.

**Compound Name**	**Activity**	**Assay/Microorganism/Model/Enzyme**	**Results**	**Positive Control**	**Ref.**
Lecanoric acid (**1**)	Antitumor	Colorimetric CTC/HepG2	40.0 µM (IC_50_)	T-2 toxin 10.0 µM (IC_50_)	[37]
Ethyl lecanorate (**2**)	Antitumor	Colorimetric CTC/HepG2	40.0 µM (IC_50_)	T-2 toxin 10.0 µM (IC_50_)	[37]
	Antitumor	Colorimetric CTC/CCF-STTG1	54.0 µM (IC_50_)	T-2 toxin 10.0 µM (IC_50_)	[37]
Aspergiside A (**3**)	Antibacterial	Agar diffusion assay/*S. aureus* ATCC25923	8 µg/mL (MIC)	Vancomycin 0.25 µg/mL (MIC)	[38]
	Antibacterial	Agar diffusion assay/MRSA	8 µg/mL (MIC)	Vancomycin 0.50 µg/mL (MIC)	[38]
	Antifungal	Agar diffusion assay/*C. albicans* NCPF3153	200 µg/mL (MIC)	Amphotericin B 0.25 µg/mL (MIC)	[38]
	Antibacterial	Agar diffusion assay/*C. neoformans* ATCC 9013	64 µg/mL (MIC)	Amphotericin B 0.25 µg/mL (MIC)	[38]
	Antifungal	Agar diffusion assay/*M. gypseum* SH-MU-4	128 µg/mL (MIC)	Miconazole 1.0 µg/mL (MIC)	[38]
	Antitumor	MTT/KB	53.69 µM (IC_50_)	Ellipticine 10.56 µM (IC_50_) Doxorubicin 0.65 µM (IC_50_)	[38]
	Antitumor	MTT/MCF-7	54.42 µM (IC_50_)	Doxorubicin 16.83 µM (IC_50_) Tamoxifen 20.78 µM (IC_50_)	[38]
	Antitumor	MTT/Vero	83.75 µM (IC_50_)	Ellipticine 4.55 µM (IC_50_)	[38]
Aspergiside B (**4**)	Antibacterial	Agar diffusion assay/*S. aureus* ATCC25923	128 µg/mL (MIC)	Vancomycin 0.25 µg/mL (MIC)	[38]
	Antibacterial	Agar diffusion assay/MRSA	128 µg/mL (MIC)	Vancomycin 0.50 µg/mL (MIC)	[38]
	Antibacterial	Agar diffusion assay/*C. neoformans* ATCC 9013	32 µg/mL (MIC)	Amphotericin B 0.25 µg/mL (MIC)	[38]
	Antitumor	MTT/Vero cells	114.40 µM (IC_50_)	Ellipticine 4.55 µM (IC_50_)	[38]
Aspergiside C (**5**)	Antibacterial	Agar diffusion assay/*S. aureus* ATCC25923	200 µg/mL (MIC)	Vancomycin 0.25 µg/mL (MIC)	[38]
	Antibacterial	Agar diffusion assay/MRSA	200 µg/mL (MIC)	Vancomycin 0.50 µg/mL (MIC)	[38]
	Antibacterial	Agar diffusion assay/*C. neoformans* ATCC 9013	200 µg/mL (MIC)	Amphotericin B 0.25 µg/mL (MIC)	[38]
	Antitumor	MTT/Vero cells	45.07 µM (IC_50_)	Ellipticine 4.55 µM (IC_50_)	[38]
3-Hydroxy-2,5-dimethylphenyl 2,4-dihydroxy-3,6-dimethylbenzoate (**9**)	Antibacterial	Microbroth dilution/*S. aureus*	250 µg/mL (MIC)	Vancomycin and tetracycline	[49]
	Antibacterial	Microbroth dilution/*B. subtilis*	25 µg/mL (MIC)	Vancomycin and tetracycline	[49]
	Antibacterial	Microbroth dilution/*P. aeruginosa*	25 µg/mL (MIC)	Vancomycin and tetracycline	[49]
Colletotric acid C (**11**)	Antibacterial	Agar diffusion assay/*B. subtilis*	9.70 µg/mL (MIC)	Ampicillin 0.07 µg/mL (MIC)	[46]
Agonodepside A (**12**)	Enoyl-ACP reductase inhibition	Fluorometric InhA assay/*M. tuberculosis* InhA	74 µM (IC_50_)	Triclosan 3 µM (IC_50_)	[50]
Thielavin Z_7_ (**15**)	Antifouling	*Balanus Amphitrite*(cyprid larvae)	3.20 µM (EC_50_)	Butenolide 4.62 µM (EC_50_)	[52]
Sterenin J (**16**)	α-Glucosidase inhibitory	Colorimetric/α-Glucosidase	65.70 µM (IC_50_)	Acarbose 640.88 µM (IC_50_)	[53]
Sterenin E (**17**)	α-Glucosidase inhibitory	Colorimetric/α-Glucosidase	7.62 µM (IC_50_)	Acarbose 640.88 µM (IC_50_)	[53]
Sterenin F (**18**)	α-Glucosidase inhibitory	Colorimetric/α-Glucosidase	3.06 µM (IC_50_)	Acarbose 640.88 µM (IC_50_)	[53]
Sterenin G (**19**)	α-Glucosidase inhibitory	Colorimetric/α-Glucosidase	6.03 µM (IC_50_)	Acarbose 640.88 µM (IC_50_)	[53]
Sterenin H (**20**)	α-Glucosidase inhibitory	Colorimetric/α-Glucosidase	22.70 µM (IC_50_)	Acarbose 640.88 µM (IC_50_)	[53]
Sterenin I (**21**)	α-Glucosidase inhibitory	Colorimetric/α-Glucosidase	72.50 µM (IC_50_)	Acarbose 640.88 µM (IC_50_)	[53]
MS-3 (**22**)	Antibacterial	Plate diffusion/*B.* *cereus*	25 mm (IZD)	Streptomycin 28 mm (IZD) Penicillin G 28 mm (IZD)	[57]
	Antibacterial	Plate diffusion/*B. subtilis*	25 mm (IZD)	Streptomycin 32 mm (IZD) Penicillin G 28 mm (IZD)	[57]
	Antibacterial	Plate diffusion/*S. aureus*	28 mm (IZD)	Streptomycin 30 mm (IZD) Penicillin G 27 mm (IZD)	[57]
	α-Glucosidase inhibitory	Colorimetric/α-Glucosidase inhibitor	23.82 µM (IC_50_)	Acarbose 640.88 µM (IC_50_)	[53]
4′-Hydroxy-5′-methoxy-6′-(3″-methyl-2″-butenyl)-phenyl-2,4-dihydroxy-6-methyl-benzoate/ 4-Hydroxy-3-methoxy-2-(3-methylbut-2-en-1-yl)phenyl 2,4-dihydroxy-6-methylbenzoate (**23**)	Anti-inflammatory NO inhibitory potential	LPS-induced macrophages RAW 264.7	19.17 µM (IC_50_)	Hydrocortisone 48.15 µM (IC_50_)	[40]
	Antibacterial	Agar plate diffusion/MRSA	25 µg/mL (MIC)	Ancomycin 1.0 µg/mL (MIC)	[40]
	Antibacterial	Agar plate diffusion/*S. aureus*	25 µg/mL (MIC)	Ancomycin 1.0 µg/mL (MIC)	[40]
	Antibacterial	Agar plate diffusion/*B. subtilis*	25 µg/mL (MIC)	Ancomycin 0.5 µg/mL (MIC)	[40]
	Antitumor	MTT/A549	13.14 µM (IC_50_)	Cisplatin 14.33 µM (IC_50_)	[40]
	Antitumor	MTT/HepG2	49.02 µM (IC_50_)	Cisplatin 18.74 µM (IC_50_)	[40]
	α-Glucosidase inhibitory	Colorimetric/α-Glucosidase inhibitor	14.17 µM (IC_50_)	Acarbose 640.88 µM (IC_50_)	[53]
4′-Hydroxy-6′-(3″-methyl-2″-butenyl)-phenyl-2,4-dihydroxy-6-methyl-benzoate (**24**)	Antibacterial	Agar plate diffusion/MRSA	25 µg/mL (MIC)	Ancomycin 1.0 µg/mL (MIC)	[40]
	Antibacterial	Agar plate diffusion/*S. aureus*	25 µg/mL (MIC)	Ancomycin 1.0 µg/mL (MIC)	[40]
	Antibacterial	Agar plate diffusion/*B. subtilis*	50 µg/mL (MIC)	Ancomycin 0.5 µg/mL (MIC)	[40]
KS-501a (**27**)	Antimalarial activity	Microculture radioisotope technique/*Plasmodium falciparum* K1	9.9 µM (IC_50_)	Dihydroartemisinin 0.0039 µM (IC_50_)	[41]
	Antitumor	MTT/KB	13.0 µM (IC_50_)	Ellipticine 1.99 µM (IC_50_)	[41]
	Antitumor	MTT/BC	8.8 µM (IC_50_)	Ellipticine 0.49 µM (IC_50_)	[41]
	Antitumor	MTT/NC1-H187	13.6 µM (IC_50_)	Ellipticine 1.77 µM (IC_50_)	[41]
	Antitumor	MTT/Vero cells	34.3 µM (IC_50_)	Ellipticine 1.94 µM (IC_50_)	[41]
KS-501a-2-O-β-D- galactopyranose (**28**)	Antitumor	MTT/KB	>25 µM (IC_50_)	Ellipticine 1.99 µM (IC_50_)	[41]
	Antitumor	MTT/BC	4.4 µM (IC_50_)	Ellipticine 0.49 µM (IC_50_)	[41]
	Antitumor	MTT/NC1-H187	13.9 µM (IC_50_)	Ellipticine 1.77 µM (IC_50_)	[41]
KS-501a-2-O-β-D- digalactopyranose (**29**)	Antitumor	MTT/KB	>25 µM (IC_50_)	Ellipticine 1.99 µM (IC_50_)	[41]
	Antitumor	MTT/BC	20.2 µM (IC_50_)	Ellipticine 0.49 µM (IC_50_)	[41]
	Antitumor	MTT/NC1-H187	>25 µM (IC_50_)	Ellipticine 1.77 µM (IC_50_)	[41]
	Antitumor	MTT/Vero cells	32.1 µM (IC_50_)	Ellipticine 1.94 µM (IC_50_)	[41]
Arenicolin A (**30**)	Antitumor	Immunocytochemistry ICC/HCT-116	7.3 µM (IC_50_)	5-FU 6.5 µM (IC_50_)	[42]
	Antitumor	Immunocytochemistry ICC/IMR-32	6.0 µM (IC_50_)	5-FU 5.7 µM (IC_50_)	[42]
	Antitumor	Immunocytochemistry ICC/BT-474	9.7 µM (IC_50_)	-	[42]
Sterenin A (**38**)	11β-HSD inhibitory	Luminescence immunoassay/HTRF cortisol	240 nM (IC_50_)	-	[64]
	α-Glucosidase inhibitory	Colorimetric/α-Glucosidase	25.10 µM (IC_50_)	Acarbose 640.88 µM (IC_50_)	[53]
Sterenin B (**39**)	11β-HSD inhibitory	Luminescence immunoassay/HTRF cortisol	6600 nM (IC_50_)	-	[64]
	α-Glucosidase inhibitory	Colorimetric/α-Glucosidase inhibitor	12.23 µM (IC_50_)	Acarbose 640.88 µM (IC_50_)	[53]
Sterenin C (**40**)	11β-HSD inhibitory	Luminescence immunoassay/HTRF cortisol	230 nM (IC_50_)	-	[64]
	α-Glucosidase inhibitory	Colorimetric/α-Glucosidase inhibitor	3.31 µM (IC_50_)	Acarbose 640.88 µM (IC_50_)	[53]
Sterenin D (**41**)	11β-HSD inhibitory	Luminescence immunoassay/HTRF cortisol	2600 nM (IC_50_)	-	[64]
	Antifungal	Plate diffusion/*B. cinerea*	50 µg/mL (MFC)	Rovral 10 µg/mL (MFC)	[58]
	Antifungal	Plate diffusion/*B. cinerea*	10 µg/mL (MIC)	Rovral 1 µg/mL (MFC)	[58]
Sterenin K (**42**)	α-Glucosidase inhibitory	Colorimetric/α-Glucosidase	36.64 µM (IC_50_)	Acarbose 640.88 µM (IC_50_)	[53]
Sterenin L (**43**)	α-Glucosidase inhibitory	Colorimetric/α-Glucosidase	13.09 µM (IC_50_)	Acarbose 640.88 µM (IC_50_)	[53]
Sterenin M (**44**)	α-Glucosidase inhibitory	Colorimetric/α-Glucosidase	27.52 µM (IC_50_)	Acarbose 640.88 µM (IC_50_)	[53]
Colletotric acid A (**45**)	Antibacterial	Agar diffusion assay/*B. subtilis*	25 µg/mL (MIC)	Ampicillin 0.05 µg/mL (MIC)	[65]
	Antibacterial	Agar diffusion assay/*B. subtilis*	6.55 µg/mL (MIC)	Ampicillin 0.07 µg/mL (MIC)	[46]
	Antibacterial	Agar diffusion assay/*S. aureus*	50 µg/mL (MIC)	Ampicillin 0.5 µg/mL (MIC)	[65]
	Antibacterial	Agar diffusion assay/*S. lutea*	50 µg/mL (MIC)	Ampicillin 0.01 µg/mL (MIC)	[65]
	Antibacterial	Agar diffusion assay/*H. lutea*	50 µg/mL (MIC)	Triadimefon 20 µg/mL (MIC)	[65]
	Antibacterial	Agar diffusion assay/*P. aeruginosa*	3.27 µg/mL (MIC)	Ampicillin 0.15 µg/mL (MIC)	[46]
	Antibacterial	Agar diffusion assay/MRSA	6.28 µg/mL (MIC)	Ampicillin 0.15 µg/mL (MIC)	[46]
	Antibacterial	Agar diffusion assay/*S. typhimurium*	26.20 µg/mL (MIC)	Ampicillin 0.31 µg/mL (MIC)	[46]
	Antifungal	Agar diffusion assay/*C. albicans*	3.27 µg/mL (MIC)	Ketoconazole 0.10 µg/mL (MIC)	[46]
	Antitumor	MTT/MDA-MB-435	37.01 µM (IC_50_)	Epirubicin 0.26 µM (IC_50_)	[46]
	Antitumor	MTT/A549	37.73 µM (IC_50_)	Epirubicin 5.60 µM (IC_50_)	[46]
Colletotric acid B (**46**)	Antibacterial	Agar diffusion assay/*P. aeruginosa*	1.67 µg/mL (MIC)	Ampicillin 0.15 µg/mL (MIC)	[46]
	Antibacterial	Agar diffusion assay/MRSA	3.36 µg/mL (MIC)	Ampicillin 0.15 µg/mL (MIC)	[46]
	Antitumor	MTT/MDA-MB-435	16.82 µM (IC_50_)	Epirubicin 0.26 µM (IC_50_)	[46]
	Antitumor	MTT/A549	20.75 µM (IC_50_)	Epirubicin 5.60 µM (IC_50_)	[46]
	Antibacterial	Agar diffusion assay/*B. subtilis*	26.90 µg/mL (MIC)	Ampicillin 0.07 µg/mL (MIC)	[46]
Thielavin A (**48**)	Phospholipase A (PLA) inhibition	Rat PLA_2_-II	43 µM (IC_50_)	Mepacrine 320 µM (IC_50_) p-Bromophenacyl bromide 6.7 µM (IC_50_) Manoalide 2.0 µM (IC_50_)	[70]
		Human PLA_2_-II	29 µM (IC_50_)	Mepacrine 76 µM (IC_50_) p-Bromophenacyl bromide 34 µM (IC_50_) Manoalide 1.5 µM (IC_50_)	
	Antifouling	*Balanus amphitrite* (cyprid larvae)	54.99 µM (EC_50_)	Butenolide 4.62 µM (EC_50_)	[52]
	Anti-diabetic	Colorimetric/α-Glucosidase inhibitor (αGHY)	23.8 µM (IC_50_)	Acarbose 545 µM (IC_50_	[67]
	Prostaglandin synthesis inhibition	Conversion of AA into PGH_2_	10 µM (IC_50_)	Indomethacin 30 µM (IC_50_)	[69,81]
		Conversion of PGH_2_ into PGE_2_	40 µM (IC_50_)	Indomethacin 130 µM (IC_50_)	
		Conversion of PGE_2_ into TXA_2_	150 µM (IC_50_)	Imidazole 200 µM (IC_50_)	
Thielavin B (**49**)	Phospolipase A (PLA) inhibition	Rat PLA_2_-II	1.3 µM (IC_50_)	Mepacrine 320 µM (IC_50_) p-Bromophenacyl bromide 6.7 µM (IC_50_) Manoalide 2.0 µM (IC_50_)	[70]
		Human PLA_2_-II	2.4 µM (IC_50_)	Mepacrine 76 µM (IC_50_) p-Bromophenacyl bromide 34 µM (IC_50_) Manoalide 1.5 µM (IC_50_)	
	Prostaglandin synthesis inhibition	Conversion of AA into PGH_2_	40 µM (IC_50_)	Indomethacin 30 µM (IC_50_)	[69,81]
		Conversion of PGH_2_ into PGE_2_	9 µM (IC_50_)	Indomethacin 130 µM (IC_50_)	
		Conversion of PGE_2_ into TXA_2_	350 µM (IC_50_)	Imidazole 200 µM (IC_50_)	
Thielavin B methyl ester (**50**)	Antitumor	SRB/MCF-7	7.3 µM (IC_50_)	0.07 µM (IC_50_)	[48]
	Antitumor	SRB/H460	6.6 µM (IC_50_)	< 0.01 µM (IC_50_)	[48]
	Antitumor	SRB/SF268	8.1 µM (IC_50_)	0.04 µM (IC_50_)	[48]
Thielavin C (**51**)	PLA inhibition	Rat PLA_2_-II	0.46 µM (IC_50_)	Mepacrine 320 µM (IC_50_) p-Bromophenacyl bromide 6.7 µM (IC_50_) Manoalide 2.0 µM (IC_50_)	[70]
		Human PLA_2_-II	2.1 µM (IC_50_)	Mepacrine 76 µM (IC_50_) p-Bromophenacyl bromide 34 µM (IC_50_) Manoalide 1.5 µM (IC_50_)	
Thielavin D (**52**)	PLA inhibition	Rat PLA_2_-II	1.1 µM (IC_50_)	Mepacrine 320 µM (IC_50_) p-Bromophenacyl bromide 6.7 µM (IC_50_) Manoalide 2.0 µM (IC_50_)	[70]
		Human PLA_2_-II	6.2 µM (IC_50_)	Mepacrine 76 µM (IC_50_) p-Bromophenacyl bromide 34 µM (IC_50_) Manoalide 1.5 µM (IC_50_)	
Thielavin E (**53**)	PLA inhibition	Rat PLA_2_-II	4.5 µM (IC_50_)	Mepacrine 320 µM (IC_50_) p-Bromophenacyl bromide 6.7 µM (IC_50_) Manoalide 2.0 µM (IC_50_)	[70]
		Human PLA_2_-II	9.3 µM (IC_50_)	Mepacrine 76 µM (IC_50_) p-Bromophenacyl bromide 34 µM (IC_50_) Manoalide 1.5 µM (IC_50_)	
Thielavin H (**56**)	Antifouling	*Balanus amphitrite*	12.64 µM (EC_50_)	Butenolide 4.62 µM (EC_50_)	[52]
Thielavin J (**58**)	Anti-diabetic	Colorimetric/α-Glucosidase inhibitor (αGHY)	15.8 µM (IC_50_)	Acarbose 545 µM (IC_50_)	[67]
Thielavin K (**59**)	Anti-diabetic	Colorimetric/α-Glucosidase inhibitor (αGHY)	22.1 µM (IC_50_)	Acarbose 545 µM (IC_50_)	[67]
Thielavin S (**66**)	Antibacterial	Microbroth dilution/*S. aureus* ATCC 25923	100 µg/mL (MIC)	Tetracycline 3.12 µg/mL (MIC)	[19]
Thielavin T (**67**)	Antimicrobial	Microbroth dilution assay/*S. aureus* ATCC 25923	6.25 µg/mL (MIC)	Tetracycline 3.12 µg/mL (MIC)	[19]
Thielavin U (**68**)	Antibacterial	Microbroth dilution assay/*S. aureus* ATCC 25923	50 µg/mL (MIC)	Tetracycline 3.12 µg/mL (MIC)	[19]
Thielavin V (**69**)	Antibacterial	Microbroth dilution assay/*S. aureus* ATCC 25923	25 µg/mL (MIC)	Tetracycline 3.12 µg/mL (MIC)	[19]
Thielavin W (**70**)	Antifouling	*Balanus Amphitrite*(cyprid larvae)	2.95 µM (EC_50_)	Butenolide 4.62 µM (EC_50_)	[52]
Thielavin X (**71**)	Antifouling	*Balanus Amphitrite*(cyprid larvae)	3.13 µM (EC_50_)	Butenolide 4.62 µM (EC_50_)	[52]
Thielavin Y (**72**)	Antifouling	*Balanus Amphitrite*(cyprid larvae)	5.78 µM (EC_50_)	Butenolide 4.62 µM (EC_50_)	[52]
Thielavin Z_2_ (**75**)	Antifouling	*Balanus Amphitrite*(cyprid larvae)	69.19 µM (EC_50_)	Butenolide 4.62 µM (EC_50_)	[52]
Thielavin Z_3_ (**76**)	Antifouling	*Balanus Amphitrite* (cyprid larvae)	4.23 µM (EC_50_)	Butenolide 4.62 µM (EC_50_)	[52]
Thielavin Z_4_ (**77**)	Antifouling	*Balanus Amphitrite*(cyprid larvae)	50.50 µM (EC_50_)	Butenolide 4.62 µM (EC_50_)	[52]
Thielavin Z_5_ (**78**)	Antifouling	*Balanus Amphitrite*(cyprid larvae)	25.86 µM (EC_50_)	Butenolide 4.62 µM (EC_50_)	[52]
Thielavin Z_6_ (**79**)	Antifouling	*Balanus Amphitrite*(cyprid larvae)	17.86 µM (EC_50_)	Butenolide 4.62 µM (EC_50_)	[52]
3-Hydroxy-2,5-dimethylphenyl 4-[(2,4-dihydroxy-3,6-dimethylbenzoyl)oxy]-2-hydroxy-3,6- dimethylbenzoate (**80**)	Antibacterial	Microbroth dilution/*S. aureus*	250 µg/mL (MIC)	Vancomycin and tetracycline	[49]
	Antibacterial	Microbroth dilution/*B. subtilis*	250 µg/mL (MIC)	Vancomycin and tetracycline	[49]
	Antibacterial	Microbroth dilution/*P. aeruginosa*	250 µg/mL (MIC)	Vancomycin and tetracycline	[49]
	Antibacterial	Microbroth dilution/*E. coli*	250 µg/mL (MIC)	Vancomycin and tetracycline	[49]
3-Hydroxy-2,4,5-trimethylphenyl 4-[(2,4-dihydroxy-3,6-dimethylbenzoyl)oxy]-2-hydroxy-3,6- dimethylbenzoate (**81**)	Antibacterial	Microbroth dilution/*S. aureus*	250 µg/mL (MIC)	Vancomycin and tetracycline	[49]
	Antibacterial	Microbroth dilution/*B. subtilis*	25 µg/mL (MIC)	Vancomycin and tetracycline	[49]
	Antibacterial	Microbroth dilution/*P. aeruginosa*	25 µg/mL (MIC)	Vancomycin and tetracycline	[49]
	Antibacterial	Microbroth dilution/*E. coli*	250 µg/mL (MIC)	Vancomycin and tetracycline	[49]
Amidepsine D (**86**)	DGAT1 inhibition	Rat liver microsomes	17.5 µM (IC_50_)	-	[74]
	DGAT2 inhibition	Rat liver microsomes	30 µM (IC_50_)		[71,82]
	Triacylglycerol inhibition	Raji cells	2.82 µM (IC_50_)	-	[74]
	Antibacterial	Disk diffusion/*B. subtilis* ATCC6633	8.0 mm (IZD)	-	[74]
Amidepsine A (**88**)	DGAT1 inhibition	Rat liver microsomes	10.2 µM (IC_50_)	-	[74]
	DGAT2 inhibition	Rat liver microsomes	70 µM (IC_50_)	-	[71,82]
	Triacylglycerol inhibition	Raji cells	15.5 µM (IC_50_)	-	[74]
	Antibacterial	Disk diffusion/*B. subtilis* ATCC6633	11.0 mm (IZD)	-	[74]
Amidepsine B (**89**)	DGAT1 inhibition	Rat liver microsomes	19.2 µM (IC_50_)	-	[74]
	DGAT2 inhibition	Rat liver microsomes	60 µM (IC_50_)	-	[71,82]
	Triacylglycerol inhibition	Raji cells	3.35 µM (IC_50_)	-	[74]
	Antibacterial	Disk diffusion/*B. subtilis* ATCC6633	7.0 mm (IZD)	-	[74]
Amidepsine C (**90**)	DGAT1 inhibition	Rat liver microsomes	51.6 µM (IC_50_)	-	[74]
	DGAT2 inhibition	Rat liver microsomes	100 µM (IC_50_)	-	[71,82]
	Triacylglycerol inhibition	Raji cells	17.2 µM (IC_50_)	-	[74]
	Antibacterial	Disk diffusion/*B. subtilis* ATCC6633	9.0 mm (IZD)	-	[74]
Amidepsine E (**91**)	Triacylglycerol inhibition	Raji cells	91 µM (IC_50_)	-	[76]
	DGAT1 inhibition	Rat liver microsomes	124 µM (IC_50_)	-	[76]
Amidepsine J (**96**)	DGAT1 inhibition	Rat liver microsomes	40 µM (IC_50_)	-	[71]
	DGAT2 inhibition	Rat liver microsomes	40 µM (IC_50_)		[71]
CJ-21,164 (**97**)	G6Pase inhibition	Colorimetric assay	102% (% inhibition)		[77]
	Glucose output inhibition	Colorimetric assay	81% (% inhibition)	-	[77]
Thielocin A1i (**99**)	PLA inhibition	Human PLA_2_-II	6.2 µM (IC_50_)	-	[78,80,83,84]
		Human PLA_2_-I	140 µM (IC_50_)	-	
		Ki value human PLA_2_-II	12 µM	-	
		Rat PLA_2_-II	0.0033 µM (IC_50_)	Mepacrine 240 µM (IC_50_)	
		*Vipera russelli* venom PLA_2_-II	17 µM (IC_50_)		
		*Crotalus adamanteus* venom PLA_2_-II	17 µM (IC_50_)		
		Porcine pancreas PLA_2_-I	63 µM (IC_50_)		
		Rat PLA_2_-I	21 µM (IC_50_)	Mepacrine 135 µM (IC_50_)	
		Bee venom PLA_2_-I	2 µM (IC_50_)		
		Naja naja venom PLA_2_-I	7.1 µM (IC_50_)		
		*N. mocambique* venom PLA_2_-I	9.3 µM (IC_50_)		
		Bee venom PLA_2_	1.4 µM (IC_50_)	*p*-Bromophenacyl bromide 80 µM (IC_50_)	[83,84]
		Bee venom PLA_2_-induced paw edema	42.4 (mg)		
		Ki value Bee venom PLA2	0.57 µM		
Thielocin A2α (**100**)	PLA inhibition	Rat PLA2-II	0.051 µM (IC_50_)	Mepacrine 320 µM (IC_50_) *p*-Bromophenacyl bromide 6.7 µM (IC_50_) Manoalide 2.0 µM (IC_50_)	[70]
		Human PLA_2_-II	0.31 µM (IC_50_)	Mepacrine 76 µM (IC_50_) *p*-Bromophenacyl bromide 34 µM (IC_50_) Manoalide 1.5 µM (IC_50_)	
Thielocin A2β (**101**)	PLA inhibition	Rat PLA2-II	0.038 µM (IC_50_)	Mepacrine 320 µM (IC_50_) *p*-Bromophenacyl bromide 6.7 µM (IC_50_) Manoalide 2.0 µM (IC_50_)	[70]
		Human PLA_2_-II	0.24 µM (IC_50_)	Mepacrine 76 µM (IC_50_) *p*-Bromophenacyl bromide 34 µM (IC_50_) Manoalide 1.5 µM (IC_50_)	[70]
		Rat PLA2-II	0.032 µM (IC_50_)	Mepacrine 320 µM (IC_50_) *p*-Bromophenacyl bromide 6.7 µM (IC_50_) Manoalide 2.0 µM (IC_50_)	[70]
		Human PLA_2_-II	0.39 µM (IC_50_)	Mepacrine 76 µM (IC_50_) *p*-Bromophenacyl bromide 34 µM (IC_50_) Manoalide 1.5 µM (IC_50_)	
Thielocin B1 (**105**)	PLA inhibition	Rat PLA2-II	0.0078 µM (IC_50_)	Mepacrine 320 µM (IC_50_) *p*-Bromophenacyl bromide 6.7 µM (IC_50_) Manoalide 2.0 µM (IC_50_)	[70]
		Human PLA_2_-II	0.17 µM (IC_50_)	Mepacrine 76 µM (IC_50_) *p*-Bromophenacyl bromide 34 µM (IC_50_) Manoalide 1.5 µM (IC_50_)	
Thielocin B2 (**106**)	PLA inhibition	Rat PLA2-II	0.070 µM (IC_50_)	Mepacrine 320 µM (IC_50_) *p*-Bromophenacyl bromide 6.7 µM (IC_50_) Manoalide 2.0 µM (IC_50_)	[70]
		Human PLA_2_-II	2.7 µM (IC_50_)	Mepacrine 76 µM (IC_50_) *p*-Bromophenacyl bromide 34 µM (IC_50_) Manoalide 1.5 µM (IC_50_)	
Thielocin B3 (**107**)	PLA inhibition	Human PLA2-I	18 µM (IC_50_)	-	[70,80]
		Rat PLA_2_-II	0.012 µM (IC_50_)	Mepacrine 320 µM (IC_50_) *p*-Bromophenacyl bromide 6.7 µM (IC_50_) Manoalide 2.0 µM (IC_50_)	
		Human PLA_2_-II	0.076 µM (IC_50_)	Mepacrine 76 µM (IC_50_) *p*-Bromophenacyl bromide 34 µM (IC_50_) Manoalide 1.5 µM (IC_50_)	
		Rat PLA_2_-I	2.8 µM (IC_50_)	-	
		Mean Ki	0.98 µM	-	
		Snake venom PLA_2_	0.0045 µM (IC_50_) 1.6 µM (ED_50_)	-	
		Exudate volume after carrageenan	1.60 mL (conc. 1 mg/kg) 1.15 mL (conc. 3 mg/kg)	Indomethacin 1.08 mL (conc. 1 mg/kg) Dexamethasone 0.60 mL (conc. 0.1 mg/kg)	
		PLA_2_ activityin pleural exudate after carrageenan	2.22 pmol/minute/mL (conc. 1 mg/kg) 0.76 pmol/minute/mL (conc. 3 mg/kg)	Indomethacin 7.36 pmol/minute/mL (conc. 1 mg/kg) Dexamethasone 7.94 pmol/minute/mL (conc. 0.1 mg/kg)	
Thielocin B3 monomethyl ester B (**108**)	PLA inhibition	Human PLA_2_-II	0.20 µM (IC_50_)	-	[80]
		Snake venom PLA_2_	0.032 µM (IC_50_) 5.2 µM (ED_50_)	-	
Thielocin B3 monomethyl ester C (**109**)	PLA inhibition	Human PLA_2_-II	0.28 µM (IC_50_)	-	[80]
		Snake venom PLA_2_	0.31 µM (IC_50_) 5.2 µM (ED_50_)	-	
Thielocin B3 monomethyl ester D (**110**)	PLA inhibition	Human PLA_2_-II	51 µM (IC_50_)	-	[80]
		Snake venom PLA_2_	>100 µM (IC_50_) 7.6 µM (ED_50_)	-	

## Supplementary Materials

The following are available online at https://www.mdpi.com/article/10.3390/metabo11100683/s1, Table S1: Physical and spectral data of fungal depsides.

## Abbreviations

ACP: acyl carrier protein; AT: acyl transferase; 11 β-HSD1; 11 β-hydroxysteroid dehydrogenase type 1; 11 β-HSD2:11 β-hydroxysteroid dehydrogenase type 2; 5-FU: 5-fluorouracil; APPIHRMS: atmospheric pressure photoionization high-resolution; B16-F10: murine melanoma cell line; BC: human breast cancer; BT-474: ductal carcinoma; CaM: Calmodulin; CaM-PDE: calmodulin-dependent cyclic-nucleotide phosphodiesterase; CaM-PDEI: Ca2+/CaM dependent phosphodiesterase inhibitory; CCF-STTG1: human astrocytoma cells; CCF-STTG1: human astrocytoma; CD45: leucocyte common antigen; CTC: 5-cyano-2,3-bis(4-methylphenyl)-2H-tetrazolium chloride; DGAT; diacylglycerol acyltransferase; DGATI: diacylglycerol acyltransferase inhibitory; DPPH: 2-diphenyl-1-picrylhydrazyl; EC_50_: Inhibition of settlement of 50% of the larval population; FSW: filtered sea water; G6Pase: D-glucose-6-phosphate phosphohydrolase; H^+^/K^+^ ATPase: hydrogen/potassium adenosine triphosphatase; H46: human non-small cell lung carcinoma; hCMV: human cytomegalovirus; HCT-116: colorectal carcinoma; HepG2: human liver cancer cells: HLE: human leukocyte elastase; HSV-1: *Herpes simplex* virus type 1; HTRF: homogeneous time resolved fluorescence; IC_50_: concentration required to inhibit cell growth by 50%; IC: immunocytochemistry and binding; ID_50_: dose producing a 50% inhibition; IDO: indoleamine 2,3-dioxygenase; IDOI: indoleamine 2,3-dioxygenase inhibitory; IMR-32: neuroblastoma; ISCID: collision-induced dissociation; IZDs: inhibition zone diameters; KB: human epidermoid carcinoma cell; KS: ketosynthase; LAR: leukocyte common antigen-related; MBCs: minimal bactericidal concentrations; CMeT: methyl transferase; MCF-7: human breast adenocarcinoma; MFC: minimal fungicidal concentration; MRSA: methicillin-resistant *S. aureus*; MS: metabolic syndrome; MTT: (3-(4,5-dimethylthiazol-2-yl))-2,5-diphenyl-2H-tetrazolium bromide; NA-STZ: nicotinamide-streptozotocin; Na^+^/K^+^-ATPase: sodium/potassium adenosine triphosphatase; NCI-H187: small-cell lung cancer; PC: phosphatidylcholine; PE: phosphatidylethanolamine; PKSs: polyketide synthases; NR-PKSs: non-reducing polyketide synthases PLA2II: phospholipase A2; PMNLs: polymorphonuclear leukocytes; pNPP: *p*-nitrophenyl phosphate; PTP1B: protein-tyrosine phosphatase 1B; PTP-1BIs: PTP1B inhibitors; SAM: S-adenosylmethionine; SF268: human astrocytoma: SHP-2: Src homology phosphatase-2; SPA: scintillation proximity assay; SRB: sulforhodamine B; T2DM: type 2 diabetes mellitus; TCPTP: T-cell protein tyrosine phosphatase; TXA2: thromboxane A2; Vero: African green monkey kidney fibroblast; αGHBs: *Bacillus stearothermophilus* α-glucosidase; αGHY: *Saccharomyces cerevisiae* α-glucosidase.
