# Supplementary material for: Fungal Depsides—Naturally Inspiring Molecules: Biosynthesis, Structural Characterization, and Biological Activities
Source: Metabolites. 2021 Oct 5;11(10):683. doi: 10.3390/metabo11100683 (PMC8540757; doi:10.3390/metabo11100683)
Supplement: Supplementary file 1 [file metabolites-11-00683-s001.zip › metabolites-1322970-supplementary.pdf]

metabolites

MDPI

Review Article

Fungal Depsides Naturally Inspiring Molecules: Biosynthesis, Structural Characterization, and Biological Activities

Sabrin R.M. Ibrahim[1,2*], Alaa Sirwi[3], Basma G. Eid[4], Shaimaa G.A. Mohamed[5], Gamal A. Mohamed[3,6]

1 Batterjee Medical College, Preparatory Year Program, Jeddah 21442, Saudi Arabia; sabrin.ibrahim@bmc.edu.sa)
2 Department of Pharmacognosy, Faculty of Pharmacy, Assiut University, Assiut 71526, Egypt; sabreen.ibrahim@pharm.aun.edu.eg
3 Department of Natural Products and Alternative Medicine, Faculty of Pharmacy, King Abdulaziz University, Jeddah 21589, Saudi Arabia; asirwi@kau.edu.sa; gahussein@kau.edu.sa
4 Department of Pharmacology and Toxicology, Faculty of Pharmacy, King Abdulaziz University, Jeddah 21589, Saudi Arabia; beid@kau.edu.sa
5 Faculty of Dentistry, British University, El Sherouk City, Suez Desert Road, Cairo 11837, Egypt; shaimaag1973@gmail.com
6 Department of Pharmacognosy, Faculty of Pharmacy, Al-Azhar University, Assiut Branch, Assiut 71524, Egypt; gamals2001@yahoo.com
* Correspondence: sabrin.ibrahim@bmc.edu.sa; sabrinshaur@gmail.com; Tel.: +966 581183034

Received: date; Accepted: date; Published: date

Table S1: Physical and spectral data of fungal depsides

**Lecanoric acid (1)**	
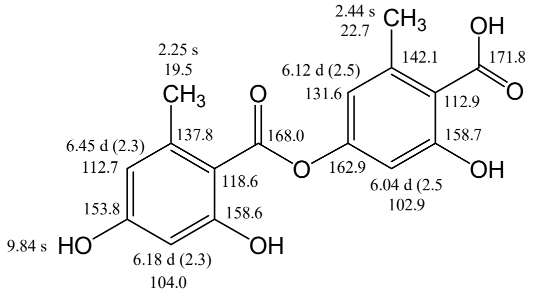	Colorless solid; UV (MeCN) λ_{max}: 236, 258, 290 nm; ESIHCDHRMS: *m/z* 317.0662, [M-H]⁻ (calcd for $C_{16}H_{13}O_7$, *m/z* 317.0661; ^1H NMR and ^{13}C NMR (600 and 150 MHz, DMSO-*d*$_6$) [37].
**Ethyl lecanorate (2)**	
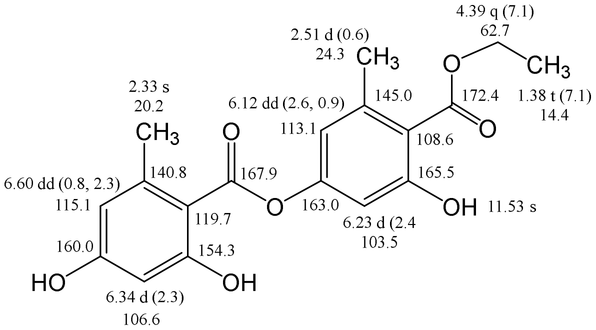	Colorless solid; UV (MeCN) λ_{max}: 238, 257, 299 nm; ESIHCDHRMS: *m/z* 345.0978 [M-H]⁻ (calcd for $C_{18}H_{17}O_7$, 345.0974); ^1H and ^{13}C NMR (600 and 150 MHz, MeCN-*d*$_3$) [37].
**Aspergiside A (3)**	
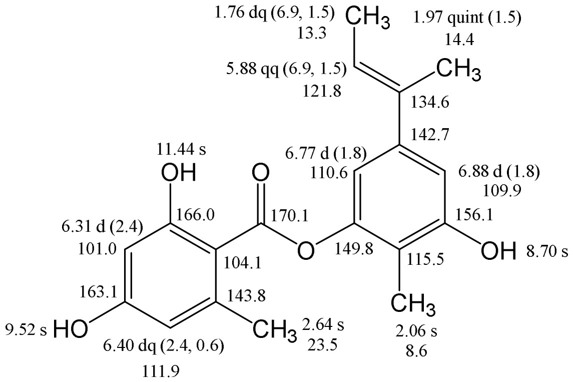	Colorless solid; mp 187-188 °C; UV (MeOH) λ_{max} (log *ε*): 263 (3.57), 300 (3.30) nm; IR (neat) γ^{max} 3342, 1726 cm^{-1}; HRESIMS: *m/z* [M+Na]⁺ 351.1208 (calcd for $C_{19}H_{20}O_5Na$, 351.1208); ^1H and ^{13}C NMR (300 and 75 MHz, acetone-*d*$_6$) [38].
**Aspergiside B (4)**	
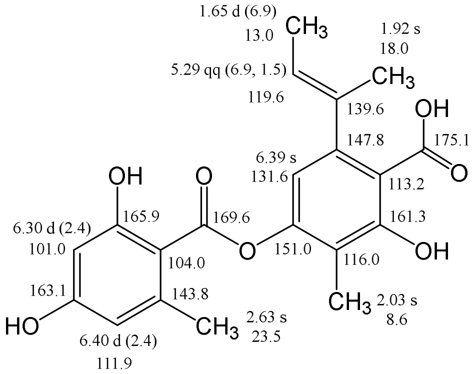	Colorless gum; UV (MeOH) λ_{max} (log *ε*): 262 (3.49), 308 (3.24) nm; IR (neat) γ_{max} 3406, 1723, 1710 cm^{-1}; HRESIMS: *m/z* 411.0843 [M+K]⁺ (calcd for $C_{20}H_{20}O_7K$, 411.0846); ^1H and ^{13}C NMR (500 and 125 MHz, acetone-*d*$_6$) [38].
**Aspergiside C (5)**	

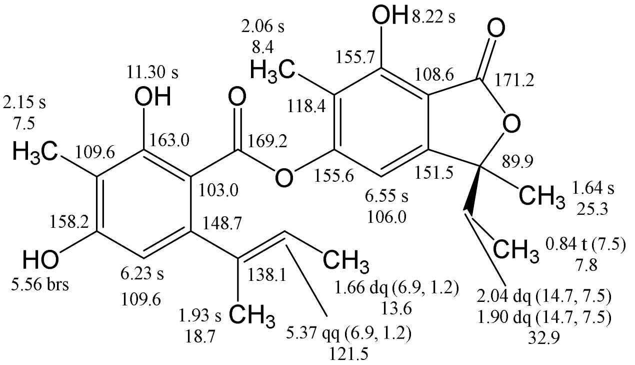	Colorless solid; mp 211-213 °C; $[\alpha]_D^{24}$ D -57.0 (*c* 0.1, MeOH); UV (MeOH) λ_{max} (log *ε*): 261 (3.56), 300 (3.01) nm; IR (neat) $\gamma_{max:}$ 3406, 1736, 1721 cm^{-1}; HRESIMS: *m/z* 449.1576 $[M+Na]^+$ (calcd for $C_{24}H_{26}O_7Na$, 449.1576); ^1H and ^{13}C NMR (300 and 75 MHz, $CDCl_3$) [38].
**Lecanorin D (6)**	
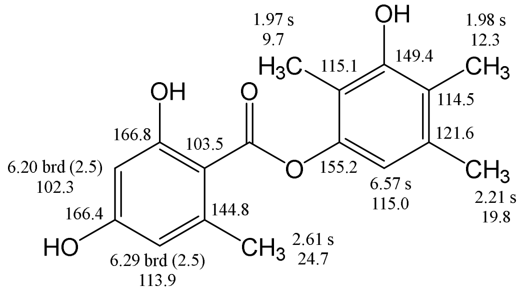	White powder; UV (MeOH) $\lambda_{max:}$ 217, 268, 305 nm; HRESIMS: *m/z* 301.1078 $[M-H]^-$ (calcd for $C_{17}H_{17}O_5$, 301.1076) ; ^1H NMR and ^{13}C NMR (800 and 200 MHz, CD_3OD) [19].
**Lecanorin E (7)**	
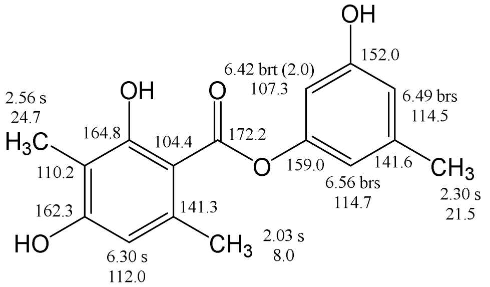	White powder; UV (MeOH) $\lambda_{max:}$ 222, 269, 305 nm; HRESIMS *m/z:* 287.0920 $[M-H]^-$ (calcd for $C_{16}H_{15}O_5$, 287.0920); ^1H NMR and ^{13}C NMR (500 and 125 MHz, CD_3OD) [19].
**Lecanorin F (8)**	
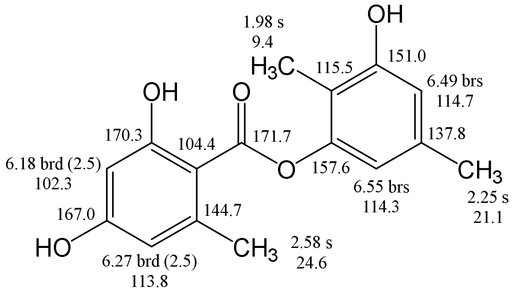	White powder; UV (MeOH) $\lambda_{max:}$ 218, 275, 312 nmHRESIMS *m/z:* 287.0922 $[M-H]^-$ (calcd for $C_{16}H_{15}O_5$, 287.0920); ^1H NMR and ^{13}C NMR (800 and 200 MHz, CD_3OD) [19].
**68- 3-Hydroxy-2,5-dimethylphenyl 2,4-dihydroxy-3,6-dimethylbenzoate (9)**	
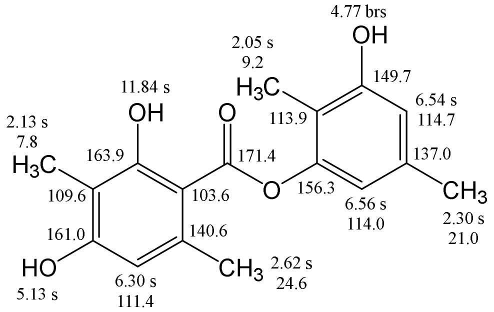	White amorphous powder; UV (MeOH) λ_{max}: 222 (4.43), 276 (4.17), 306 (3.87) nm. IR (KBr) γ_{max}: 3421, 2925, 1652, 1643, 1635, 1271, 1155 cm^{-1}; ESIMS: *m/z* 301 (5, $[M-H]^-$), 163 (100). ESITOFMS: *m/z* 301.1067 ($[M-H]^-$, $C_{17}H_{17}O_5$; Calcd301.1076); ^1H and ^{13}C NMR (400 and 100 MHz, $CDCl_3$ and CD_3OD) [49].
**3-Hydroxy-2,4,5-trimethylphenyl 2,4-dihydroxy-3,6-dimethylbenzoate (10)**	

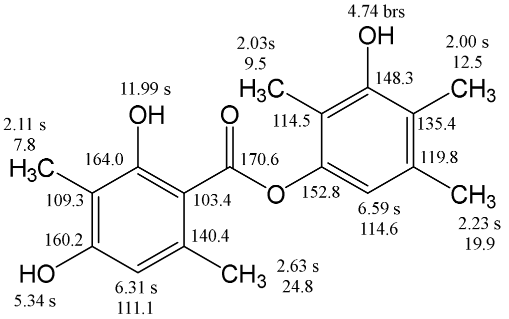	White amorphous powder; UV (MeOH) λ_{max}: 222 (4.43), 274 (4.19), 308 (3.85) nm; IR (KBr) γ_{max}: 3421, 2979, 1652, 1647, 1635, 1627, 1271, 1155 cm^{-1}; ESIMS: *m/z* 315 (2, [M-H]$^-$), 163 (100); ESITOFMS: *m/z* 315.1227 [M-H]$^-$ (calcd for $C_{18}H_{19}O_5$, 315.1232); ^{1}H and ^{13}C NMR (400 and 100 MHz, $CDCl_3$ and CD_3OD) [49].
**Colletotric acid C (11)**	
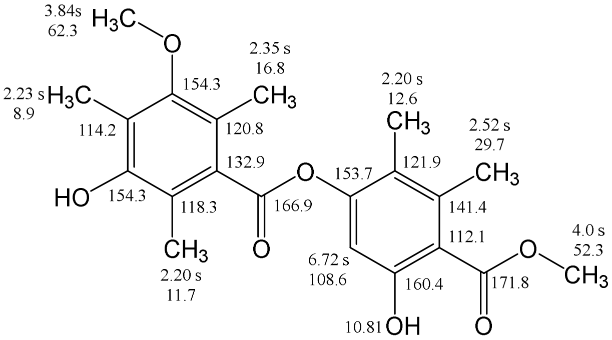	White solid; IR (KBr) γ_{max}: 3346, 2866, 1783, 1672, 1638, 1445, 1311, 1237, 1150, 1065, 981 cm^{-1}; HRESIMS: *m/z* 389.1595 [M+H]$^+$ (calcd for 389.1522, $C_{21}H_{26}O_7$); ^{1}H NMR and ^{13}C NMR (500 and 125 MHz, CD_3OD) [46].
**Agonodepside A (12)**	
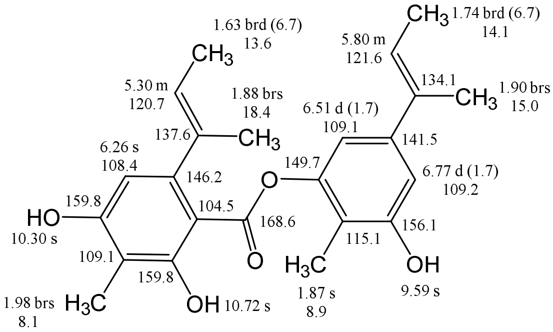	White powder; UV (MeOH) λ_{max} (log ε): 214 (4.57), 275 (4.16), 315 (2.82) nm; IR (KBr) γ_{max}: 3400, 2960, 1725, 1635 cm^{-1}; ESIMS: *m/z* 381 [M-H]$^-$, 203; HRMS: *m/z* 381.1689 [M-H]$^-$ (calcd for $C_{23}H_{25}O_5$, 381.1697); ^{1}H and ^{13}C NMR (500 and 125 MHz, DMSO-d_6) [50].
**Agonodepside B (13)**	
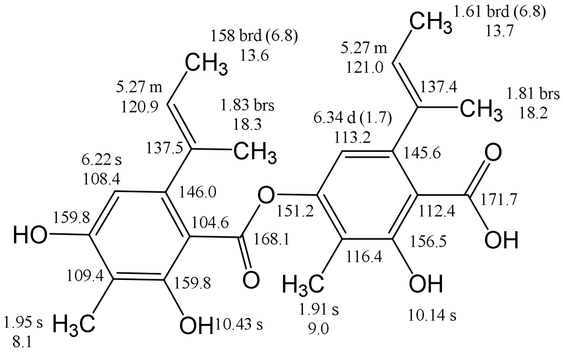	White powder; UV (MeOH) λ_{max} (log ε): 214 (4.57), 275 (4.16), 315 (2.82) nm; IR (KBr) γ_{max}: 3400, 2960, 1725, 1635 cm^{-1}; ESIMS: *m/z* 381 [M-H]$^-$, 203; HRMS: *m/z* 381.1689 (calcd for $C_{23}H_{25}O_5$, 381.1697); ^{1}H and ^{13}C NMR (500 and 125 MHz, DMSO-d_6) [50].
**Guisinol (14)**	

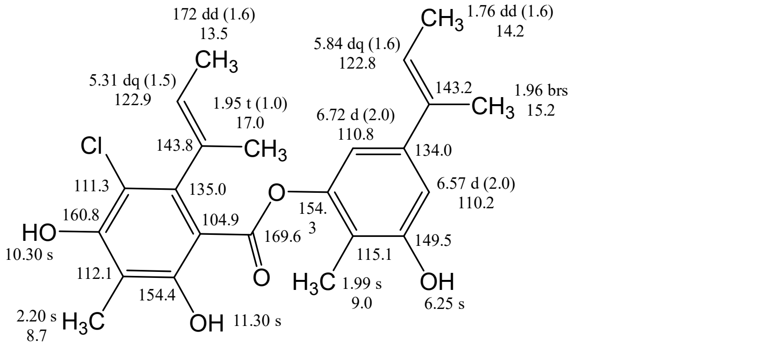	Yellowish oil; UV (MeOH) λ_{max} (log ε): 318 (3.81), 254 (4.09), 216 sh (4.53) nm; HREIMS: *m/z* 416.1396 (calcd for $C_{23}H_{25}O_5Cl$) (11)/418 (4), 239 (100)/241 (38), 211 (14)/213 (5), 178 (89), 163 (21);; ^1H and ^{13}C NMR (400 and 100 MHz, $CDCl_3$) [51].
**Thielavin Z_7 (15)**	
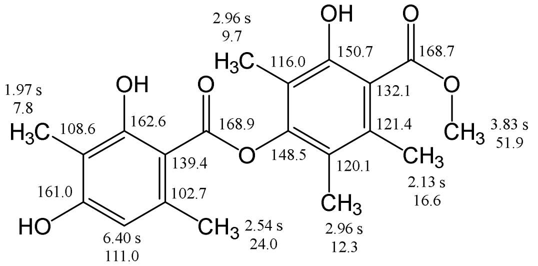	White powder; UV (ACN) λ_{max}: 215.8, 276.1, 308.9 nm; HRESIMS: *m/z* 375.1464 $[M+H]^+$ (calcd. for $C_{20}H_{23}O_7$, 375.1438); ^1H NMR and ^{13}C NMR (500 and 125 MHz, DMSO-d_6) [52].
**Sterenin J (16)**	
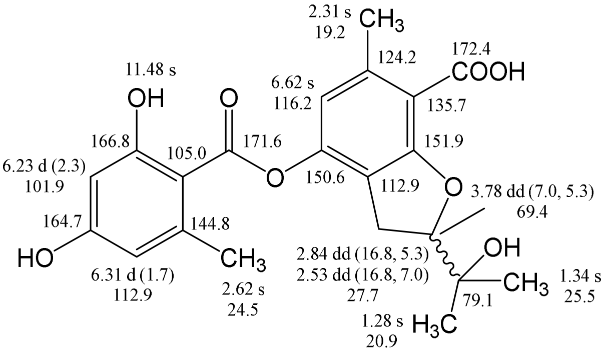	Colorless oil (MeOH); UV (MeOH) λ_{max}: (log ε): 272 (4.15) nm; $[\alpha]^{20}_D$ -11.05 (*c* 0.5, MeOH); IR γ_{max}: 2985, 2925, 2667, 1718, 1654, 1618, 1583, 1449, 1305, 1252, 1202, 1169,1094, 852, 795 cm^{-1}; HRTOFMS: *m/z* 403.1381 $[M+H]^+$ (calcd. for $C_{21}H_{22}O_8H$, 403.1387); ^1H NMR and ^{13}C NMR (500 and 125 MHz, CD_3OD) [53].
**Sterenin E (17)**	
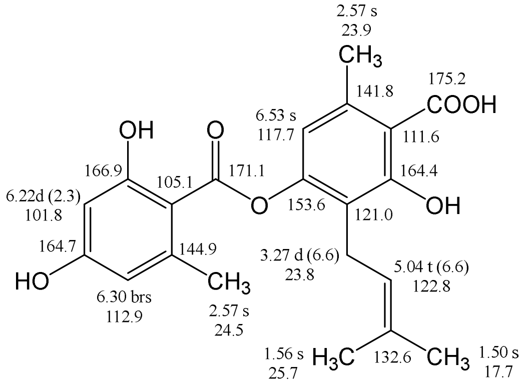	Brown amorphous powder (MeOH); UV (MeOH) λ_{max} (log ε): 266 (4.21) nm; IR γ_{max}: 3360, 3071, 2979, 2938, 1700, 1685, 1641, 1617, 1585, 1441, 1310, 1284, 1157, 1096, 846 cm^{-1}; HRTOFMS: *m/z* 409.1258 $[M+Na]^+$ (calcd. for $C_{21}H_{22}O_7Na$, 409.1258); ^1H NMR and ^{13}C NMR (500 and 125 MHz, CD_3OD) [53].
**Sterenin F (18)**	

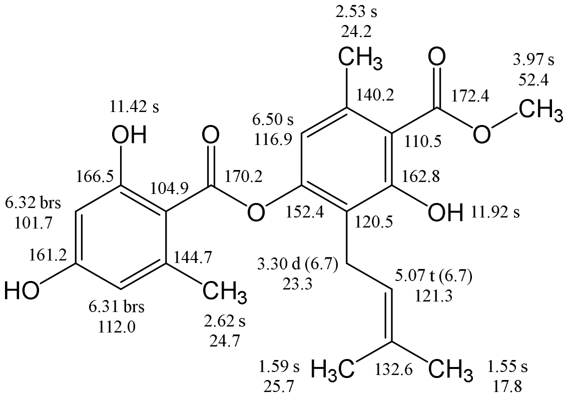	Brown amorphous powder (MeOH); UV (MeOH) λ_{max} (log ε): 265 (4.51) nm; IR γ_{max}: 3370, 2928, 2855, 1663, 1617, 1443, 1403, 1377, 1310, 1250, 1195, 1149, 1095, 806 cm^{-1}; HRTOFMS: *m/z* 423.1419 $[M+Na]^+$ (calcd. for $C_{22}H_{24}O_7Na$, 423.1414; ^1H NMR and ^{13}C NMR (500 and 125 MHz, $CDCl_3$) [53].
**Sterenin G (19)**	
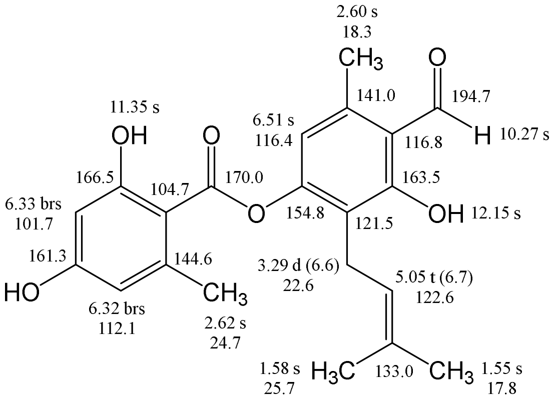	Brown amorphous powder (MeOH); UV (MeOH) λ_{max} (log ε): 260 (4.51) nm; IR γ_{max}: 3375, 2979, 2881, 1719, 1653, 1640, 1617, 1444, 1406, 1377, 1313, 1240, 1197, 1163, 1095, 846 cm^{-1}; HRTOFMS: *m/z* 371.1489 $[M+H]^+$ (calcd. for $C_{21}H_{22}O_6H$, 371.1489); ^1H NMR and ^{13}C NMR (500 and 125 MHz, $CDCl_3$) [53].
**Sterenin H (20)**	
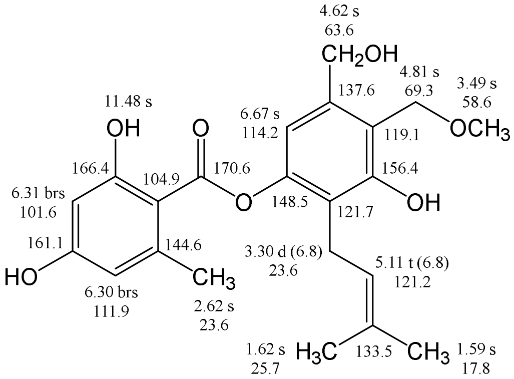	Colorless oil (MeOH); UV (MeOH) λ_{max} (log ε): 270 (4.23) nm; IR γ_{max}: 3306, 2971, 2931, 1658, 1621, 1586, 1445, 1426, 1376, 1312, 1254, 1198, 1166, 1097, 1009, 845, 792, 697 cm^{-1}; HRTOFMS: *m/z* 425.1569 $[M+Na]^+$ (calcd. for $C_{22}H_{26}O_7Na$, 425.1571); ^1H NMR and ^{13}C NMR (500 and 125 MHz, $CDCl_3$) [53].
**Sterenin I (21)**	
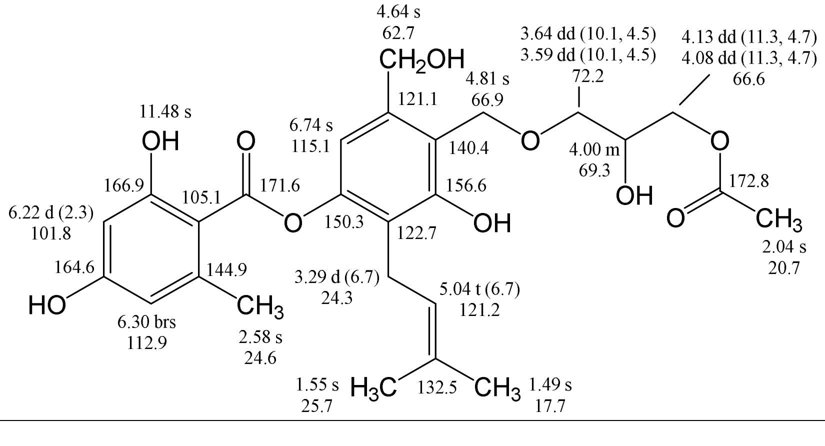	Colorless oil (MeOH); UV (MeOH) λ_{max} (log ε): 270 (1.73) nm; $[\alpha]^{20}_D$ +14.29 (*c* 0.3, MeOH); IR γ_{max}: 3370, 2973, 2951, 1721, 1658, 1621, 1586, 1444, 1426, 1376, 1311, 1255, 1201, 1169, 1098, 1043, 846, 792, 697 cm^{-1}; ^1H NMR and ^{13}C NMR (500 and 125 MHz, CD_3OD); HRTOFMS: *m/z*: 527.1891 $[M+Na]^+$ (calcd. for $C_{26}H_{32}O_{10}Na$, 527.1888).
**MS-3 (22)**	

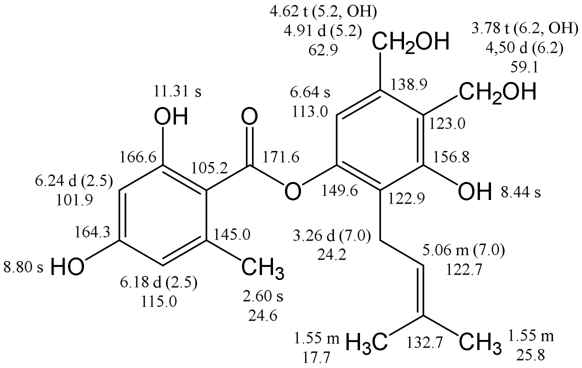	Colorless needle crystals; mp 92 °C; UV (MeOH) λ_{max} (ε): 270 (23280), 305 (86300 nm; IR (KBr) γ_{max}: 3400, 2950, 1655, 1615, 1580, 1495, 1440, 1430, 1375, 1320, 1255, 1190, 1165, 1095, 1060, 1035, 990, 885, 845, 790, 695 cm^{-1}; elemental analysis; 64.91 (C), 6.19 (H), 28.29 (O), $C_{21}H_{24}O_7$; ^{1}H NMR and ^{13}C NMR (100 and 25 MHz, dioxane-*d*8, CD_3OD) [39,55, 56].
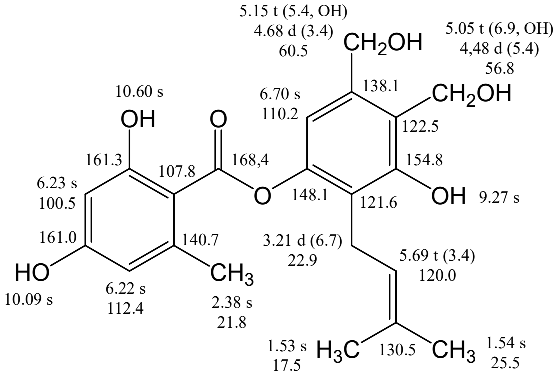<br>^{1}H NMR and ^{13}C NMR (500 and 125 MHz, DMSO-d_6)	HRESIMS: *m/z* 777.3154 [M+H]$^+$ (calcd for $C_{42}H_{48}O_{14}$, 777.3122); 799.2968 [M+Na]$^+$; ^{1}H NMR and ^{13}C NMR (500 and 125 MHz, DMSO-*d*6) [57,58]
4\`-Hydroxy-5\`-methoxy-6\`-(3\`\`-methyl-2\`\`-butenyl)-phenyl-2,4-dihydroxy-6-methyl-benzoate (**23**)	
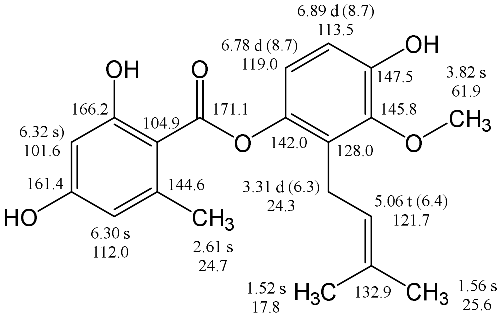	Brown amorphous powder (MeOH); UV (MeOH) λ_{max} (log ε): 202 (4.01), 268 (3.78), 302 (3.37) nm; IR γ_{max}: 3374, 2935, 1660, 1623, 1592, 1482, 1461, 1445, 1259 cm^{-1}; ^{1}H Positive HRTOFMS: *m/z* 381.1309 [M+Na]$^+$ (calcd. for $C_{20}H_{22}O_6Na$, 381.1322); ^{1}H and ^{13}C NMR (500 and 125 MHz, $CDCl_3$) [40].
4\`-Hydroxy-6\`-(3\`\`-methyl-2\`\`-butenyl)-phenyl-2,4-dihydroxy-6-methyl-benzoate (**24**)	
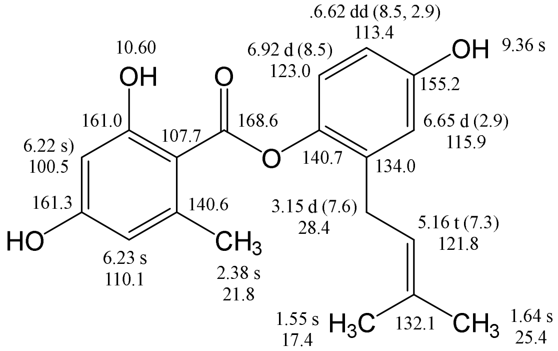	Brown oil; UV (meOH) λ_{max} (log ε): 213 (4.12), 268 (4.10), 302 (3.93) nm; IR (neat) γ_{max}: 3342, 2930, 1679, 1624, 1495, 1445 cm^{-1}; HRTOFMS: *m/z* 329.1384 [M+H]$^+$ (calcd. for $C_{19}H_{21}O_5$, 329.1385) [40].
**Nordivaricatic acid (25)**	

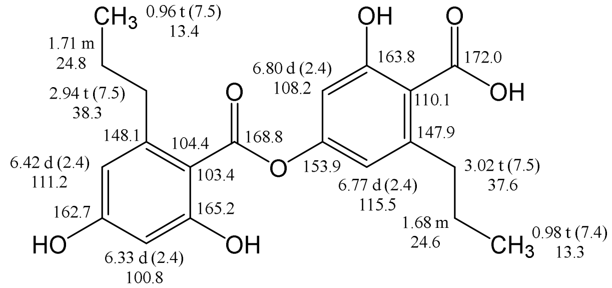	White amorphous powder; ES-IMS: *m/z* 373 [M-H]⁻; ^{1}H NMR and ^{13}C NMR (500 and 125 MHz, acetone-d_6) [25].
**Divarinyl divarate (26)**	
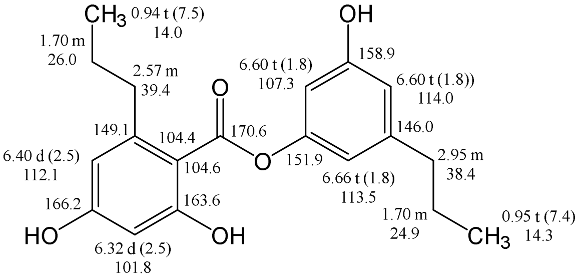	White amorphous powder; ES-IMS: *m/z* 329 [M-H]⁻; ^{1}H NMR and ^{13}C NMR (500 and 125 MHz, acetone-d_6) [25].
**KS-501a (3-Heptyl-5-hydroxyphenyl 2-heptyl-4,6-dihydroxybenzoate) (27)**	
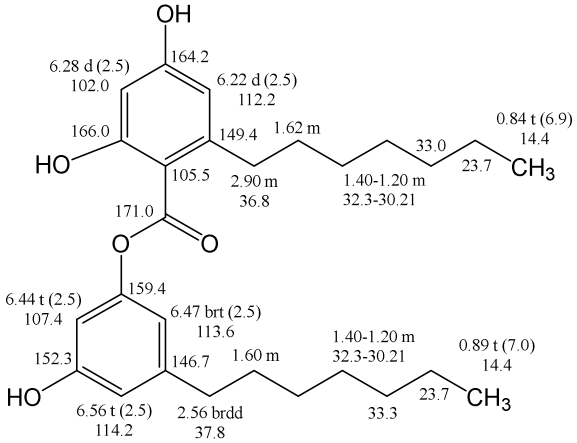	Colorless powder; EIMS: *m/z* 442 (M)⁺, 235, 208, 166, 137, 124; ^{1}H and ^{13}C NMR (400 and 100 MHz, CD_3OD) [41,120].
**KS-501a-2-O-*β*-D-galactopyranose (28)**	
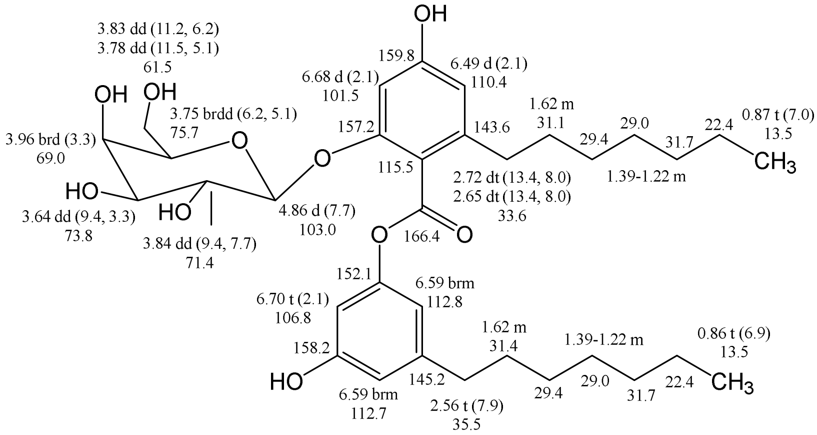	White solid; mp 174-176 °C; $[\alpha]_D^{27}$ -3.3 (*c* 0.10, MeOH); UV (MeOH) λ_{max} (log *ε*): 203 (4.66), 255 (3.87) nm; IR ($CHCl_3$) γ_{max}: 3423, 926, 1728, 1613, 1592, 1465, 1260, 1073 cm^{-1}; HRESITOF: *m/z* 627.3152 [M+Na]⁺ (calcd for $C_{33}H_{48}O_{10}Na$, 627.3145); ^{1}H and ^{13}C NMR (500 and 125 MHz, acetone-d_6) [41].
**KS-501a-2-O-*β*-D-digalactopyranose (29)**	

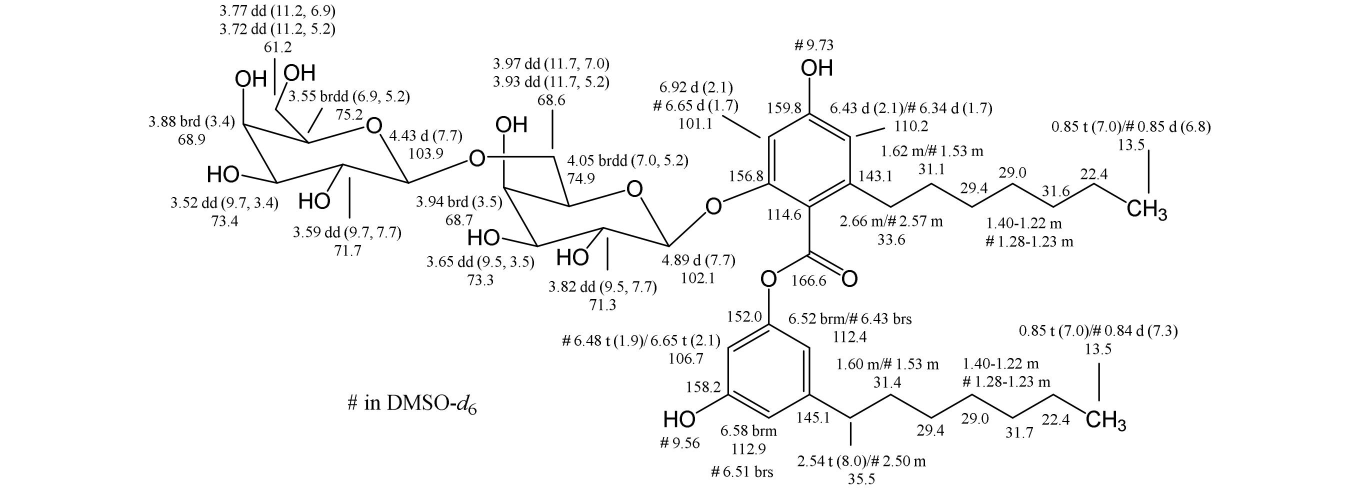

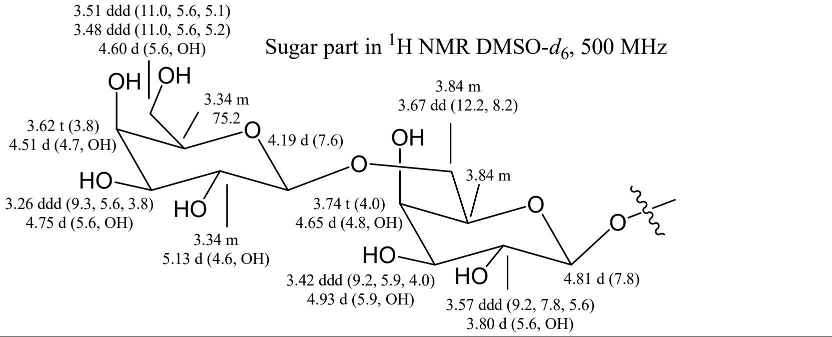

Pale yellow solid; mp 200-202 °C; $[\alpha]_D^{26}$ -10.6 (*c* 0.20, MeOH); UV (MeOH) λ_{max} (log *ε*): 203 (5.10), 253 (4.26) nm; IR ($CHCl_3$) γ_{max}: 3406, 2926, 1725, 1620, 1590, 1465, 1259, 1076 cm^{-1}; HRESITOF: *m/z* 789.3688 $[M + Na]^+$ (calcd for $C_{39}H_{58}O_{15}Na$, 789.3673); ^1H and ^{13}C NMR (500 and 125 MHz, acetone-d_6/D_2O, 95:5) [41].

Arenicolin A (30)

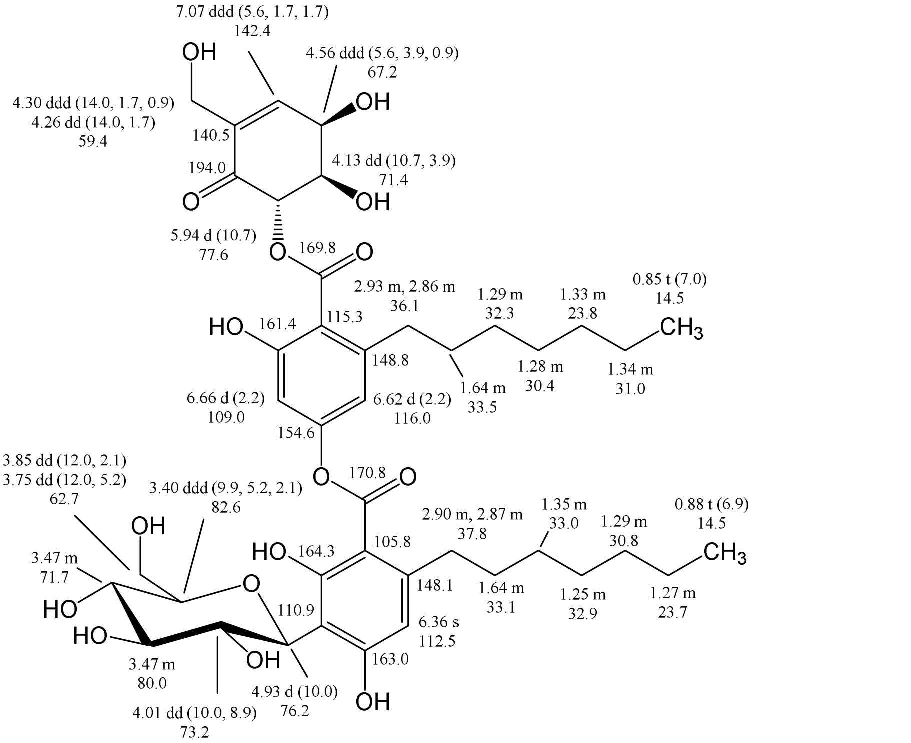

Off-white solid; $[\alpha]_D$ -29 (*c* 0.11, MeOH); UV (MeOH) λ_{max} (log *ε*): 221 (4.3), 272 (4.1), 308 (3.8) nm; ECD (MeOH 4.8 mM) λ_{max} ($\Delta\varepsilon$) 225 (-15), 240 (-56), 262 (+20), 272 (+2.5), 275 (-3.5), 311 (+8.2) nm; HRESIMS *m/z* 803.3495 $[M-H]^-$ (calcd for $C_{41}H_{55}O_{16}$, 803.3490); ^1H NMR and ^{13}C NMR (500 and 125 MHz, CD_3OD) [42]

Arenicolin B (31)

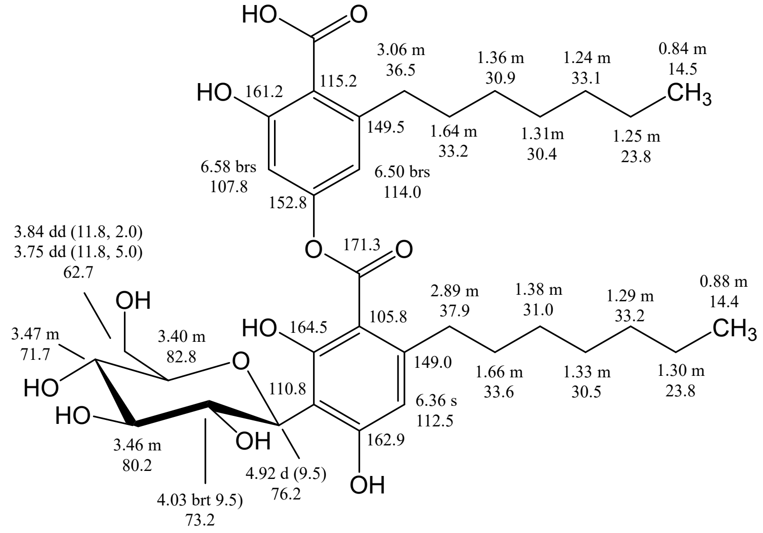	Off-white solid; [α]D +16 (*c* 0.19, MeOH); UV (MeOH) λ_{max} (log *ε*): 220 (4.4), 273 (4.1), 307 (3.9) nm; ECD (MeOH, 7.4 mM) λ_{max} (Δ*ε*) 217 (-16), 227 (-4.6), 250 (-2.7), 311 (+4.3) nm; HRESIMS: *m/z* 647.3064 [M-H]$^-$ (calcd for [$C_{34}H_{48}O_{12}$, 647.3068); ^{1}H NMR and ^{13}C NMR (500 and 125 MHz, CD_3OD) [42].
**72- Aquastatin A (32)**	
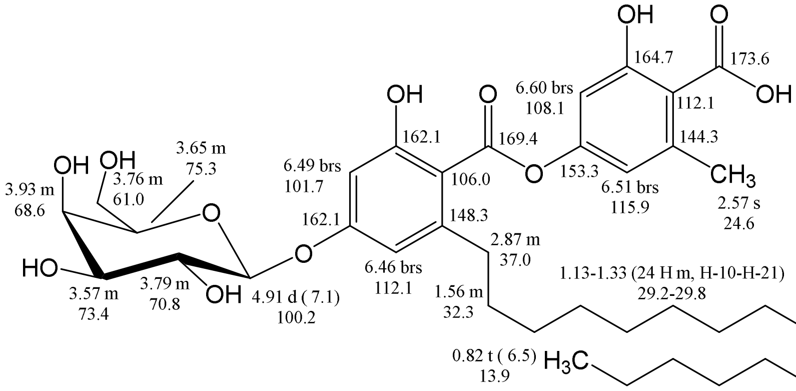	White solid; UV (MeOH) λ_{max} (*ε*): 213 (52933), 265 (19033), 307 (10733) nm; IR ($CHCl_3$) γ_{max}: 2923, 2851, 1665, 1613, 1247, 1070 cm^{-1}; HRFABMS *m/z*: 677.3537 [M+H]$^+$ (calcd for $C_{36}H_{52}O_{12}$, 677.3537) [103]. ^{1}H and ^{13}C NMR (400 and 100 MHz, $CDCl_3$); LRESIMS *m/z* 675 [M-H]$^-$; HRESIMS *m/z*: 675.3350 (M-H)$^-$ (calcd for $C_{36}H_{51}O_{12}$, 675.3381) [60].
**Depsitinuside (33)**	
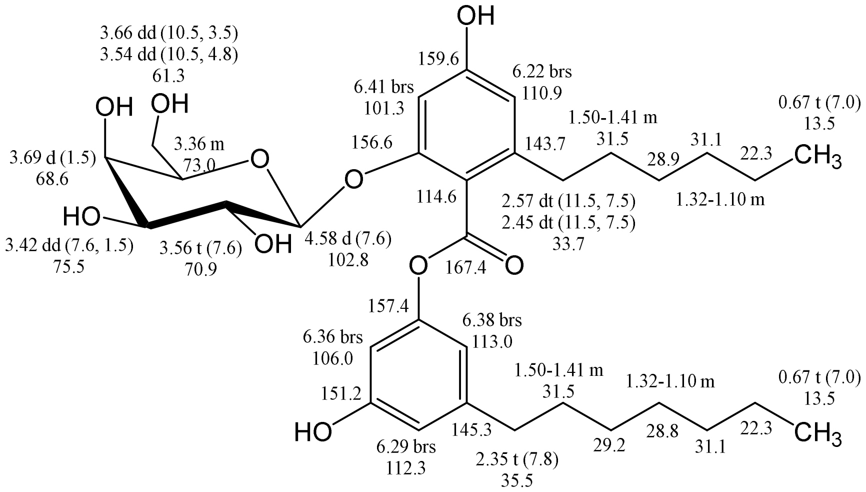	Colorless amorphous powder; IR (KBr) γ_{max}: 3475, 3120, 2920, 1720, 1610, 1560 nm; HRFABMS: *m/z* 589.3009 [M-H]$^-$ (calcd for $C_{32}H_{45}O_{10}$, 589.3012); ^{1}H NMR and ^{13}C NMR (500 and 125 MHz, $CDCl_3$) [62].
**KS-501 (2-(*β*-D-galactofuranosyloxy)-6-heptyl-4-hydroxybenzoic acid 3-heptyl-5-hydroxyphenyl ester) (34)**	

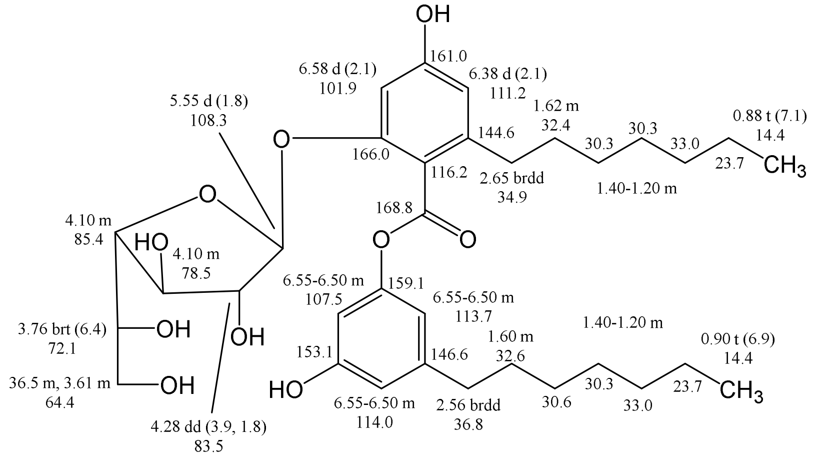

Colorless powder; $[\alpha]_D^{23}$ -53.0 (*c* 0.30, MeOH); UV (MeOH) λ_{max} (ε): 207 (35000), 254 (6600), 278 (5000) nm; IR ($CHCl_3$) γ_{max}: 3400, 1720, 1585, 1064 cm^{-1}; HRFABMS *m*/*z*: 603.3169 $[M+H]^+$ (calcd for $C_{33}H_{47}O_{10}$, 603.3170); ^1H and ^{13}C NMR (400 and 100 MHz, CD_3OD) [63, 120].

KS-502 (2-(*β*-D-galactofuranosyloxy)6-heptyl-4-hydroxybenzoic acid 4-carboxy-3-heptyl-5-hydroxyphenyl ester) (35)

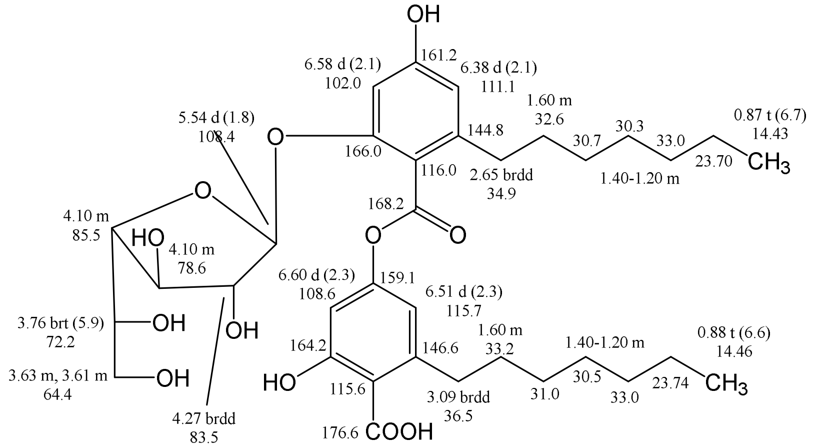

Colorless powder; $[\alpha]_D^{23}$ -45.0 (*c* 0.30, MeOH); UV (MeOH) λ_{max} (ε): 210 (56000), 245 (12000), 290 (6500) nm; IR ($CHCl_3$) γ_{max}: 3450, 1724, 1595, 1057 cm^{-1}; HRF-ABMS *m*/*z*: 647.3083 $[M+H]^+$ (calcd for $C_{34}H_{47}O_{12}$, 647.3068); ^1H and ^{13}C NMR (400 and 100 MHz, CD_3OD) [63,120].

CRM646-A (36)

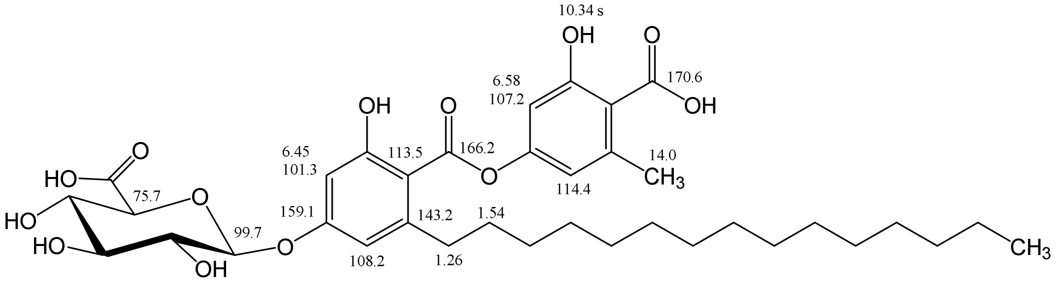

White solid; $[\alpha]_D^{20}$ - 41.5 (c 0.31, MeOH); IR (KBr) γ_{max}: 3397, 2900, 1730, 1666, 1614 cm^{-1}; FABMS: *m*/*z* 689 $[M-H]^-$ ($C_{36}H_{50}O_{13}$); ^1H and ^{13}C NMR (300 and 75 MHz, DMSO-d_6); ^1H NMR (300 MHz, DMSO-d_6): δ_H 10.34 (brs, 1H), 6.57 (s, 1H), 6.49 (m, 3H), 5.70-5.20 (br, 2H), 5.05 (d, 1H, *J* = 7.5 Hz), 3.91 (d, 1H, *J* = 9.0 Hz), 3.46-3.30 (m, 3H), 2.65-2.60 (m, 2H), 2.45 (s, 3 H), 1.60-1.50 (m, 2H), 1.38-1.20 (m, 24H), 0.86 (m, 3H); ^{13}C NMR (75 MHz, $CDCl_3$): δ_C 171.3, 170.2, 166.4, 160.8, 159.4, 157.5, 152.5, 143.4, 140.6, 116.5, 114.3, 113.8, 108.6, 107.4, 101.5, 100.0, 76.0, 75.8, 73.1, 71.6, 33.7, 31.5, 31.1, 29.2, 29.0, 28.9, 22.3, 21.8, 14.1 [43,44].

CRM646-B (37)

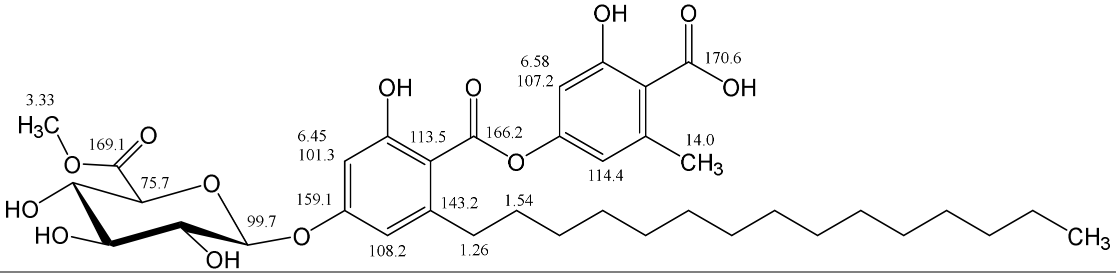	
White solid; $[\alpha]_D^{20}$ - 29.7 (*c* 0.31, CD_3COCD_3); ESIMS: *m/z* 703.3 $[M-H]^-$; IR (KBr) γ_{max}: 3397, 2900, 1730, 1666, 1614 cm^{-1}; FABMS (-): *m/z* 703 $[M-H]^-$, FABMS (-): *m/z* 705 $[M+H]^+$; HRESIMS: *m/z* 727.3300 (calcd for $C_{37}H_{52}O_{13}Na$, 727.3306); ^1H NMR (300 MHz, CD_3COCD_3): δ_H 6.58-6.55 (m, 3H), 6.45 (d, 1H, *J* = 1.8 Hz), 5.28 (d, 1H, *J* = 7.5 Hz), 4.22 (d, 1H, *J* = 9.6 Hz), 3.76-3.73 (m, 4H), 3.74-3.60 (m, 3H), 2.93 (t, 2H, *J* = 7.8 Hz), 2.65 (s, 3H), 1.65-1.60 (m, 2H), 1.35-1.25 (m, 24H), 0.88 (t, 3H, *J* = 6.8 Hz); ^{13}C NMR (75 MHz, CD_3COCD_3): δ_C 170.2, 165.2, 164.4, 162.6, 153.2, 148.5, 115.5, 112.3, 109.1, 108.4, 102.8, 101.1, 77.1, 76.7, 74.3, 72.7, 52.9, 37.2, 33.1, 32.9, 31.0, 30.7, 30.6, 30.5, 30.2, 29.9, 29.7, 29.4, 24.2, 23.6, 14.7 [43,44].	
**Sterenin A (38)**	
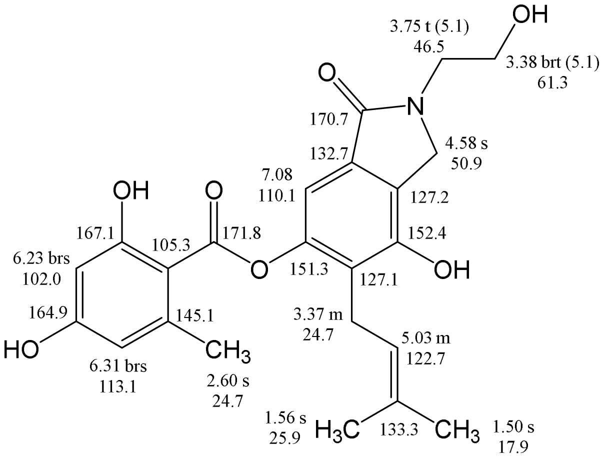	White powder; UV (MeOH) λ_{max} (ε): 212 (58600), 265 (24400), 297 (10900) nm; IR (KBr) γ_{max}: 3357, 3231, 2973, 2931, 1662, 1623, 1602, 1456, 1376, 1311, 1255, 1199, 1164, 1084, 1064, 1035, 990 cm^{-1}; FABMS: *m/z* 450 $[M+Na]^+$; HRFABMS: *m/z* 450.1526 $[M+Na]^+$ (calcd for $C_{23}H_{25}NO_7Na$, 450.1529); ^1H NMR and ^{13}C NMR (500 and 125 MHz, CD_3OD) [64].
**Sterenin B (39)**	
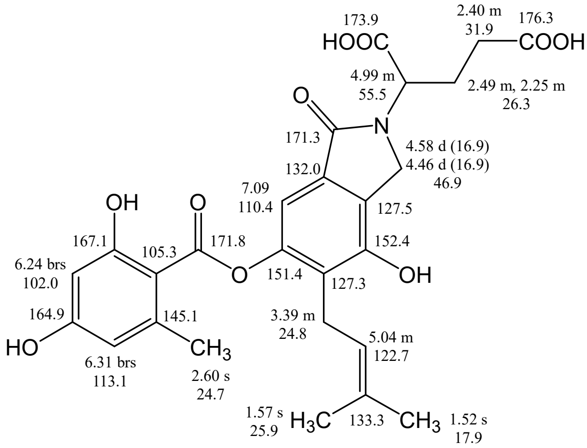	White powder; $[\alpha]D$ -16.3 (*c* 0.05, MeOH); UV (MeOH) λ_{max} (ε): 214 (58100), 265 (26500), 296 (11100) nm; IR (KBr) γ_{max}: 3348, 3264, 2973, 2928, 2858, 1718, 1662, 1624, 1603, 1450, 1413, 1377, 1310, 1254, 1198, 1162, 1087, 1063, 1035, 991 cm^{-1}; FABMS *m/z*: 514 $[M+H]^+$; HRFABMS: *m/z* 514.1697 $[M+H]^+$ (calcd for $C_{26}H_{28}NO_{10}$, 514.1713); ^1H NMR and ^{13}C NMR (500 and 125 MHz, CD_3OD) [64].
**Sterenin C (40)**	

<table>
<tr>
<td>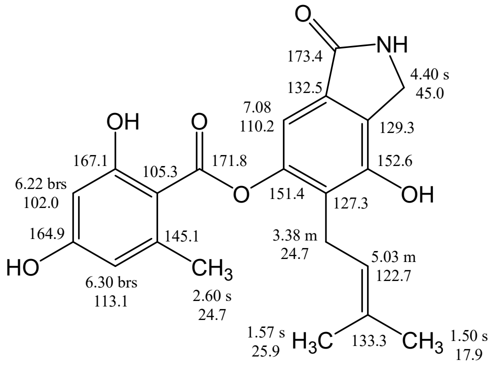
</td>
<td>White powder; UV (MeOH) λ_{max} (ε): 213 (66700), 268 (23000), 297 (12000) nm; IR (KBr) γ_{max}: 3409, 3267, 2974, 2931, 1669, 1653, 1623, 1599, 1504, 1446, 1413, 1374, 1349, 1313, 1255, 1193, 1173, 1161, 1100, 1063, 1038, 998, 989 cm^{-1}; FABMS: *m/z* 406 [M+Na]$^+$; HRFABMS: *m/z* 406.1273 [M+Na]$^+$ (calcd for $C_{21}H_{21}NO_{10}Na$, 406.1267); ^{1}H NMR and ^{13}C NMR (500 and 125 MHz, CD_3OD) [64].</td>
</tr>
<tr>
<td colspan="2">Sterenin D (41)</td>
</tr>
<tr>
<td>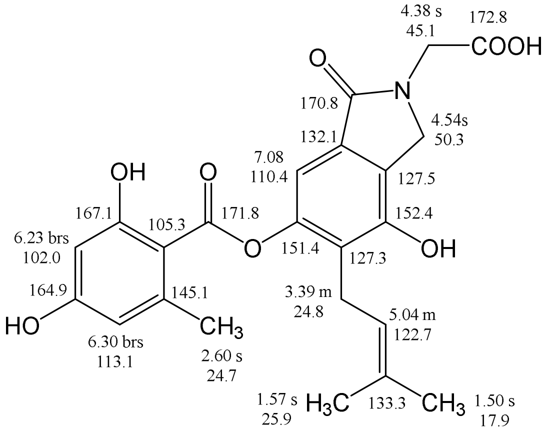
</td>
<td>White powder; UV (MeOH) λ_{max} (ε) 213 (96200), 265 (41300), 297 (17500) nm; IR (KBr) γ_{max}: 3366, 3245, 2973, 2929, 2733, 1662, 1624, 1601, 1458, 1448, 1401, 1377, 1349, 1311, 1254, 1201, 1165, 1103, 1081, 1035, 995, 946 cm^{-1}; FABMS: *m/z* 442 (M+H)$^+$; HRFABMS *m/z*: 442.1495 [M+H]$^+$ (calcd for $C_{23}H_{24}NO_8$, 442.1502); ^{1}H NMR and ^{13}C NMR (500 and 125 MHz, CD_3OD) [64].</td>
</tr>
<tr>
<td colspan="2">Sterenin K (42)</td>
</tr>
<tr>
<td>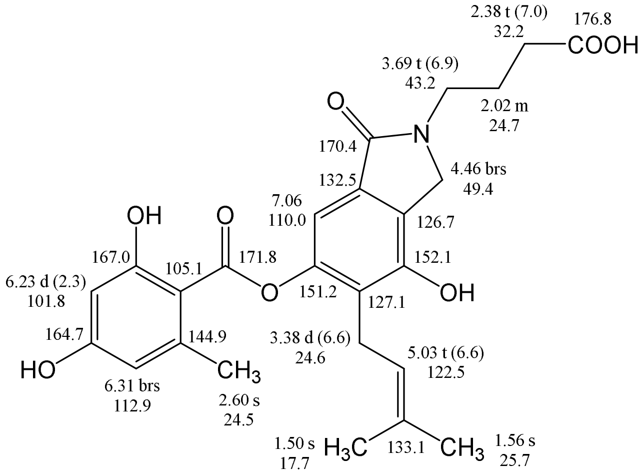
</td>
<td>White powder (MeOH); UV (MeOH) λ_{max} (log ε): 275 (3.41) nm; IR γ_{max}: 3202, 2972, 2930, 1709, 1661, 1601, 1447, 1311, 1254, 1200, 1162, 1104, 1058, 846, 792, 723 cm^{-1}; HRTOFMS: *m/z* 470.1809 [M+H]$^+$ (calcd. for $C_{25}H_{27}NO_8$, 470.1809; ^{1}H NMR and ^{13}C NMR (500 and 125 MHz, CD_3OD) [53].</td>
</tr>
<tr>
<td colspan="2">Sterenin L (43)</td>
</tr>
</table>

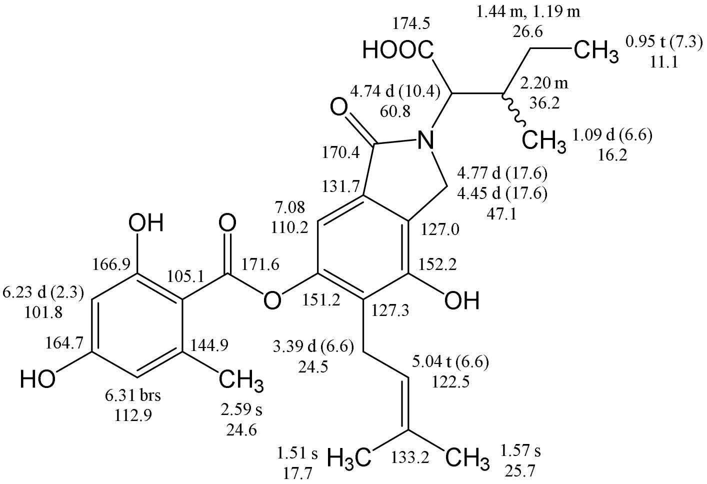	White powder (MeOH); UV (MeOH) λ_{max} (log ε): 263 (2.52) nm; $[\alpha]^{20}_{D}$ -12.05 (*c* 0.2, MeOH); IR γ_{max}: 3193, 2964, 2925, 2874, 1718, 1658, 1623, 1601, 1455, 1310, 1253, 1199, 1160, 1093, 1063, 846, 791 cm^{-1}; HRTOFMS: *m/z* 520.1940 [M+Na]$^+$ (calcd for $C_{27}H_{31}NO_8Na$, 520.1942); ^{1}H NMR and ^{13}C NMR (500 and 125 MHz, CD_3OD) [53].
**Sterenin M (44)**	
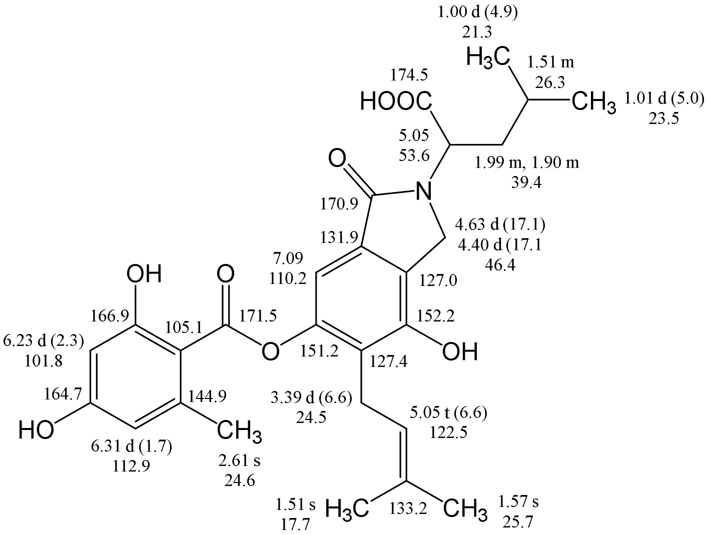	White powder (MeOH); UV (MeOH) λ_{max} (log ε): 264 (3.45) nm; $[\alpha]^{20}_{D}$ -11.33 (*c* 0.3, MeOH); IR γ_{max}: 3368, 2923, 2869, 1711, 1654, 1637, 1599, 1450, 1310, 1253, 1198, 1158, 1093, 1071, 844, 795, 725 cm^{-1}; HRTOFMS: *m/z* 498.2124 [M+H]$^+$ (calcd for $C_{27}H_{31}NO_8$, 498.2122); ^{1}H NMR and ^{13}C NMR (500 and 125 MHz, CD_3OD) [53].
**5- Colletotric acid A (45)**	
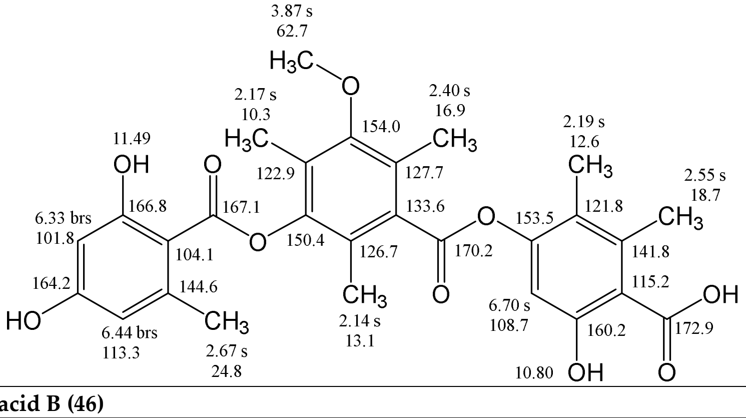	White solid; mp 85-86 °C; EIMS: *m/z* 193 (63.1), 151 (9.1), 124 (66.6), 45 (100); FABMS: *m/z* 525 [M+H]$^+$ (22), 375 (55), 343 (63); HRFABMS: *m/z* 525.1765 [M+H]$^+$ (calcd for $C_{28}H_{28}O_{10}$, 525.1761); ^{1}H and ^{13}C NMR (DMSO-d_6, 400, 125 MHz) [46].
**Colletotric acid B (46)**	

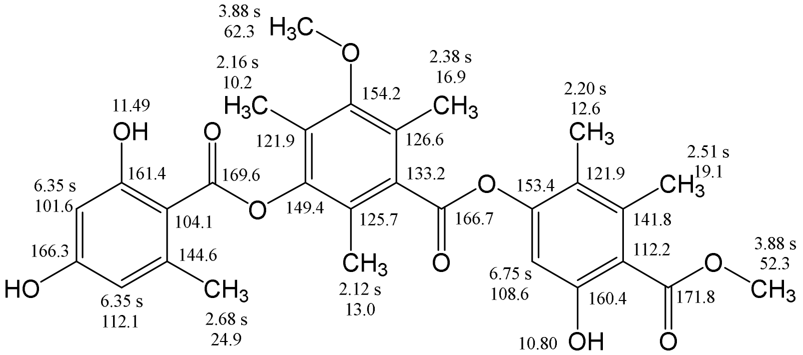

Amorphous powder; IR (KBr) γ_{max}: 3440, 2925, 2860, 1728, 1661, 1450, 1314, 1255, 1173, 1098, 988 cm^{-1}; HRESIMS: *m/z* 537.1774 [M-H]$^-$ (calcd for 37.1766, $C_{29}H_{30}O_{10}$); ^{1}H NMR and ^{13}C NMR (500 and 125 MHz, $CDCl_3$) [46].

PS-990 (47)

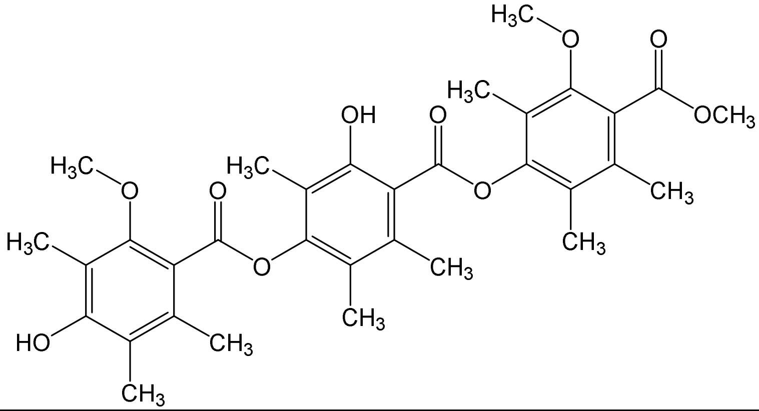

Colorless crystalline; mp 183-185 °C; UV (MeOH) λ_{max}: 215, 257, 323 nm; IR (neat) γ_{max} 3452, 2931, 1734, 1716, 1701, 1662, 1576, 1464, 1313, 1151, 1095, 1074 cm^{-1}; EIMS: *m/z* 179.2230 (calcd for $C_{32}H_{36}O_{10}$) [66].

Thielavin A (48)

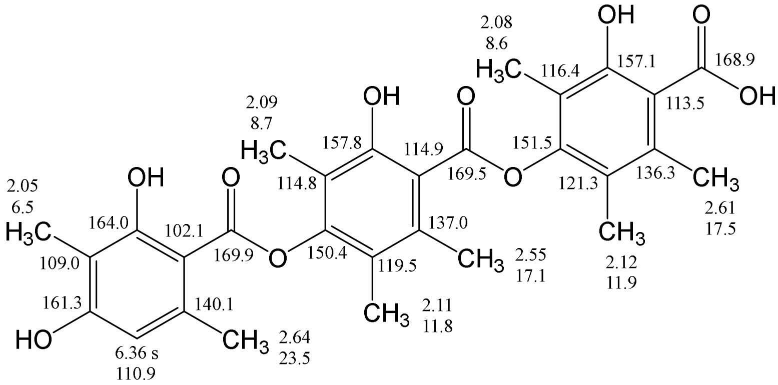

White solid; UV (MeCN) λ_{max}: 272, 315 nm; HRESIMS: *m/z* 537.1765 [M-H]$^-$ (calcd. for $C_{29}H_{29}O_{10}$, 539.1772); ^{1}H and ^{13}C NMR (500 and 125 MHz, $CDCl_3/CD_3OD$, 7:3) [67].

Thielavin B (49)

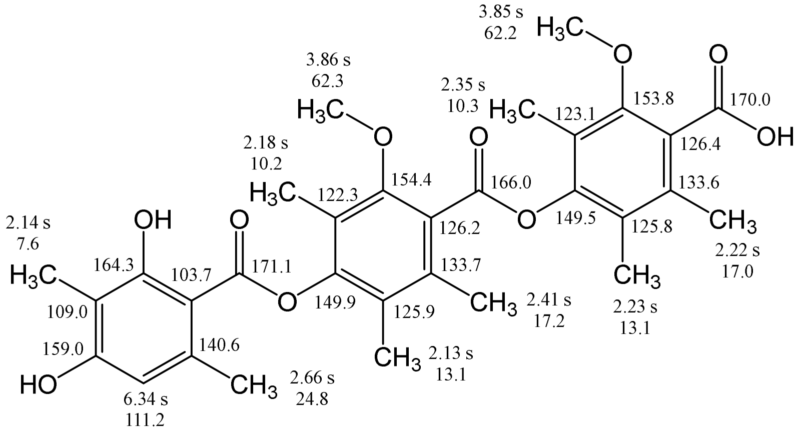

Yellowish powder; ESIMS: *m/z* 589.7 [M+Na]$^+$, 565.7 [M-H]$^-$; ^{1}H and ^{13}C NMR (400 and 100 MHz, $CDCl_3$) [68].

Thielavin B Methyl Ester (50)

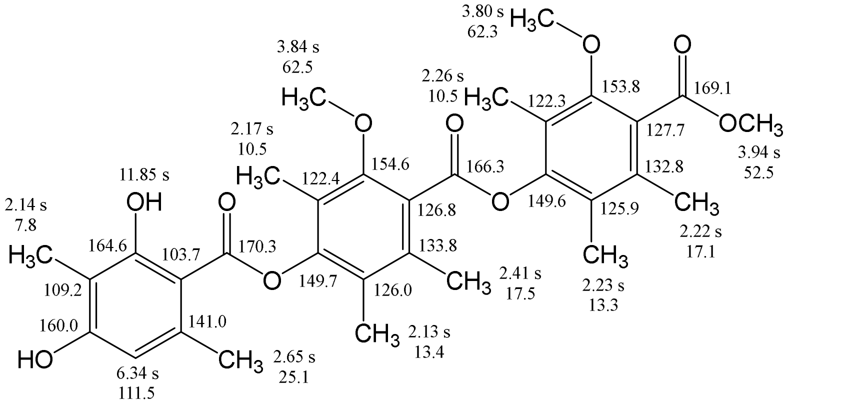	APPIHRMS: *m/z* 581.2378, $[M+H]^+$, (calcd for 581.2387, $C_{32}H_{36}O_{10}$); ^1H and ^{13}C NMR (500 and 125 MHz, $CDCl_3$) [48].
**Thielavin C (51)**	
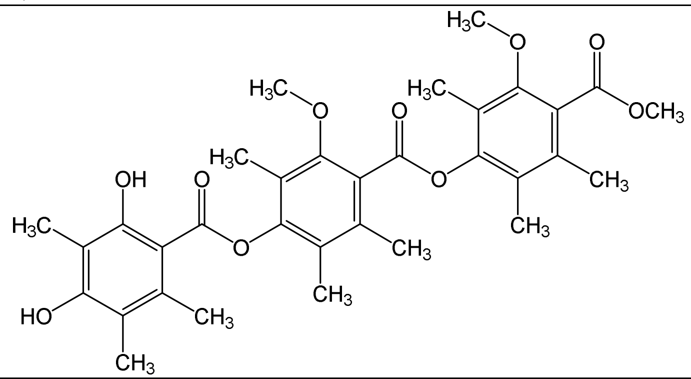	Colorless crystalline; mp 107-109 °C; $C_{32}H_{36}O_{10}$ [69 89].
**Thielavins D (52)***	
Colorless powder; mp 235-238°C; SI-MS: *m/z* 553 ($C_{30}H_{32}O_{10}$); UV (MeOH) λ_{max} (ε): 269.5 (23,110), 303.3 (7,590) nm; UV (dil HCl-MeOH) λ_{max} (ε): 270 (23,050), 302 (46,417) nm [70].	
**Thielavins E (53)***	
Colorless powder; mp 217-220°C; SI-MS: *m/z* 553 ($C_{30}H_{32}O_{10}$); UV (MeOH) λ_{max} (ε): 275.9 (26,236), 305 (5,935) nm; UV (dil HCl-MeOH) λ_{max} (ε): 276.1 (24,679), 314.5 (43,205) nm [70].	
**Thielavin F (54)**	
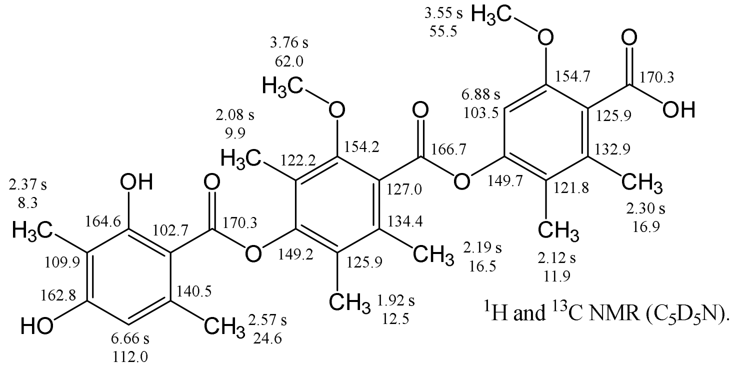	UV (MeOH) λ_{max}: 210, 276, 310 nm; IR (KBr) γ_{max}: 3400, 2937, 1743, 1652, 1604, 1461 cm^{-1}; HRFABMS: *m/z* 551.1928 $[M-H]^-$ (calcd for $C_{30}H_{32}O_{10}$, 551.1917); LRFABMS: *m/z* 553, 535, 389, 357, 343, 193, 179, 165, 151; ^1H and ^{13}C NMR (C_5D_5N) [121].

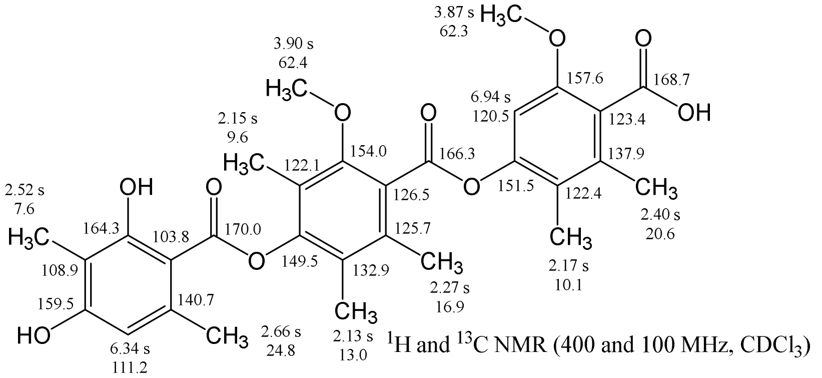	Pale yellow amorphous powder; ESIMS: *m/z* 575.7 [M+Na]$^+$, 551.7 [M-H]$^-$; ^{1}H and ^{13}C NMR (400 and 100 MHz, CDCl$_3$) [68].
**Thielavin G (55)**	
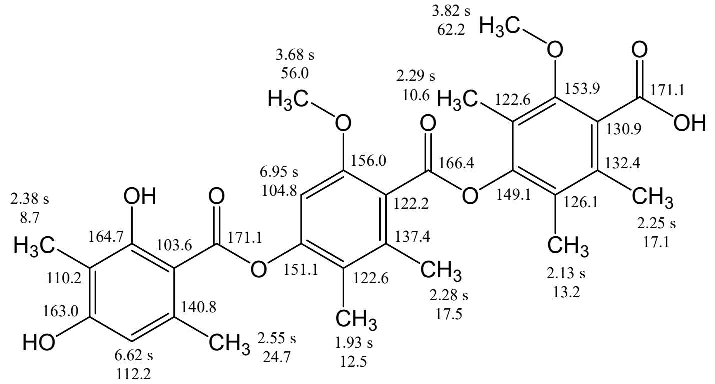	UV (MeOH) λ_{max} 210, 276, 310 nm; IR (KBr) γ_{max}: 3400, 2941, 1743, 1651, 1419, 1269 cm^{-1}; HRFABMS: *m/z* 551.1912 [M-H]$^-$ (calcd for $C_{30}H_{32}O_{10}$, 551.1918); LRFABMS: *m/z* 553, 535, 461, 389, 343, 329, 193, 179, 165; ^{1}H and ^{13}C NMR (C_5D_5N) [121].
**Thielavin H (56)**	
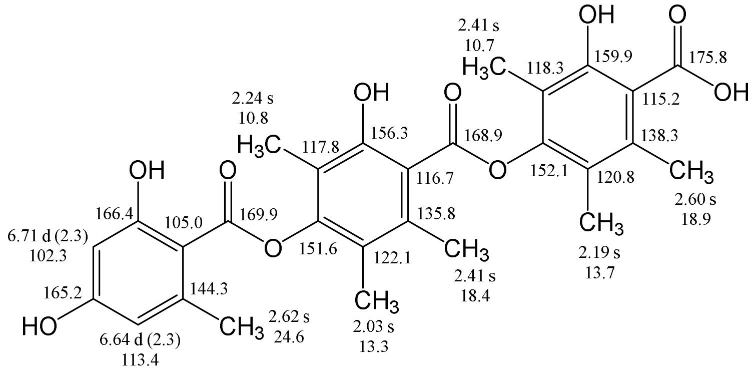	UV (MeOH) λ_{max}: 215, 267, 308 nm; IR (KBr) γ_{max}: 3400, 2929, 1660, 1446, 1315, 1255 cm^{-1}; HRFABMS: *m/z* 523.1601 [M-H]$^-$ (calcd for $C_{28}H_{30}O_{10}$, 523.1604); LRFABMS: *m/z* 525, 375, 329, 179, 151; ^{1}H and ^{13}C NMR (C_5D_5N) [121].
**Thielavin I (57)**	
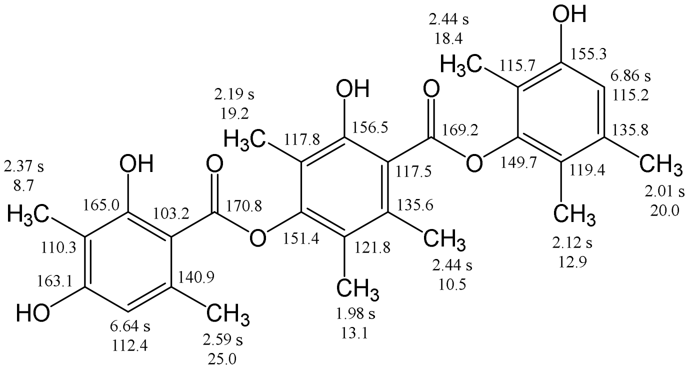	UV (MeOH) λ_{max}: 218, 275, 310 nm; IR (KBr) γ_{max}: 3398, 2931, 1660, 1622, 1417, 1307 cm^{-1}; HRFABMS: *m/z* 493.1862 [M-H]$^-$ (calcd for $C_{28}H_{30}O_8$, 493.1862); LRFABMS: *m/z* 495, 343, 331, 231, 179, 165; ^{1}H and ^{13}C NMR (C_5D_5N) [121].
**Thielavin J (58)**	

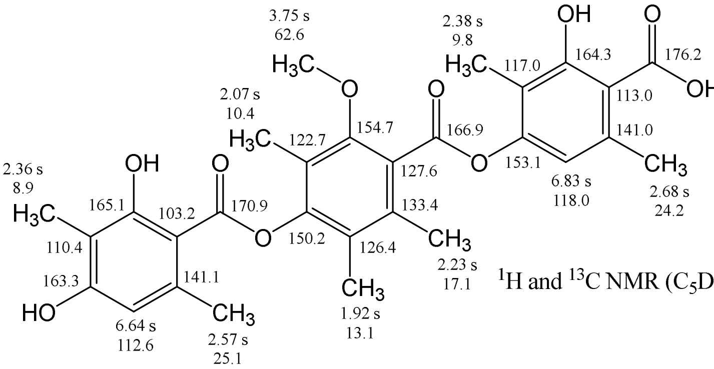 ^1H and ^{13}C NMR (C_5D_5N)	UV (MeOH) λ_{max}: 214, 250, 275, 306 nm; IR (KBr) γ_{max}: 3400, 2933, 1654, 1622, 1456, 1265 cm^{-1}; HRFABMS: *m/z* 537.1750 [M-H]$^-$ (calcd for $C_{29}H_{30}O_{10}$, 537.1760); LRFABMS: *m/z* 539, 375, 357, 343, 193, 165; ^1H and ^{13}C NMR (C_5D_5N) [121].
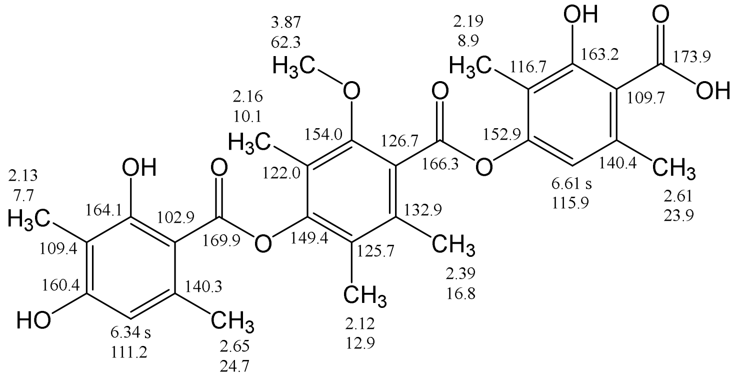 ^1H and ^{13}C NMR (500 and 125 MHz, $CDCl_3/CD_3OD$, 7:3)	White solid; UV (MeOH) λ_{max}: 272, 308; IR γ_{max}: 3408, 2942, 1725, 1653, 1605 cm^{-1}; HRESIMS: *m/z* 539.19026 [M+H]$^-$ (calcd. for $C_{29}H_{31}O_{10}$, 539.1911); ^1H and ^{13}C NMR (500 and 125 MHz, $CDCl_3/CD_3OD$, 7:3) [67].
**Thielavin K (59)**	
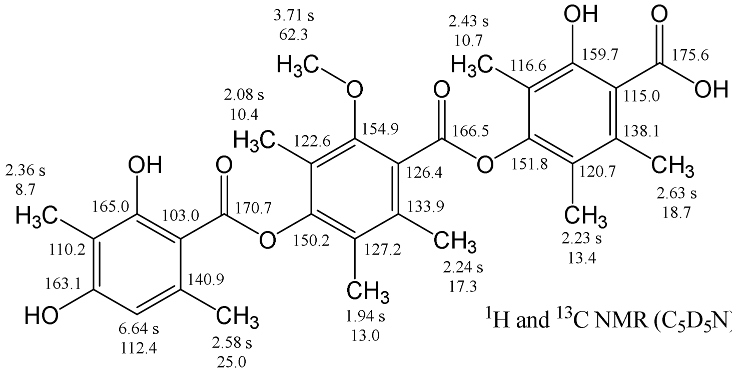 ^1H and ^{13}C NMR (C_5D_5N)	UV (MeOH) λ_{max}: 215, 250, 274, 307 nm; IR (KBr) γ_{max}: 3400, 2935, 1726, 1620, 1417, 1265 cm^{-1}; HRFABM: *m/z* 551.1901 [M-H]$^-$ (calcd for $C_{29}H_{30}O_{10}$, 551.1918); LRFABMS: *m/z* 553, 509, 389, 357, 193, 165; ^1H and ^{13}C NMR (C_5D_5N) [121].
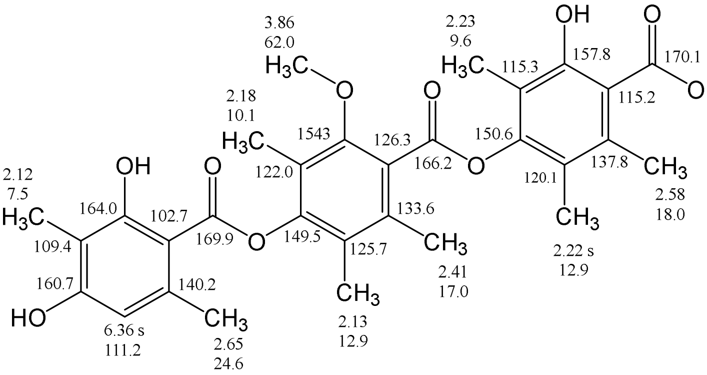 ^1H and ^{13}C NMR (500 and 125 MHz, $CDCl_3/CD_3OD$, 7:3)	White solid; UV (MeOH) λ_{max}: 271, 310 nm; IR (Reflectance) γ_{max}: 3405, 2932, 1728, 1653, 1618 cm^{-1}; HRESIMS: *m/z* 553.2060 [M+H]$^+$ (calcd. for $C_{29}H_{31}O_{10}$, 553.2068); ^1H and ^{13}C NMR (500 and 125 MHz, $CDCl_3/CD_3OD$, 7:3) [67].
**Thielavin L (60)**	

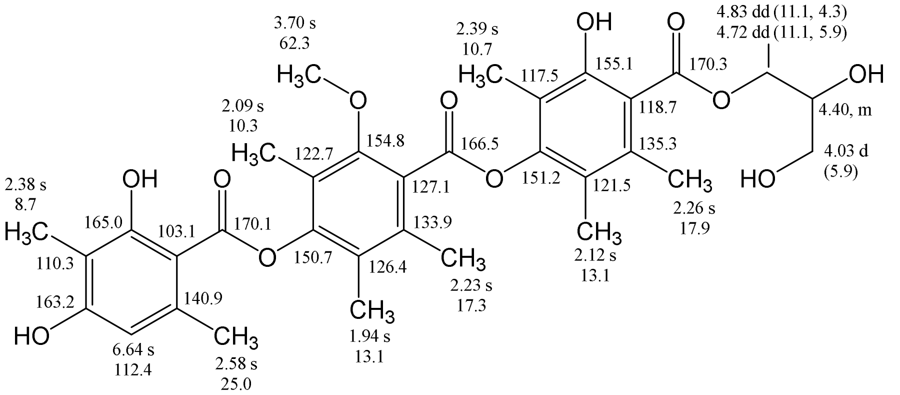	UV (MeOH) λ_{max}: 214, 275, 310 nm; IR (KBr) γ_{max}: 3398, 2933, 1733, 1654, 1606, 1458 cm^{-1}; HRFABMS: *m*: 625.2280 [M-H]$^-$ (calcd for $C_{33}H_{38}O_{12}$, 625.2285); LRFABMS: *m/z* 627, 535, 463, 357, 193, 165; ^{1}H and ^{13}C NMR (C_5D_5N) [121].
**Thielavin M (61)**	
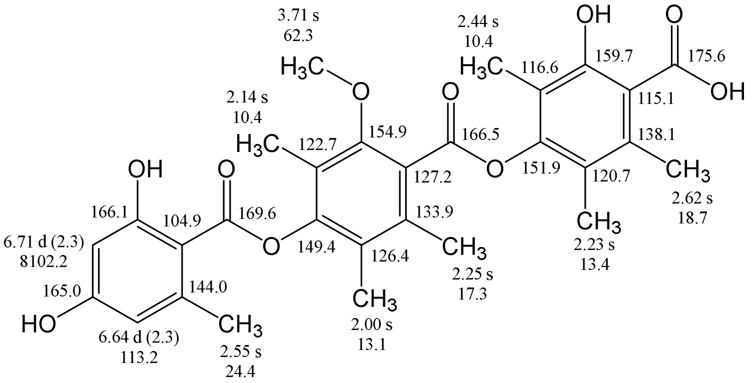	UV (MeOH) λ_{max}: 214, 275, 310 nm; IR (KBr) γ_{max}: 3400, 2929, 1733, 1652, 1624, 1458 cm^{-1}; HRFABMS: *m/z* 537.1766 [M-H]$^+$ (calcd for $C_{29}H_{30}O_{10}$, 537.1760); LRFAB-MS: *m/z* 539, 389, 343, 193, 151; ^{1}H and ^{13}C NMR (C_5D_5N) [121].
**Thielavin N (62)**	
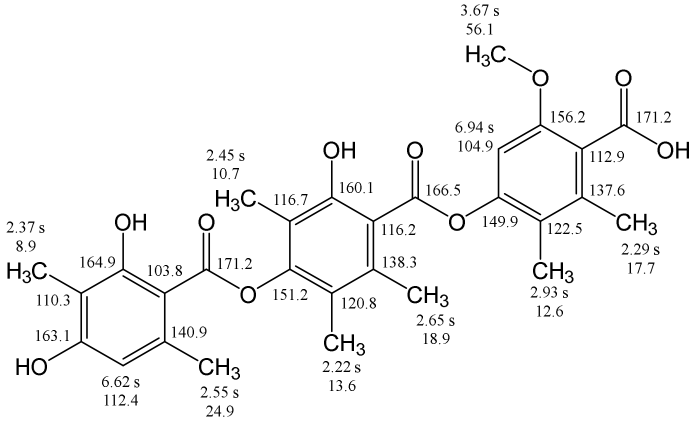	UV (MeOH) λ_{max}: 214, 253, 275, 318 nm; IR (KBr) γ_{max}: 3421, 2933, 1743, 1654, 1604, 1419 cm^{-1}; HRFABMS: *m/z* 537.1773 [M-H]$^+$ (calcd for $C_{29}H_{30}O_{10}$, 537.1761); LRFAB-MS: *m/z* 539, 461, 318, 179, 165; ^{1}H and ^{13}C NMR (C_5D_5N) [121].
**Thielavin O (63)**	
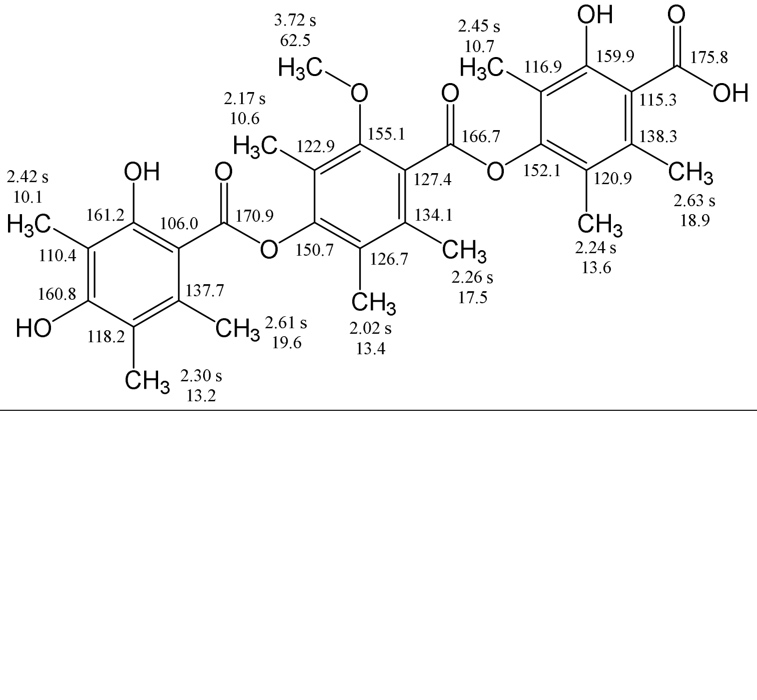	UV (MeOH) λ_{max}: 214, 252, 275, 318 nm; IR (KBr) γ_{max}: 3421, 2929, 1733, 1652, 1651, 1610, 1458 cm^{-1}; HRF-ABMS: *m/z* 565.2078 [M-H]$^+$ (calcd for $C_{31}H_{34}O_{10}$, 565.2073); LRFABMS: *m/z* 539, 389, 343, 193, 151; ^{1}H and ^{13}C NMR (C_5D_5N) [121].

<table>
<tr><td colspan="2">Thielavin P (64)</td></tr>
<tr><td>
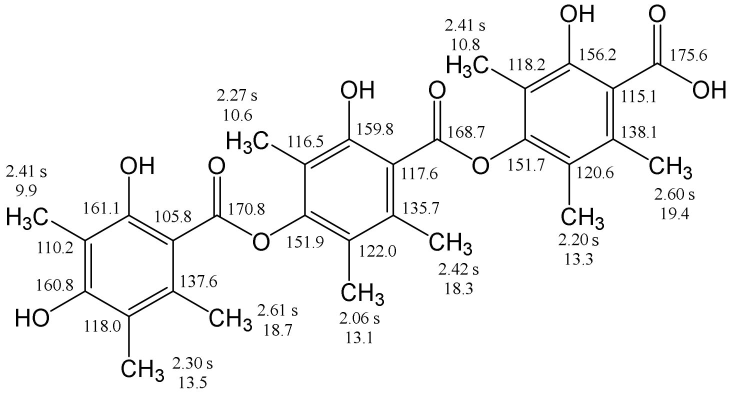

</td><td>UV (MeOH) λmax: 216, 258, 273, 318 nm; IR (KBr) γmax: 3400, 2927, 1652, 1612, 1456, 1419 cm^{-1}; HRFABMS: <i>m/z</i> 551.1929 [M-H]$^{-}$ (calcd for $C_{30}H_{32}O_{10}$, 551.1918); LRFABMS: m/z 553, 344, 193, 179; ^{1}H and ^{13}C NMR (C_5D_5N) [121].</td></tr>
<tr><td colspan="2">Thielavin Q (65)</td></tr>
<tr><td>
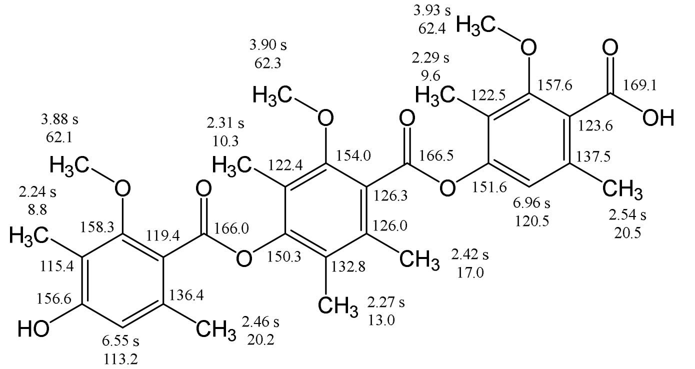

</td><td>White amorphous powder; [α]$^{15}_{D}$ +6.3 (c 0.1, MeOH); UV (MeOH) λmax (log ε): 288 (3.31) nm; IR (KBr) γmax: 3426, 1734, 1666, 1402, 1376, 1172, 1075 cm^{-1}; HRESIMS: <i>m/z</i> 565.2075 [M-H]$^{-}$ (calcd for $C_{31}H_{34}O_{10}$, 565.2074); ^{1}H and ^{13}C NMR (900 and 225 $CDCl_3$) [68].</td></tr>
<tr><td colspan="2">Thielavin S (66)</td></tr>
<tr><td>
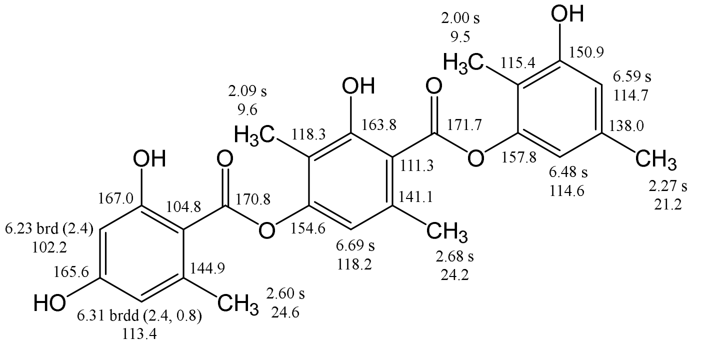

</td><td>White powder; UV (MeOH) λmax: 222, 272, 313 nm; HRESIMS: m/z 451.1394 [M-H]$^{-}$ (calcd for $C_{25}H_{23}O_8$, 451.1393); ^{1}H NMR and ^{13}C NMR (500 and 125 MHz, CD_3OD) [19].</td></tr>
<tr><td colspan="2">Thielavin T (67)</td></tr>
<tr><td>
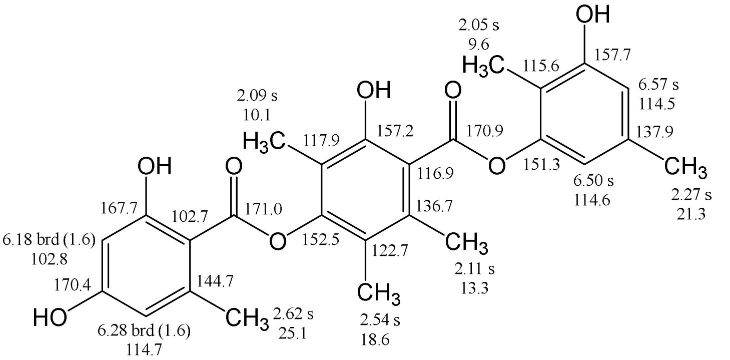

</td><td>White powder; UV (MeOH) λmax: 225, 272, 308 nm; HRESIMS: m/z 465.1544 [M-H]$^{-}$ (calcd for $C_{26}H_{25}O_8$, 465.1549); ^{1}H NMR and ^{13}C NMR (400 and 100 MHz, CD_3OD) [19].</td></tr>
<tr><td colspan="2">Thielavin U (68)</td></tr>
</table>

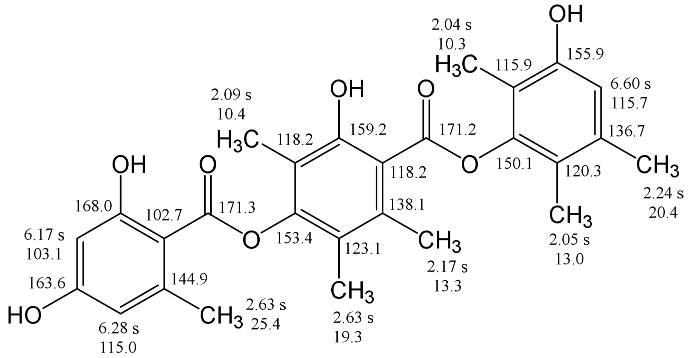

White powder; UV (MeOH) λ_{max}: 218, 272, 310 nm; RESIMS: *m/z* 479.1707 $[M-H]^-$ (calcd for $C_{27}H_{27}O_8$, 479.1706); ^1H NMR and ^{13}C NMR (800 and 200 MHz, CD_3OD) [19].

Thielavin V (69)

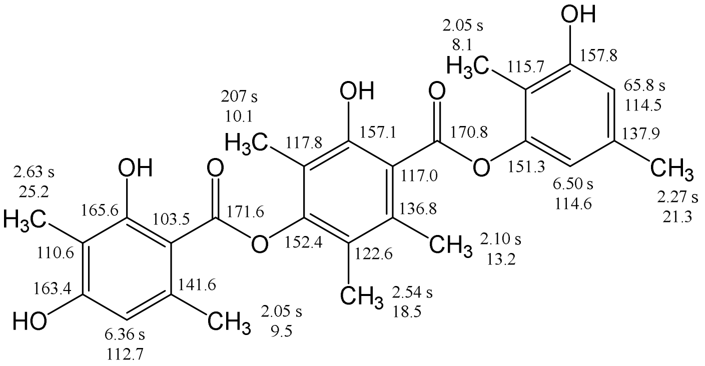

White powder; UV (MeOH) λ_{max}: 224, 276, 313 nm; HRESIMS: *m/z* 479.1708 $[M-H]^-$ (calcd for $C_{27}H_{27}O_8$, 479.1706); ^1H NMR and ^{13}C NMR (400 and 100 MHz, CD_3OD) [19].

Thielavin W (70)

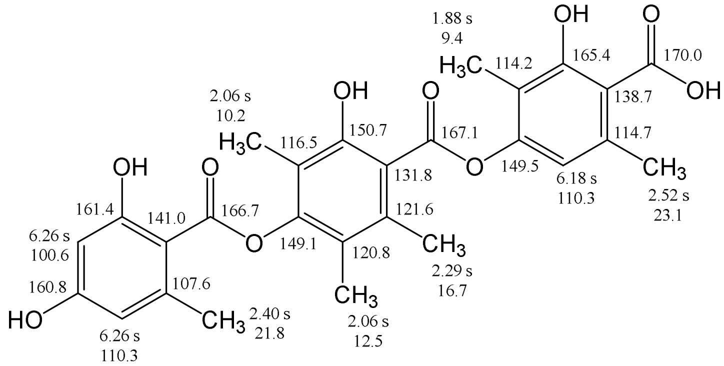

White powder, UV (CAN) λ_{max}: 218.1, 267.8, 304.2 nm; HRESIMS: *m/z* 511.1604 $[M+H]^+$ (calcd. for $C_{27}H_{27}O_{10}$, 511.1599); ^1H NMR and ^{13}C NMR (500 and 125 MHz, DMSO-d_6) [52].

Thielavin X (71)

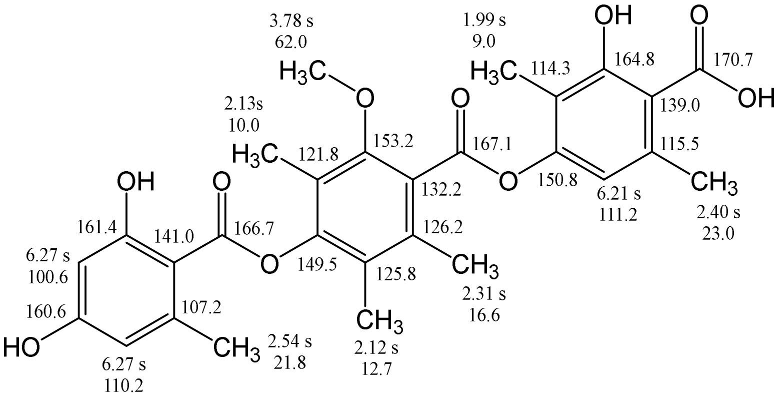

White powder, UV (CAN) λ_{max}: 218.1, 267.8, 306.5 nm; HRESIMS: *m/z* 525.1727 $[M+H]^+$ (calcd for $C_{28}H_{29}O_{10}$, 525.1755); ^1H NMR and ^{13}C NMR (500 and 125 MHz, DMSO-d_6) [52].

Thielavin Y (72)

<table>
<tr><td>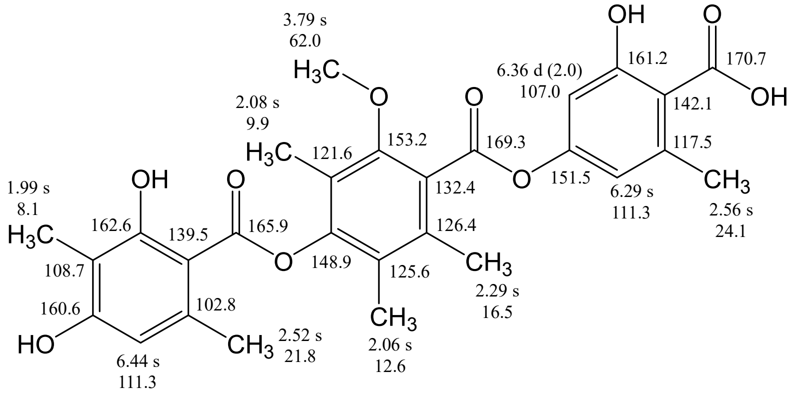
</td><td>White powder; UV (CAN) λ_{max}: 218.1, 276.1, 304.7 nm; HRESIMS: *m/z* 525.1765 [M+H]$^+$ (calcd. for $C_{28}H_{29}O_{10}$, 525.1755); ^{1}H NMR and ^{13}C NMR (500 and 125 MHz, DMSO-d_6) [52].</td></tr>
<tr><td colspan="2">Thielavin Z (73)</td></tr>
<tr><td>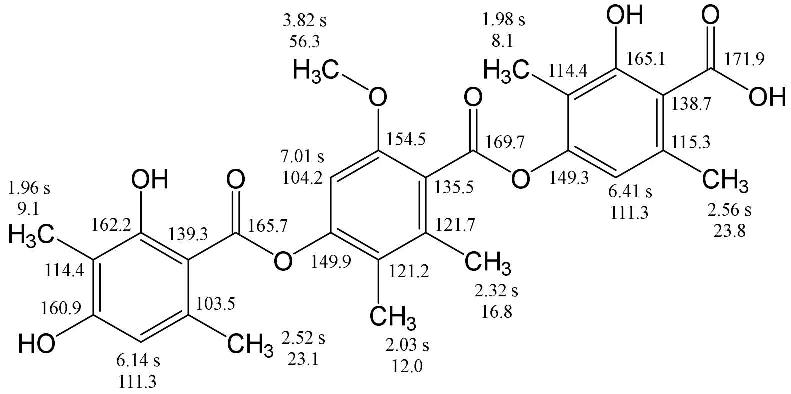
</td><td>White powder; UV (CAN) λ_{max}: 218.1, 276.1, 306.5 nm; HRESIMS: *m/z* 525.1757 [M+H]$^+$ (calcd for $C_{28}H_{29}O_{10}$, 525.1755); ^{1}H NMR and ^{13}C NMR (500 and 125 MHz, DMSO-d_6) [52].</td></tr>
<tr><td colspan="2">Thielavin Z_1 (74)</td></tr>
<tr><td>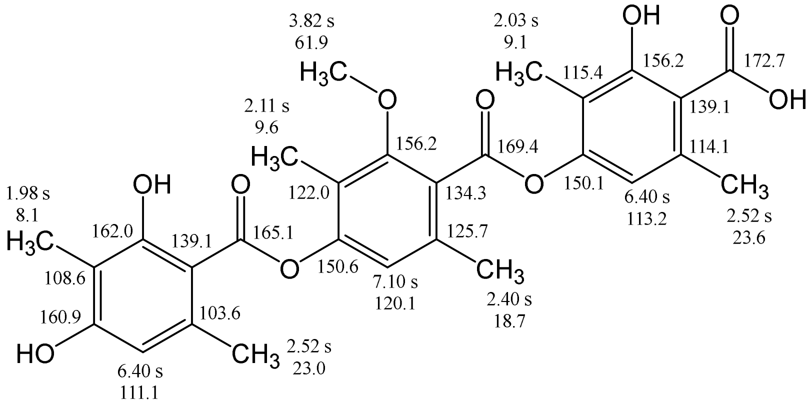
</td><td>White powder; UV (CAN) λ_{max}: 218.1, 276.1, 306.9 nm; ^{1}H NMR and ^{13}C NMR (500 and 125 MHz, DMSO-d_6); HRESIMS: *m/z* 525.1747 [M+H]$^+$ (calcd for $C_{28}H_{29}O_{10}$, 525.1755) [52].</td></tr>
<tr><td colspan="2">Thielavin Z_2 (75)</td></tr>
<tr><td>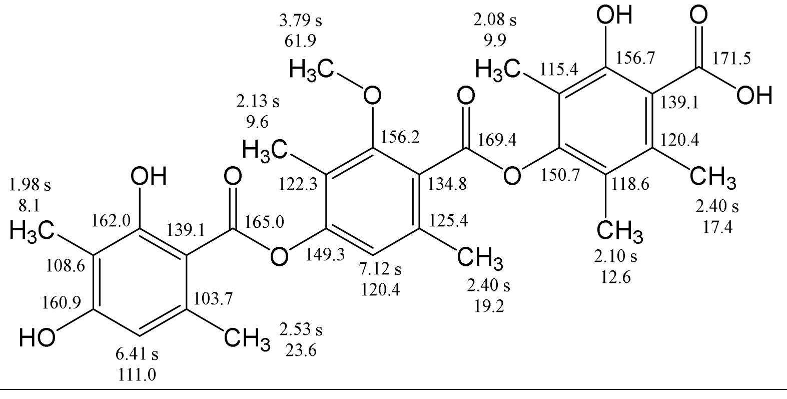
</td><td>White powder, UV ACN λ_{max} 218.1, 267.8, 305.2 nm; ^{1}H NMR and ^{13}C NMR (500 and 125 MHz, DMSO-d_6); HRESIMS *m/z*: 539.1916 [M+H]$^+$ (calcd. for $C_{29}H_{31}O_{10}$ 539.1912); ISCID: 507.1, 375.2, 343.1, 179.1, 165.1 [52].</td></tr>
</table>

Thielavin Z3 (76)

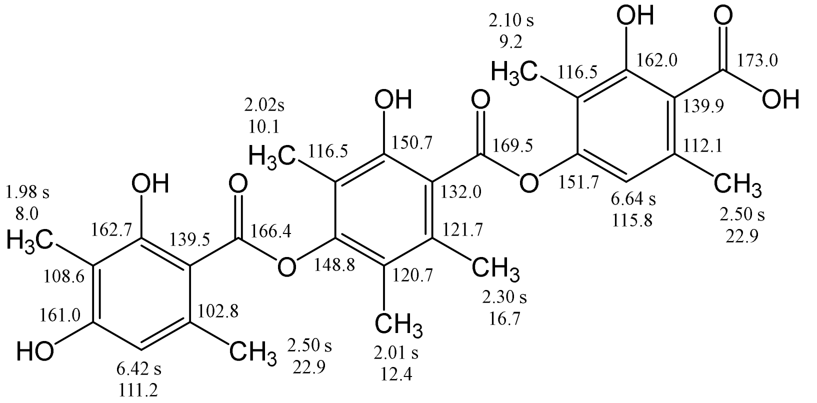

White powder; UV (CAN) λ_{max}: 214.6, 276.1, 309.1 nm; HRESIMS: *m/z* 525.1758 $[M+H]^+$ (calcd for $C_{28}H_{29}O_{10}$, 525.1755); ^1H NMR and ^{13}C NMR (500 and 125 MHz, DMSO-d_6 [52].

Thielavin Z4 (77)

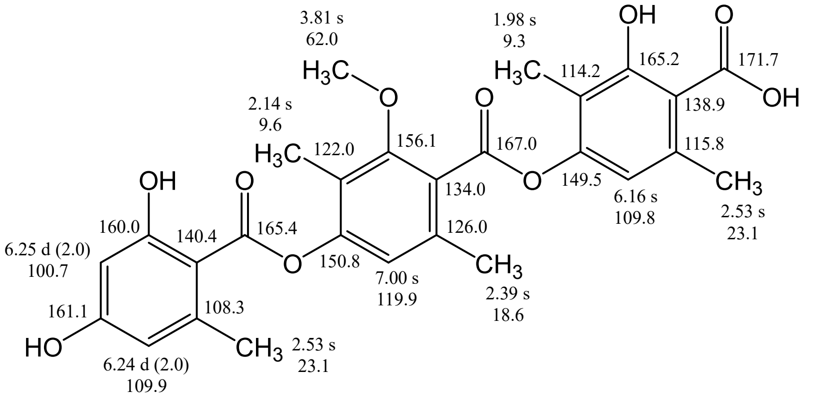

White powde; UV (CAN) λ_{max}: 218.1, 267.8, 305.2 nm; HRESIMS: *m/z* 511.1564 $[M+H]^+$ (calcd for $C_{27}H_{27}O_{10}$, 511.1599); ^1H NMR and ^{13}C NMR (500 and 125 MHz, DMSO-d_6) [52].

Thielavin Z5 (78)

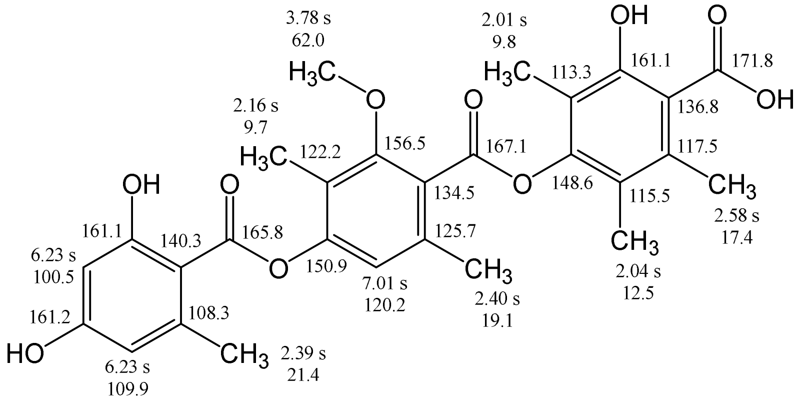

White powder, UV (CAN) λ_{max}: 213.4, 267.8, 307.0 nm; HRESIMS: *m/z*: 525.1778 $[M+H]^+$ (calcd for $C_{28}H_{29}O_{10}$, 525.1755); ^1H NMR and ^{13}C NMR (500 and 125 MHz, DMSO-d_6) [52].

Thielavin Z6 (79)

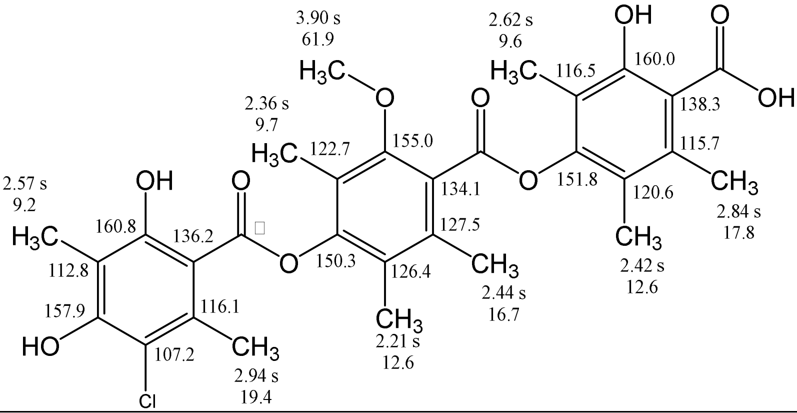

White powder; UV (CAN) λ_{max}: 215.8, 276.1, 307.0 nm; HRESIMS: *m/z* 587.1719/589.1646 $[M+H]^+$ 3:1 (calcd for $C_{30}H_{32}ClO_{10}$, 587.1679); ^{1}H NMR and ^{13}C NMR (500 and 125 MHz, DMSO-*d*6) [52].

3-Hydroxy-2,5-dimethylphenyl 4-[(2,4-dihydroxy-3,6-dimethylbenzoyl)oxy]-2-hydroxy-3,6-dimethylbenzoate (80)

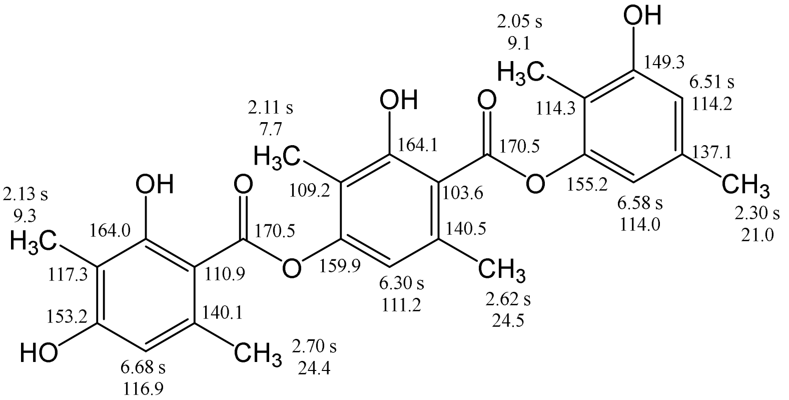

UV (MeOH) λ_{max}: 226 (4.39), 278 (4.13), 310 (3.81); IR (KBr) γ_{max}: 3419, 2925, 1664, 1623, 1583, 1147, 1257 cm^{-1}; ESIMS: *m/z* 465 $[M-H]^-$, 301 (25), 163 (100); ESITOFMS: *m/z* 465.1522 $[M-H]^-$ (calcd for $C_{26}H_{25}O_8$, 465.1548); ^{1}H and ^{13}C NMR (400 and 100 MHz, $CDCl_3$ and CD_3OD) [49].

3-Hydroxy-2,4,5-trimethylphenyl 4-[(2,4-dihydroxy-3,6-dimethylbenzoyl)oxy]-2-hydroxy-3,6-dimethylbenzoate (81)

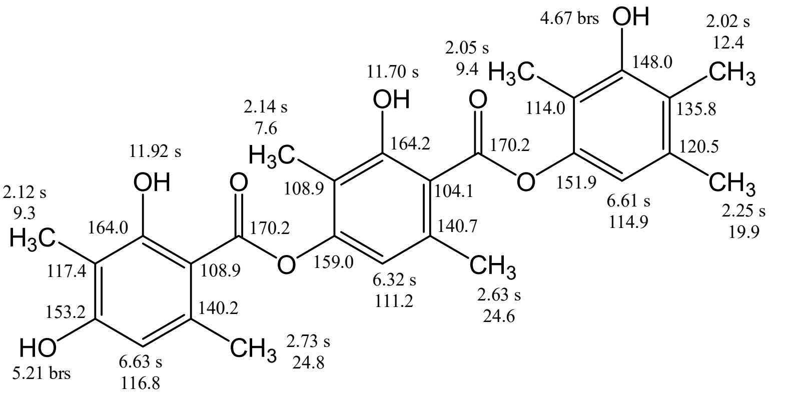

White amorphous powder; UV λ_{max}: 226 (4.37), 278 (4.12), 310 (3.82) nm; IR (KBr) γ_{max}: 3421, 2931, 1706, 1654, 1623, 1585, 1143, 1257 cm^{-1}; ESIMS: *m/z* 479 $[M-H]^-$, 315 (20), 163 (100); ESI-TOFMS: *m/z* 479.1674 $[M-H]^-$ (calcd for $C_{27}H_{27}O$, 479.1705); ^{1}H and ^{13}C NMR (400 and 100 MHz, $CDCl_3$ and CD_3O [49].

Gyrophoric acid (82)

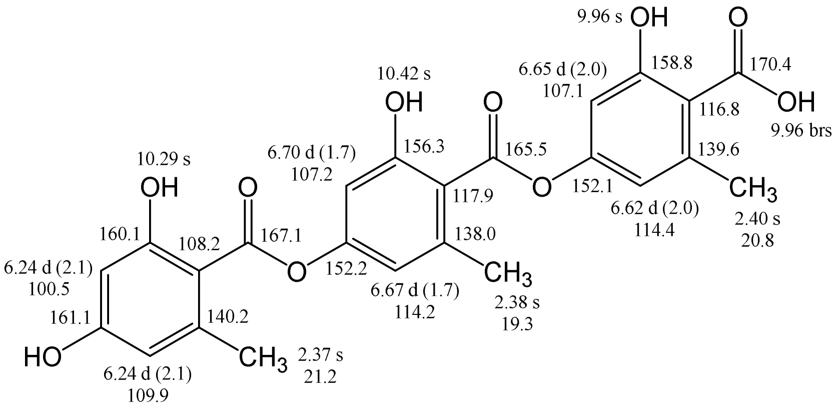

Colorless materials; ^{1}H and ^{13}C NMR (500 and 125 MHz, DMSO-*d*6) [71,72].

**Trivaric acid (83)**	
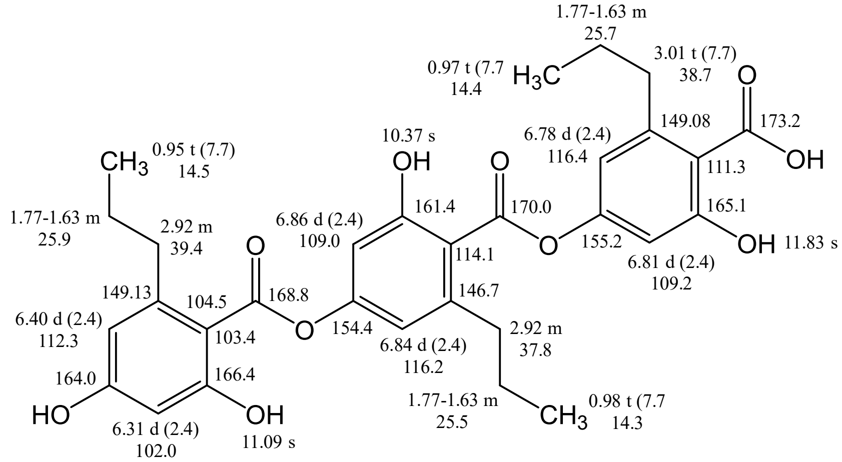	White amorphous powder; ESIMS: *m/z* 551 [M-H]-; ^{1}H NMR and ^{13}C NMR (500 and 125 MHz, acetone-*d*6) [25].
**Cytonic acid A (84)**	
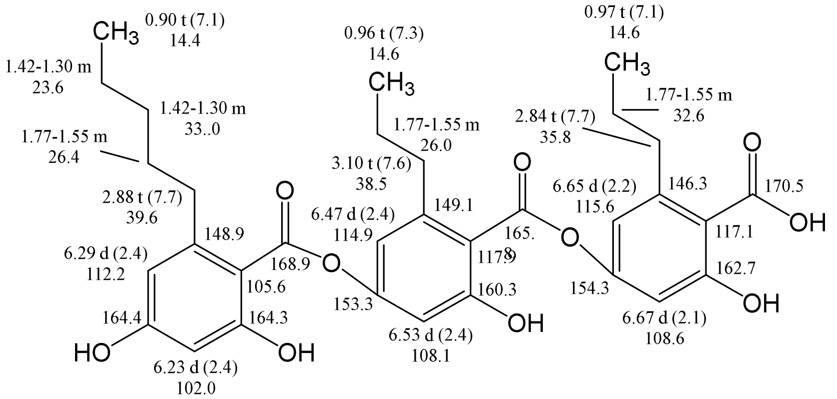	White powder; UV (MeOH) λ_{max} (log *ε*): 211 (4.7), 253 (4.1), 284 (3.9) nm; IR (KBr) γ_{max}: 3450, 1766, 1762, 1651, 1575, 1557, 1538, 1135, 1035, 824 cm^{-1}; (-) ESIMS *m/z*: 579 [M-H]- (2.2%), 195 (100%), 177 (54.1%), 401 (42.2%), 151 (32.4%), 205 (26.8%), 133 (14.6%), 223 (15.5%), 373 (9.2%); (-) ESIMS: *m/z* 579: 579, 401, 373, 223, 195; (-) ESIMS: *m/z* 401: 195, 223, 401; ESIMS-MS: *m/z* 373, 195, 373; (-)ESIMS: *m/z* 223, 223, 179; (-) ESIMS: *m/zr* 195, 151, 195; (-) APCIMS: *m/z* 151 (100%), 195 (91.5%), 179 (48.3%), 137 (31.3%), 177 (22.5%), 223 (16.2%), 205 (18.6%); (-) HRMS: *m/z* 579.2214 [M-H]- (calcd for $C_{32}H_{35}O_{10}$, 579.2227) (^{1}H and ^{13}C NMR (400 and 100 MHz, CD_3OD) [73].
**Cytonic acid B (85)**	
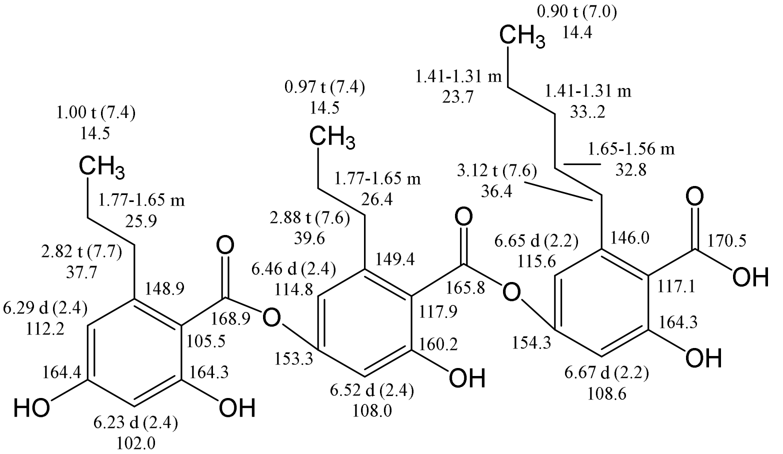	White powder; UV (MeOH) λ_{max} (log *ε*): 212 (4.7), 250 (4.1), 286 (3.9) nm; IR (KBr) γ_{max}: 3450, 1765, 1760, 1651, 1574, 1557, 1538, 1137, 1034, 828 cm^{-1}; (-) ESIMS: *m/z* 579 [M-H]- (2.0%), 223 (100%), 401 (70.3%), 177 (52.7%), 133 (8.1%); (-) ESIMS: *m/z* 579: 579, 401, 223, 195, 373, 151; (-) ESIMS: *m/z* 401, 401, 223, 195, 177; (-) ESIMS: *m/z* 373, 195, 177, 151; (-) ESIMS: *m/z* 223, 179; (-) APCIMS: *m/z* 223 (100%), 177 (100%), 179 (75.5%), 133 (70.6%), 401 (2.7%); ^{1}H and ^{13}C NMR (400 and 100 MHz, CD_3OD) [73].
**Amidepsine D (86)**	

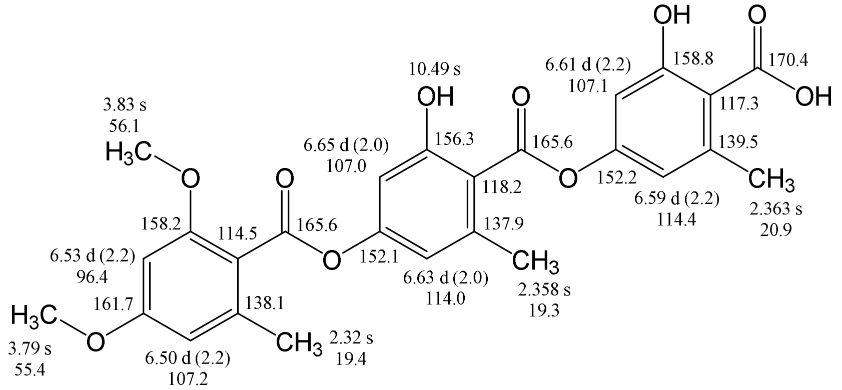

White powder; [α]$_D^{25}$ 0.0 (*c* 0.1, MeOH); UV (MeOH) λ_{max} (ε): 217 (30700), 250 (17700), 294 (8900) nm; IR (KBr) γ_{max}: 1668, 1608, 1419, 1246, 1200, 1140 cm^{-1}; FAMMS: *m/z* 497 [M+H]$^+$, 519 [M+Na]$^+$, 495 [M-H]$^-$; HRFABMS 495.1294 [M-H]$^-$ (cald for $C_{26}H_{23}O_{10}$, 495.1291) ^{1}H and ^{13}C NMR (400 and 100 MHz, DMSO-d_6) [7475].

Amidepsine K (87)

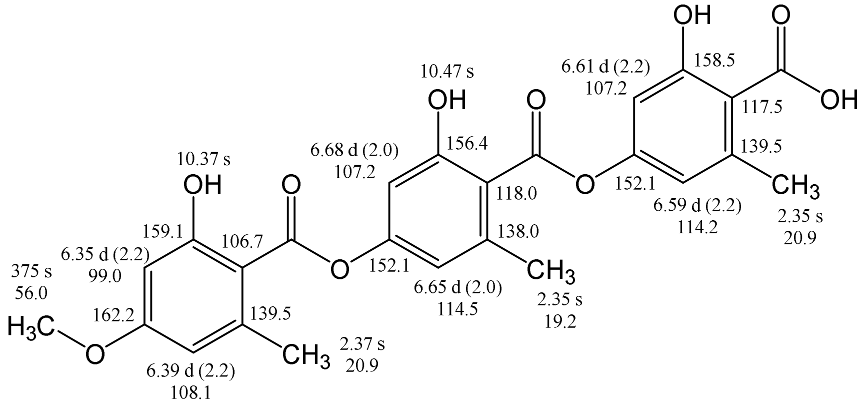

White powder; [α]$_D^{25}$ 0.0 (*c* 0.1, MeOH); UV (MeOH) λ_{max} (ε): 213 (38500), 270 (7900) nm; IR (KBr) γ_{max}: 2979, 2935, 2852, 1662, 1610, 1581, 1421, 1248, 1201, 1148 cm^{-1}; FABMS: *m/z* 483 [M+H]$^+$, 505 [M+Na]$^+$, 481 [M-H]$^-$; HRFABMS: *m/z* 483.1274 [M-H]$^-$ (cald for $C_{25}H_{32}O_{10}$, 483.1291); ^{1}H and ^{13}C NMR (600 and 125 MHz, DMSO-d_6) [71].

Amidepsine A (88)

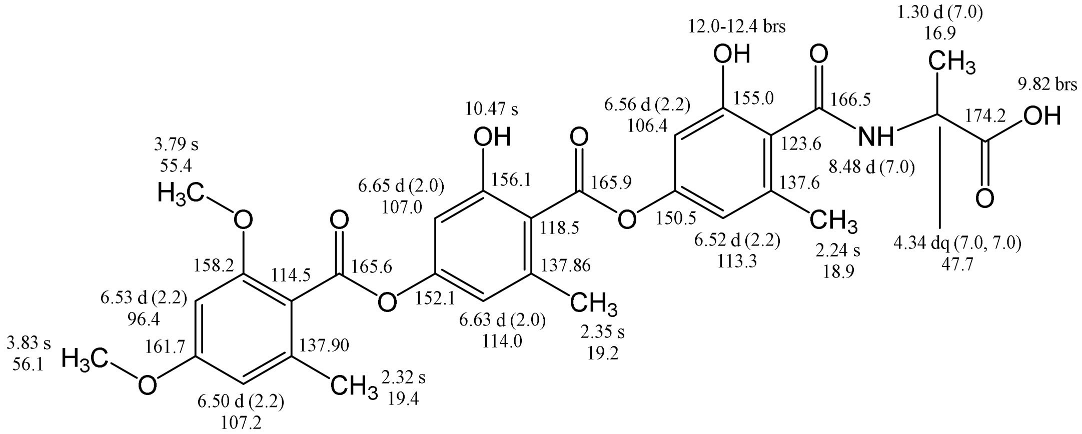

Pale yellow powder; [α]$_D^{25}$ - 10.0 (*c* 0.1, MeOH); UV (MeOH) λ_{max} (ε): 218 (35000), 254 (18000), 282 (10600) nm; IR (KBr) γ_{max}: 1660, 1583, 1506, 1410, 1246, 1136 cm^{-1}; FAMMS *m/z*: 568 [M+H]$^+$, 590 [M+Na]$^+$, 566 [M-H]$^-$; HRFABMS 566.1662 [M-H]$^-$ (cald for $C_{29}H_{28}O_{11}N$, 566.1674) ^{1}H and ^{13}C NMR (400 and 100 MHz, DMSO-d_6) [74,75].

86- Amidepsine B (89)

<table>
<tr><td colspan="2">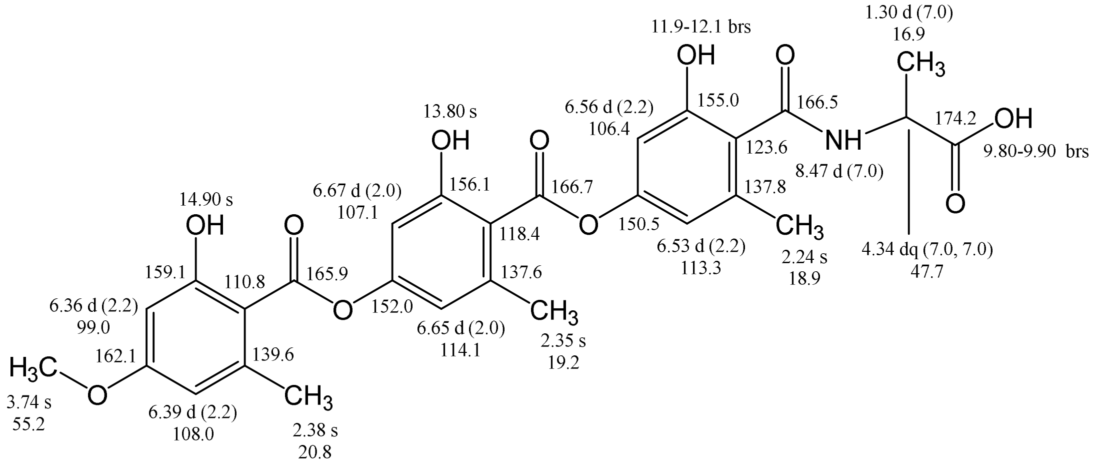
</td></tr>
<tr><td colspan="2">Pale yellow powder; [α]D25 -16.0 (c 0.1, MeOH); UV (MeOH) λ_{max} (ε): 216 (34000), 266 (22160), 305 (11100) nm; IR (KBr) γ_{max}: 1656, 1583, 1400, 1248, 1163, 1144 cm^{-1}; FAMMS m/z: 554 $[M+H]^+$, 576 $[M+Na]^+$, 552 $[M-H]^-$; HRFABMS: m/z 552.1506 $[M-H]^-$ (cald for $C_{28}H_{26}O_{11}N$, 552.1529); ^1H and ^{13}C NMR (400 and 100 MHz, DMSO-d_6) [74 64,75].</td></tr>
<tr><td colspan="2">Amidepsine B (-): White powder; mp 153-156 °C; $[\alpha]^{29}_D$ -17.3 (c 0.1, CH_3OH); IR (KBr) γ_{max}: 3087, 2937, 2858, 1668, 1610, 1454 cm^{-1}; ^1H NMR (270 MHz, $CDCl_3$): δ_H 10.4 (brs, 1H), 8.44 (d, J = 6.8 Hz, 1H), 6.68 (brs, 1H), 6.66 (brs, 1H), 6.56 (brs, 1H), 6.53 (brs, 1H), 6.39 (brs, 1H), 6.36 (brs, 1H), 4.35 (m, 1H), 3.74 (s, 3H), 2.45 (s, 3H), 2.38 (s, 3H), 2.35 (s, 3H), 1.31 (d, J = 7.1 Hz, 3H); ^{13}C NMR (75 MHz, $CDCl_3$) δ_C 174.1, 166.7, 166.4, 165.8, 162.1, 159.1, 156.1, 155.0, 152.0, 150.5, 139.6, 137.8, 137.6, 123.5, 118.4, 114.1, 113.3, 110.7, 108.1, 107.1, 106.4, 99.0, 55.2, 47.6, 20.8, 19.2, 18.8 and 16.9; HRFABMS: m/z 552.1510 $[M-H]^-$ (calcd for $C_{28}H_{26}NO_{11}$, 552.1506) [79].</td></tr>
<tr><td colspan="2">Amidepsine B (+): White powder; mp 156-159 °C; $[\alpha]^{25}_D$ +10.4 (c 0.1, CH_3OH); IR (KBr) γ_{max}: 3087, 2937, 2858, 1668, 1610, 1454 cm^{-1}; ^1H NMR (270 MHz, $CDCl_3$): δ_H 10.4 (brs, 1H), 8.44 (d, J = 6.8 Hz, 1H), 6.68 (brs, 1H), 6.66 (brs, 1H), 6.56 (brs, 1H), 6.53 (brs, 1H), 6.39 (brs, 1H), 6.36 (brs, 1H), 4.35 (m, 1H), 3.74 (s, 3H), 2.45 (s, 3H), 2.38 (s, 3H), 2.35 (s, 3H), 1.31 (d, J = 7.1 Hz, 3H); ^{13}C NMR (75 MHz, $CDCl_3$): δ_C 174.1, 166.7, 166.4, 165.8, 162.1, 159.1, 156.1, 155.0, 152.0, 150.5, 139.6, 137.8, 137.6, 123.5, 118.4, 114.1, 113.3, 110.7, 108.1, 107.1, 106.4, 99.0, 55.2, 47.6, 20.8, 19.2, 18.8 and 16.9; HRFABMS: m/z 554.1663 $[M+H]^+$ (calcd for $C_{28}H_{28}NO_{11}$, 554.1662) [79].</td></tr>
<tr><td colspan="2">Amidepsine C (90)</td></tr>
<tr><td>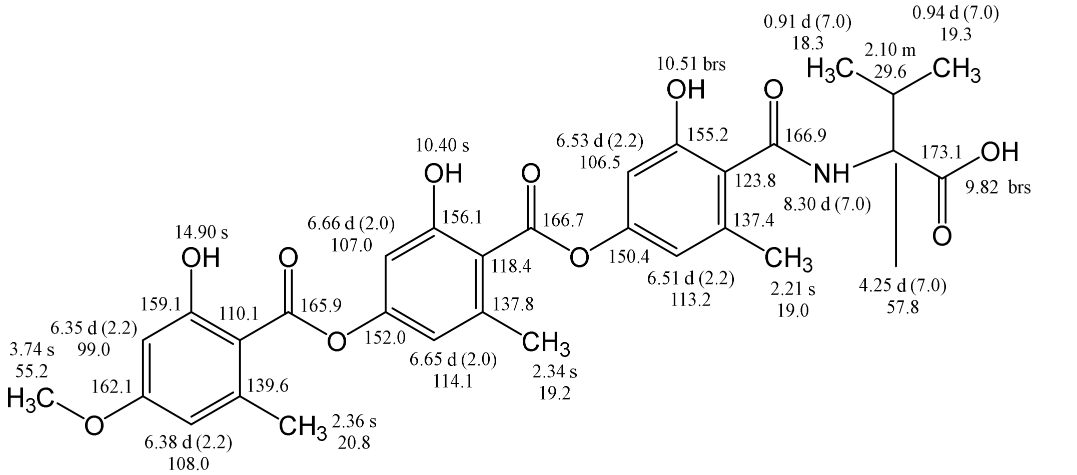
</td><td>Pale yellow powder; [α]D25 -94.0 (c 0.1, MeOH); UV (MeOH) λ_{max} (ε): 209 (37400), 269 (24200), 306 (12300) nm; IR (KBr) γ_{max}: 1670, 1612, 1583, 1313, 1248, 1140 cm^{-1}; FAMMS: m/z 582 $[M+H]^+$, 604 $[M+Na]^+$, 580 $[M-H]^-$; HRFABMS: m/z 580.1822 (cald for $C_{30}H_{30}O_{11}N$, 580.1819) ^1H and ^{13}C NMR (400 and 100 MHz, DMSO-d_6) [74,75].</td></tr>
<tr><td colspan="2">Amidepsine E (91)</td></tr>
</table>

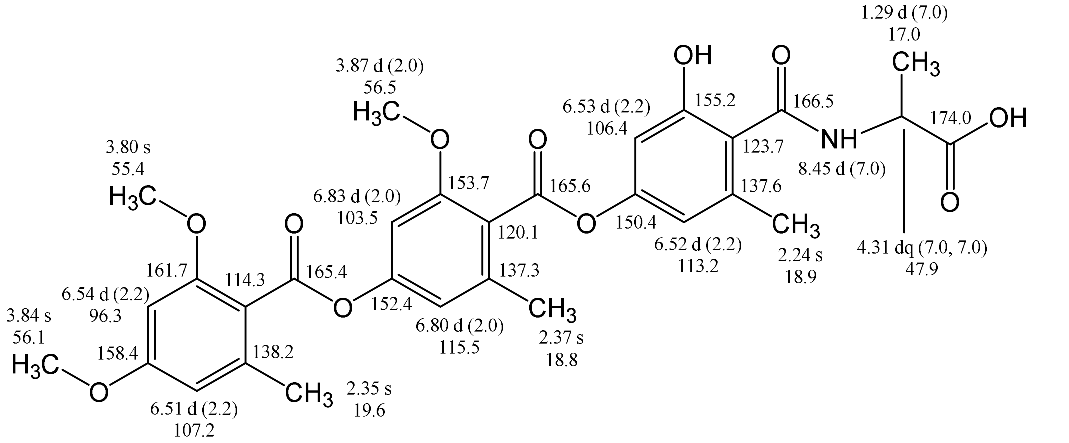	Pale yellow powder; mp 215 °C; $[\alpha]_D^{28}$ -7.0 (*c* 0.1, MeOH); λ_{max} (ε): 206 (75500), 246 (14000), 281 (10400) nm; IR (KBr) γ_{max}: 3410, 2927, 1732, 1597, 1462, 1327, 1250, 1139 cm^{-1}; FABMS *m/z*: 582 $(M+H)^+$, 604 $(M+Na)^+$, 580 $(M-H)^-$; HRFABMS: *m/z* 582.1977 (cald for $C_{30}H_{32}O_{11}N$, 582.1975); ^1H and ^{13}C NMR (400 and 100 MHz, DMSO-d_6) [76].
**Amidepsine F (92)**	
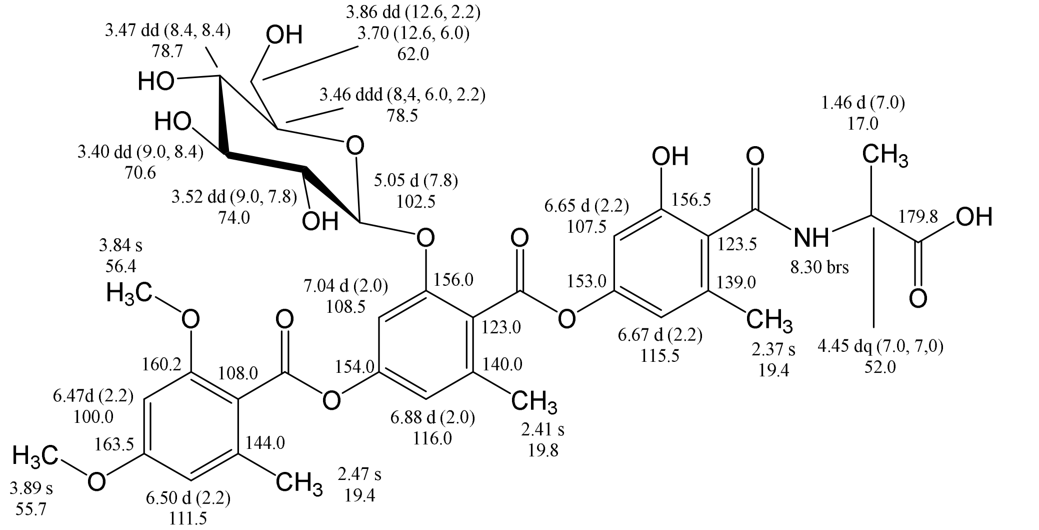	Pale yellow powder; $[\alpha]_D^{25}$ -10.0 (*c* 0.1, MeOH); UV (MeOH) λ_{max} (ε): 204 (48000), 281 (5600) nm; IR (KBr) γ_{max}: 3379, 2968, 2956, 2927, 2858, 1739, 1599, 1456, 1257, 1038 cm^{-1}; FABMS: *m/z* 730 $[M+H]^+$, 752 $[M+Na]^+$, 728 $[M-H]^-$; HRFABMS: *m/z* 730.2307 (cald for $C_{35}H_{40}O_{16}N$, 730.2347); ^1H and ^{13}C NMR (600 and 125 MHz, CD_3OD) [71].
**Amidepsine G (93)**	
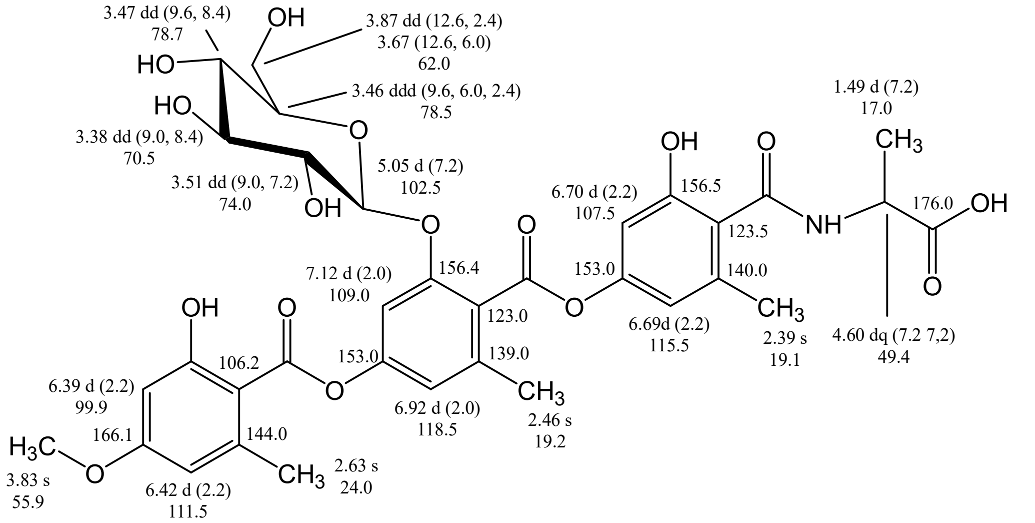	Pale yellow powder; $[\alpha]_D^{25}$ -13.0 (*c* 0.1, MeOH); UV (MeOH) λ_{max} (ε): 203 (50500), 270 (15500) nm; IR (KBr) γ_{max}: 3311, 2929, 2860, 1738, 1657, 1603, 1450, 1657, 1603, 1450, 1254, 1157, 1063 cm^{-1}; FABMS: *m/z* 716 $[M+H]^+$, 738 $[M+Na]^+$, 714 $[M-H]^-$; HRFABMS: *m/z* 716.2146 (cald for $C_{34}H_{38}O_{16}N$, 716.2191); ^1H and ^{13}C NMR (600 and 125 MHz, CD_3OD) [71].
**Amidepsine H (94)**	

<table>
<tr><td>
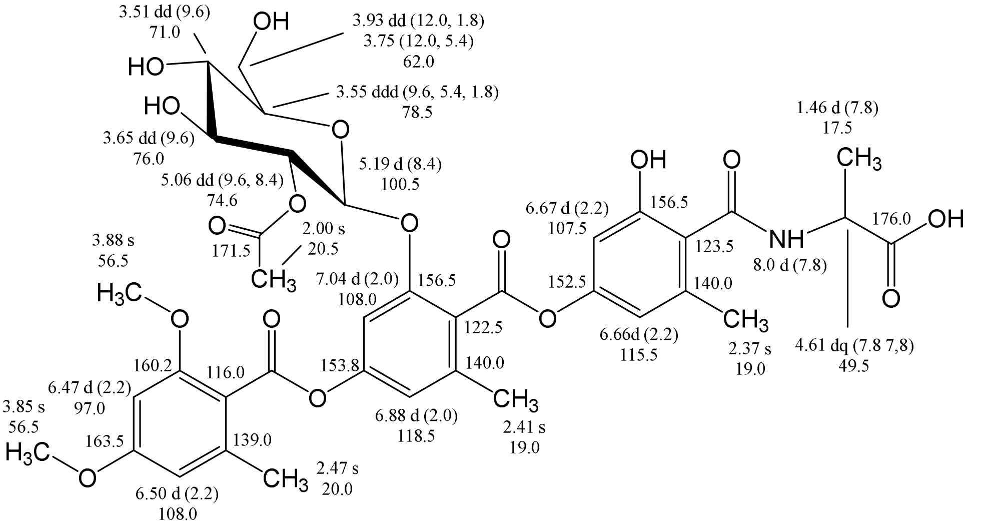

</td><td>Pale yellow powder; [α]$_D^{25}$ -19.0 (*c* 0.1, MeOH); UV (MeOH) λ_{max} (ε): 203 (59500), 251 (11700) nm; IR (KBr) γ_{max}: 3359, 2956, 2927, 2858, 1741, 1599, 1454, 1254, 1157, 1038 cm^{-1}; FABMS: *m/z* 772 [M+H]$^+$, 793 [M+Na]$^+$, 770 [M-H]$^-$; HRFABMS: *m/z* 772.2405 (cald for $C_{37}H_{42}O_{17}N$, 772.2453) ^{1}H and ^{13}C NMR (600 and 125 MHz, CD_3OD) [71].</td></tr>
<tr><td colspan="2">Amidepsine I (95)</td></tr>
<tr><td>
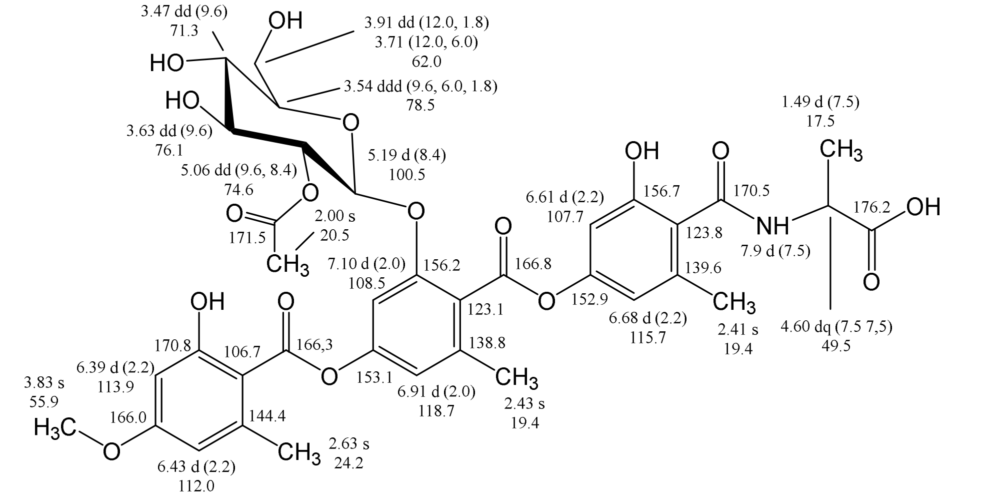

</td><td>Pale yellow powder; [α]$_D^{25}$ -21.0 (*c* 0.1, MeOH); UV (MeOH) λ_{max} (ε): 203 (49400), 270 (14700) nm; IR (KBr) γ_{max}: 3355, 2968, 2931, 1745, 1655, 1616, 1448, 1252, 1157 cm^{-1}; FABMS: *m/z* 758 [M+H]$^+$, 780 [M+Na]$^+$, 756 [M-H]$^-$; HRFABMS: *m/z* 758.2296 (cald for $C_{36}H_{40}O_{17}N$, 758.2313); ^{1}H and ^{13}C NMR (600 and 125 MHz, CD_3OD) [71].</td></tr>
<tr><td colspan="2">Amidepsine J (96)</td></tr>
<tr><td>
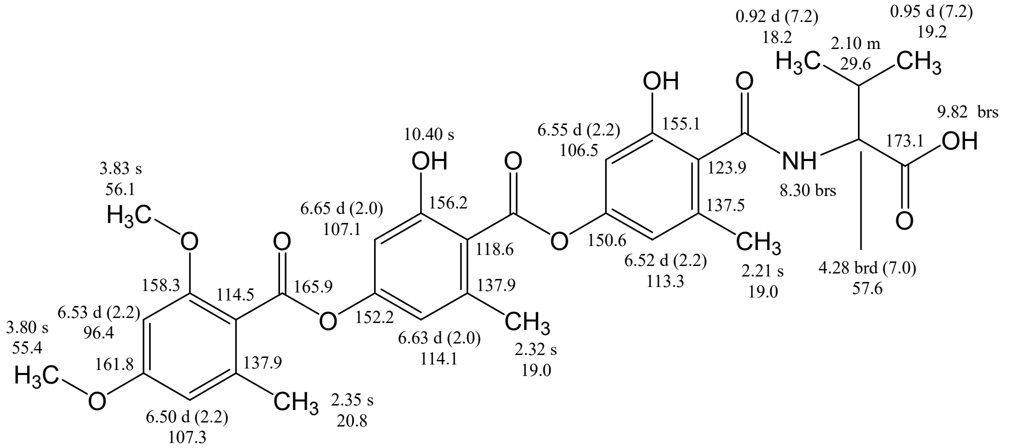

</td><td>Pale yellow powder; [α]$_D^{25}$ +3.0 (*c* 0.1, MeOH); UV (MeOH) λ_{max} (ε): 204 (50200), 256 (11500) nm; IR (KBr) γ_{max}: 3172, 2964, 2929, 2862, 1741, 1670, 1600, 1456, 1248, 1136, 1030 cm^{-1}; FABMS: *m/z* 596 [M+H]$^+$, 618 [M+Na]$^+$, 594 [M-H]$^-$; HRFABMS: *m/z* 596.2098 [M+H]$^+$ (cald for $C_{31}H_{34}O_{11}N$, 596.2132); ^{1}H and ^{13}C NMR (600 and 125 MHz, DMSO-d_6) [71].</td></tr>
<tr><td colspan="2">CJ-21,164 (97)</td></tr>
</table>

<table>
<tr><td>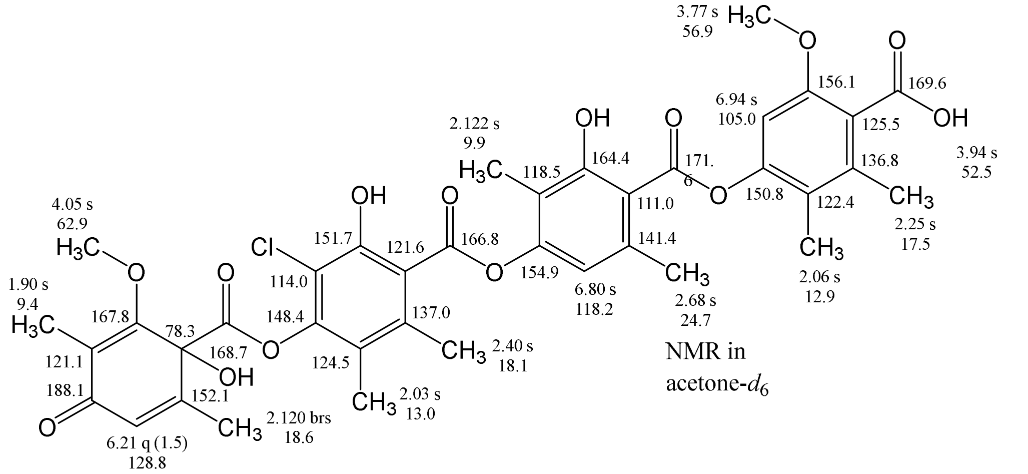
</td><td rowspan="2">White powder; UV (MeOH) λ_{max} (ε): 215 (78800), 252 (29900), 284 (12700), 315 (sh) nm; $[\alpha]^{24}_D$ +63.8 (*c* 1.00, MeOH); IR (KBr) γ_{max}: 3700-2500, 3408, 3250 (sh), 2937, 2937, 1760, 1725 (sh), 1676, 1618, 1583, 1445, 1385, 1348, 1296, 1258, 1190, 1140, 1084, 959, 881, 723 cm^{-1}; HRFABMS: *m/z* 751.1782 $[M-H]^-$ (calcd for $C_{38}H_{36}ClO_{14}$, 551.1794); LRFABMS: *m/z* 753/755 (5:2), 557/559, 393/395, 363/365, 359/361, 199/201, 195, 179, 168, 165; ^{1}H and ^{13}C NMR (270 and 68 MHz, acetone-d_6 and CD_3OD) [77].</td></tr>
<tr><td>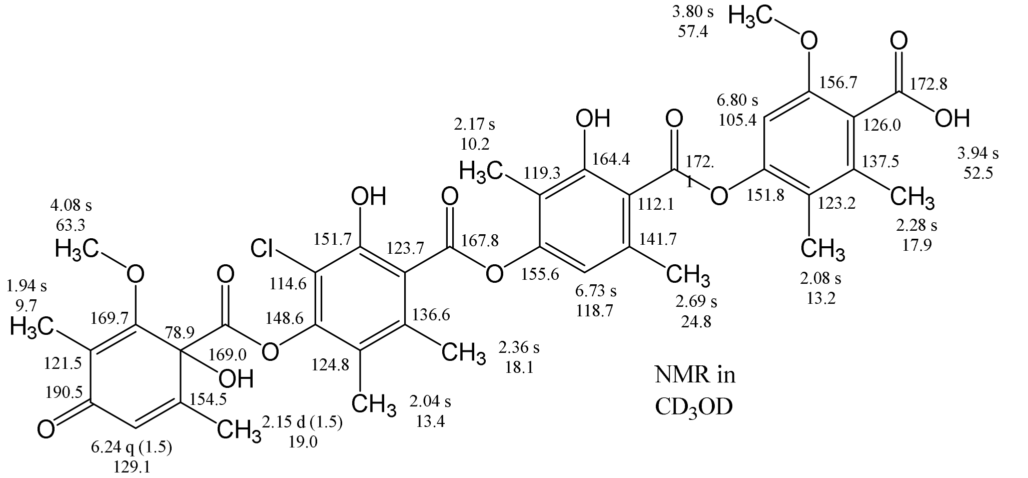
</td></tr>
<tr><td colspan="2">Thielocin A1α (**98**)*</td></tr>
<tr><td colspan="2">Colorless powder; mp 203-206°C; HR-MS: *m/z* 997.3861 (calcd 997.3854, $C_{54}H_{60}O_{18}$); IR (KBr) γ_{max}: 3490, 1740, 1675, 1577, 1155, 1095 cm^{-1} [90]; ^{1}H NMR (400 MHz, $CDC1_3$-CD_3OD (20:1): δ_H 2.74 (1H, d, *J* =16 Hz), 3.3 (1H, d, *J* = 16Hz)), 3.83 (9H, s, 3×OCH_3), 3.35 (3H, s, OCH_3), 1.33 (3H, s), 1.89 (3H, s), 2.23 (3H, s), 2.24 (3H, s), 2.26 (3H, s), 2.27 (3H, s), 2.40 (3H, s), 2.41 (3H, s), 2.27 (6H, s), 2.30 (3H, s), 2.32 (6H each, s) [78].</td></tr>
<tr><td colspan="2">Thielocin A1β (**99**)*</td></tr>
<tr><td colspan="2">Colorless powder; mp 203-206°C; HR-MS: *m/z* 997.3865 (calcd 997.3854, $C_{54}H_{60}O_{18}$); IR (KBr) γ_{max}: 3440, 1744, 1675, 1575, 1155, 1095 cm^{-1} [90]. ^{1}H NMR (400 MHz, $CDC1_3$-CD_3OD (20:1): δ_H 2.72 (1H, d, *J* =16 Hz), 3.38 (1H, d, *J* = 16Hz), 3.78 (3H, s, OCH_3), 3.81 (3H, s, OCH_3), 3.82 (9H, s, 3×OCH_3), 1.52 (3H, s), 1.75 (3H, s), 2.13 (3H, s), 2.20 (3H, s), 2.21 (3H, s), 2.22 (3H, s), 2.25 (3H, s), 2.34 (3H, s), 2.37 (3H, s), 2.40 (3H, s), 2.24 (6H, s), 2.28 (6H each, s) [78].</td></tr>
<tr><td colspan="2">Thielocin A2α (**100**)*</td></tr>
<tr><td colspan="2">Colorless powder; mp 203-206°C; HR-MS: *m/z* 1175.4479 (calcd 1175.4483, $C_{64}H_{70}O_{21}$); UV (MeOH+ dil HCl-MeOH) λ_{max} (ε): 274.6 (25,100), 316.7 (5,380) nm; UV (dil NaOH-MeOH) λ_{max} (ε): 260 (sh), 323.3 (52,800) nm; IR (KBr) γ_{max}: 3440, 1740, 1605, 1574, 1150, 1090 cm^{-1} [75].</td></tr>
<tr><td colspan="2">Thielocin A2β (**101**)*</td></tr>
<tr><td colspan="2">Colorless powder; mp 204-206°C; HR-MS: *m/z* 1175.4488 (calcd 1175.4483, $C_{64}H_{70}O_{21}$); UV (MeOH+ dil HCl-MeOH) λ_{max} (ε): 274.5 (22,900, sh), 321 (4,520) nm; UV (dil NaOH-MeOH) λ_{max} (ε): 260 (sh), 323 (51,300) nm; IR (KBr) γ_{max}: 3420, 1745, 1605, 1572, 1147, 1090 cm^{-1} [75].</td></tr>
<tr><td colspan="2">Thielocin A3 (**104**)*</td></tr>
<tr><td colspan="2">Colorless powder; mp 202-205°C; HR-MS: *m/z* 1175.4488 (calcd 1175.4470, $C_{64}H_{70}O_{21}$); UV (MeOH+ dil HCl-MeOH) λ_{max} (ε): 274.5 (22,512), 318.6 (5,232) nm; UV (dil NaOH-MeOH) λ_{max} (ε): 255 (sh), 323 (29,825) nm; IR (KBr) γ_{max}: 3440, 1743, 1657, 1608, 1576, 1097 cm^{-1} [75].</td></tr>
<tr><td colspan="2">Thielocin B1 (**105**)*</td></tr>
<tr><td colspan="2">Colorless powder; mp 175-182°C; HR-MS: *m/z* 967.3754 (calcd 967.3749, $C_{53}H_{58}O_{17}$); UV (MeOH+dil HCl-MeOH) λ_{max} (ε): 275 (24,150), 323 (5,120) nm; UV (dil NaOH-MeOH) λ_{max} (ε): 248 (21,250, sh), 331 (32,650); IR (KBr) γ_{max}: 3424, 1742, 1659, 1610, 1152, 1096 cm^{-1} [75].</td></tr>
</table>

Thielocin B2 (**106**)*
Colorless powder; mp 180-186°C; HR-MS: *m*/*z* 967.3751 (calcd 967.3749, $C_{53}H_{58}O_{17}$); UV (MeOH+dil HCl-MeOH) λ_{max} (ε): 245 (24,830 sh), 325 (3,579) nm; IR (KBr) γ_{max}: 3440, 1775, 1734, 1665, 1610, 1150 cm^{-1} [75 121].
Thielocin B3 (**107**)*
Colorless powder; mp 194-197°C; HR-MS: *m*/*z* 1131.4226 (calcd 1131.4226, $C_{62}H_{66}O_{20}$); UV (MeOH+ dil HCl-MeOH) λ_{max} (ε): 276 (39,760), 306 (11,690 sh) nm; IR (KBr) γ_{max}: 3410, 1740, 1650, 1610, 1143, 1094 cm^{-1} [75].

* The spectral data were published in Japanese language [75].

References

120. Nazir, M.; Sultan, M.; Riaz, N.; Hafeez, M.; Hussain, H.; Ahmed, I.; Schulz, B.; Draeger, S.; Jabbar, A.; Krohn, K. Depsitinuside: A new depside galactoside from an endophytic fungus isolated from *Viburnum tinus*. *J. Asian. Nat. Prod. Res.* **2011**, *13*, 1056–1060.
121. Narui, T.; Sawada, K.; Takatsuki, S.; Okuyama, T.; Culberson, C.F.; Culberson, W.L.; Shibata, S. NMR assignments of depsides and tridepsides of the lichen family Umbilicariaceae. *Phytochemistry* **1998**, *48*, 815–822.
